# Prioritization of research engaged with rare disease stakeholders: a systematic review and thematic analysis

**DOI:** 10.1186/s13023-023-02892-2

**Published:** 2023-11-23

**Authors:** Soho Yoon, Minjee Lee, Hoi-In Jung, M. Mahmud Khan, So-Yoon Kim, Hannah Kim, Sophia Wasti

**Affiliations:** 1 Asian Institute for Bioethics and Health Law, Yonsei University, Seodaemun–gu, Seoul Republic of Korea; 2 Simons Cancer Institute, Southern Illinois University School of Medicine, Springfield, IL USA; 3 Preventive Dentistry and Public Oral Health, Yonsei University College of Dentistry, Seodaemun-gu, Seoul, Republic of Korea; 4 Department of Health Policy and Management, College of Public Health, University of Georgia, Athens, GA USA; 5 College of Medicine, Yonsei University, Seodaemun-gu, Seoul, Republic of Korea

**Keywords:** Rare diseases, Stakeholders, Survey, Patients, Caregivers, Healthcare professionals, Political support, Policy, Public funding

## Abstract

**Background:**

Although rare diseases (RD) are increasingly becoming a priority for healthcare activities and services around the world, developing research policy for investigating RD in public settings proves challenging due to the limited nature of existing evidence. Rare conditions require the involvement of a wide range of stakeholders in order to promote general awareness and garner political support. Consequently, it is critically important to identify trends in the various types of research focusing on rare disease stakeholders, including the specific topics or issues to be included in surveys and studies focused on RD stakeholders. This systematic review and thematic analysis analyses the existing literature based on RD surveys, including the stakeholders involved, and proposes potential research priorities and initiatives for policy-making related to RD.

**Methods:**

Articles were downloaded and analyzed from across five electronic databases (PubMed, EMBASE, Cochrane Central, Web of Science, and CINHAL) and 115 studies were included.

**Results:**

Across 115 studies, the main research participants were patients and/or caregivers (n = 77, 67.0%), health professionals (n = 18, 15.7%), and the public (n = 7, 6.1%). The studies discussed RDs in general (n = 46, 40.0%), endocrine, nutritional, and metabolic diseases (n = 20, 17.4%) and other RDs. Experiences with RD were examined by more than half of the selected studies (n = 74, 64.3%), followed by the opinions of stakeholders (n = 24, 20.9%). Most of the studies used surveys in order to collect relevant data (n = 114, 99.1%). Additionally, the majority of the studies were conducted in high-income countries (n = 92, 80.0%) and rarely in middle and low-income countries (n = 12, 13.8%).

**Conclusion:**

Stakeholder research on RD reveals that there are significant instances of unmet needs and various challenges faced by the medical system in dealing with RDs.

Furthermore, public awareness and support is critical to ensuring political feasibility of increasing national-level investments for RDs and development of medical products and treatment.

**Supplementary Information:**

The online version contains supplementary material available at 10.1186/s13023-023-02892-2.

## Introduction

Approximately 400 million people worldwide are affected by rare diseases (RD) [1]. It is extremely difficult to diagnose RD at an early stage due to significantly limited number of cases and healthcare providers’ general inexperience with these types of diseases. The fact that case numbers are generally very low also impedes the development of cost-effective, viable medications and treatments [2]. Given the low market demand for RD-related medical products and treatments, private investments in innovation and development continue to fall short of socially desirable levels. This highlights the crucial role of public sector funding in acting as a catalyst for increased private funding. Furthermore, RDs exert significant strains on individuals and society, impacting social well-being, causing economic burdens, and leading to uncertain health outcomes [3]. Consequently, it is necessary to address the current constraints preventing progress in the field of RD treatment and research, including the shortage of RD specialists and limited accessibility to relevant information. Overcoming these challenges is vital for meeting the clinical and social needs of RD patients, safeguarding their rights, supporting caregivers, and ultimately reducing the disease burden [4].

The exchange of information between RD stakeholders, including health professionals, patient organizations, healthcare centers, government policymakers, and researchers, has been widely recognized as important for improving understanding and awareness of factors relevant to RD [5]. These exchanges play a crucial role in enhancing knowledge and awareness regarding various aspects of RD, such as diagnosis and treatment strategies, improving quality of life (QoL), and identifying unmet needs across stakeholders. This shared information can then be used to inform and motivate political actions aimed at addressing the needs of RD patients and healthcare providers. For example, the European Union (EU) adopted a landmark RD document, the Communication on Rare Diseases: Europe’s Challenges, in 2008 [6], using EurordisCare’s longitudinal questionnaires and a public hearing to encourage effective RD prevention, diagnosis, and treatment. Although various stakeholders are interested in improving the QoL of RD patients as well as improving access to diagnosis and treatment options, there is no consensus on what constitutes best practice in dealing with RD’s or the most effective approaches to priority setting in research and development related to RD’s. Rare conditions require involvement of a wider array of stakeholders to create awareness and political support. It is therefore critically important to identify the relevant stakeholders and specific topics or issues to be included in RD stakeholder surveys. These surveys can help to identify the challenges of diagnosing and treating RD’s, as well as the barriers to accessing care for people with rare diseases. Without the wider involvement of these stakeholders, RD-related issues will continue to remain neglected and will not get the priority they deserve.

This systematic review synthesizes literature on RD and proposes feasible priorities for future research. Specifically, it considers the following research questions: (1) What types of stakeholders have been invited to participate in RD stakeholder surveys? (2) Which study topics have been covered in RD stakeholder surveys? and (3) How have RD stakeholder surveys been conducted? The results of this review will hopefully provide better insight for designing surveys relevant to RD research that can guide political priorities on this topic.

## Methods

### Overview

This systematic review utilized the Preferred Reporting Items for Systematic Reviews and Meta-Analyses (PRISMA) Statement for article selection and was guided by a multidisciplinary team comprising of academic researchers, professors, medical doctors, and a medical librarian. Furthermore, a thematic analysis was conducted to analyze the study topics in-depth.

### Search strategy and data extraction

**Fig. 1:**
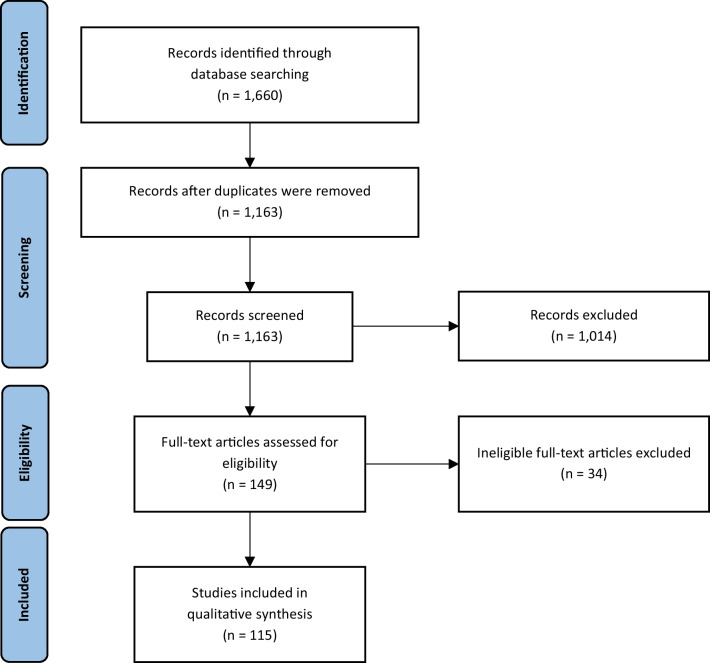
Flowchart indicating the selection of studies for the review - 115 studies were selected for inclusion

Five electronic databases (PubMed, EMBASE, Cochrane Central, Web of Science, and CINHAL) were used to find the relevant literature and the search was conducted from May 6, 2020, to May 20, 2020. The authors designed and agreed on the keywords and medical subject headings (MeSH) to use for the search (Table 1). Eight exclusion criteria were designed to narrow the search focus and exclude articles that were not relevant to the research questions (Additional file 1: Table S2). Titles and abstracts were screened by the research team to make sure they satisfied the relevant exclusion and inclusion criteria. During the evaluation process, a number of studies were excluded based on the following exclusion criteria. Six studies (0.6%) were eliminated because they were not written in the English language. An additional 106 studies (10.1%) were excluded as they focused on non-rare diseases, and 55 studies (5.3%) were not considered due to the fact that they involved non-human stakeholders. 69% (722 studies) were not included due to being published in non-peer-reviewed journals. Across these various exclusions, 456 were conference/meeting abstracts, 66 were comment/review/editorial/dissertation papers, 72 were case reports, 125 were clinical papers, and 3 were book/dissertation papers. Additionally, 101 studies (9.6%) aiming to establish clinical trial protocols and 37 studies (3.5%) involving secondary data analysis were not included. Fifteen additional studies (1.4%) published before 2002 and five studies (0.5%) where full text was unavailable were also excluded from the analysis. After excluding articles that were ineligible using these exclusion criteria, the research team reviewed the full texts and agreed on a final set of articles that fulfilled all predefined criteria (Fig. 1).

**Table 1:** Search area classification table

	Category 1	Category 2	Category 3
Key word	“rare disease” OR “rare diseases” in title/abstract	“survey” in title/abstract	“public” OR “patients” OR “family” OR “health professional” OR “researchers”
MesH	“rare disease” OR “rare diseases” in title/abstract OR “rare diseases” [MeSH]	“survey” in title/abstract OR “health care surveys” [MeSH]	“public” OR “patient” OR “patients” OR “family” OR “families” OR “health professional” OR “health professionals” OR “researcher” OR “researchers” OR “medical doctors” OR “medical doctor” OR “nurses” OR “nurse” OR “caregiver” OR “caregivers” OR “patients” [MeSH] OR “family” [MeSH] OR “health personnel” [MeSH] OR “physicians” [MeSH] OR “nurses” [MeSH] OR “research personnel” [MeSH] OR “caregivers” [MeSH]

### Data analysis

The selected articles were reviewed and extracted with data into EndNote X9.2. Each article was coded for author(s), publication year, type of disease(s), study locations, research aim(s), study design, data collection instruments, and major findings. The authors reviewed and resolved any conflicts by applying the exclusion criteria, where 34 full text studies and articles were excluded as they did not satisfy the exclusion criteria and conducted a thematic analysis of the study topics from the questionnaires used in the surveys to provide in-depth insights and identify any key themes or topics that emerged from the data. Across 115 articles, 36 provided full questionnaires, and the reviewers used NVivo 12 to extract the study topics.

## Results

### Overview and details of the selected research

Out of the 1,660 articles, 402 articles were identified through PubMed, 929 articles were from EMBASE, 88 articles were from Cochrane Central, 15 articles were from Web of Science, and 226 articles were from CINHAL. 115 articles satisfied the eligibility criteria and were selected for analysis in this review. The characteristics of the selected studies in terms of six specific domains are summarized in Table 2. These domains were selected for analysis as they were considered the most useful categories for answering the questions under investigation in this review as they relate to RD stakeholders’ by firstly, identifying stakeholders’ key characteristics including the nature of their stake i.e., their relationship to RD’s, the nature of the studies various focuses in terms of identifying specific stakeholder issues, which countries these studies are being conducted in, types of study and study focus over the review’s specified timescale.

**Table 2:** Characteristics of the 115 selected studies

Characteristics	Number of studies (%)	
Study participants		
Group 1 (patients, families, caregivers, and patient organizations)	77	(67.0)
Group 2 (specialists, GPs, and other healthcare professionals [HCPs])	18	(15.7)
Group 3 (others, including the public)	7	(6.1)
Mixed	13	(11.3)
Study locations		
Europe	46	(40.0)
North America	40	(34.8)
Asia	6	(5.2)
Oceania	7	(6.1)
South America	2	(1.7)
Mixed	14	(12.2)
Types of RD		
Unspecified	46	(40.0)
Endocrine, nutritional, or metabolic diseases (stem code #05)	20	(17.4)
Nervous system diseases (stem code #08)	11	(9.6)
Developmental anomalies (stem code #20)	10	(8.7)
Immune system diseases (stem code #04)	8	(7.0)
Diseases related to blood or blood-forming organs (stem code #03)	5	(4.3)
Mixed	5	(4.3)
Visual system diseases (stem code #09)	2	(1.7)
Circulatory system diseases (stem code #11)	2	(1.7)
Skin diseases (stem code #14)	2	(1.7)
Musculoskeletal system or connective tissue diseases (stem code #15)	2	(1.7)
Genitourinary system diseases (stem code #16)	1	(0.9)
Neoplasms (stem code #02)	1	(0.9)
Study topics		
Experience	74	(64.3)
Opinions	24	(20.9)
Knowledge	9	(7.8)
Prevalence	8	(7.0)
Study methodologies		
Quantitative	89	(77.4)
Qualitative	1	(0.9)
Mixed	25	(21.7)
Publication year		
2016–2020	83	(72.2)
2011–2015	25	(21.7)
2002–2010	7	(6.1)

### Trends in academic interest related to RD

**Fig. 2:**
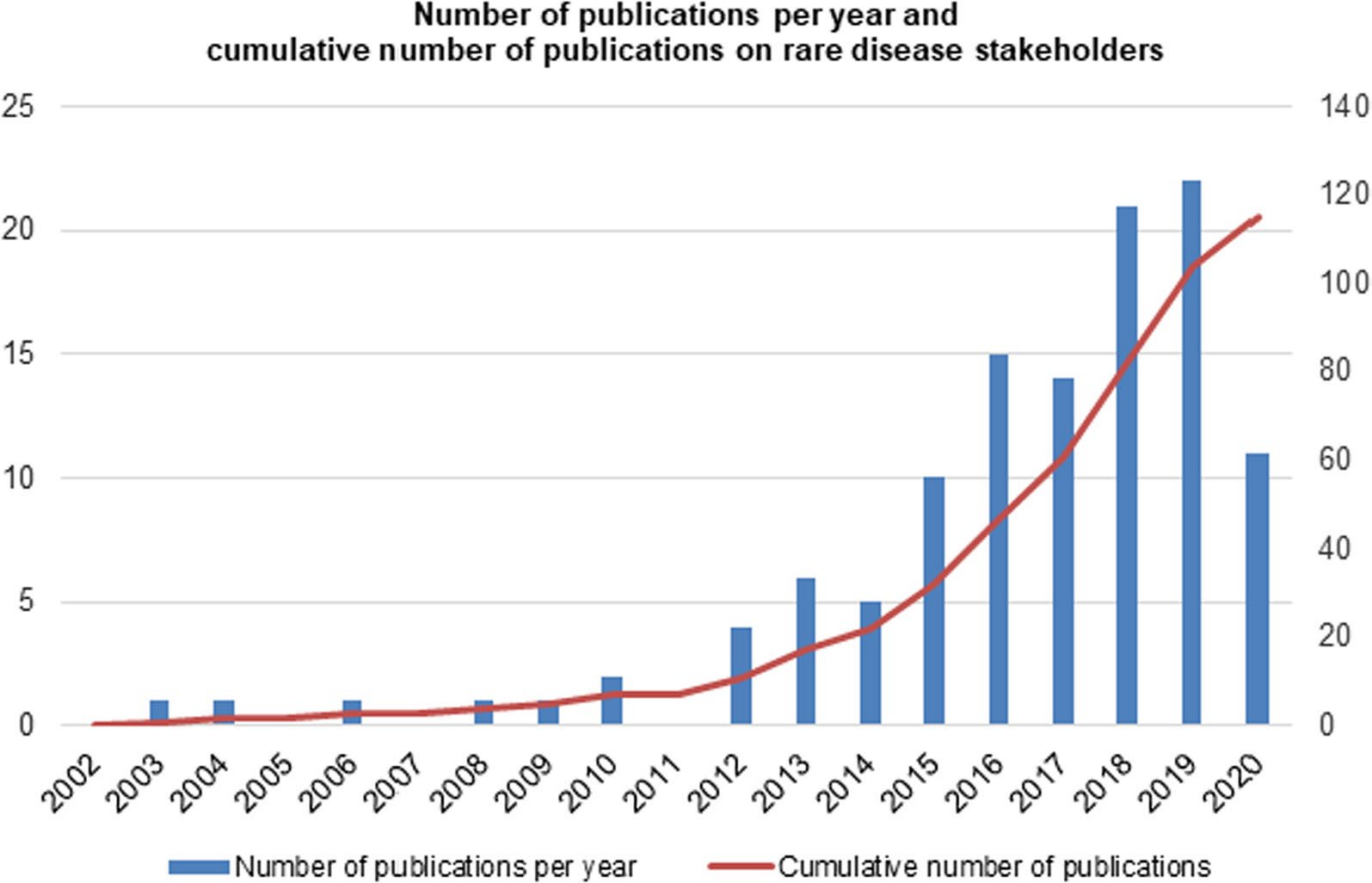
Publication year of the 115 selected studies

The number of studies on RD stakeholders has steadily increased over the years during the time period reviewed, with a notable, more rapid increase since 2015 (Fig. 2). In 2015, the number of studies doubled from the previous year, when only five studies were conducted. The cut-off date of the literature search in May 2020 and consequently, results indicate a lower number of publications in that year.

**Fig. 3:**
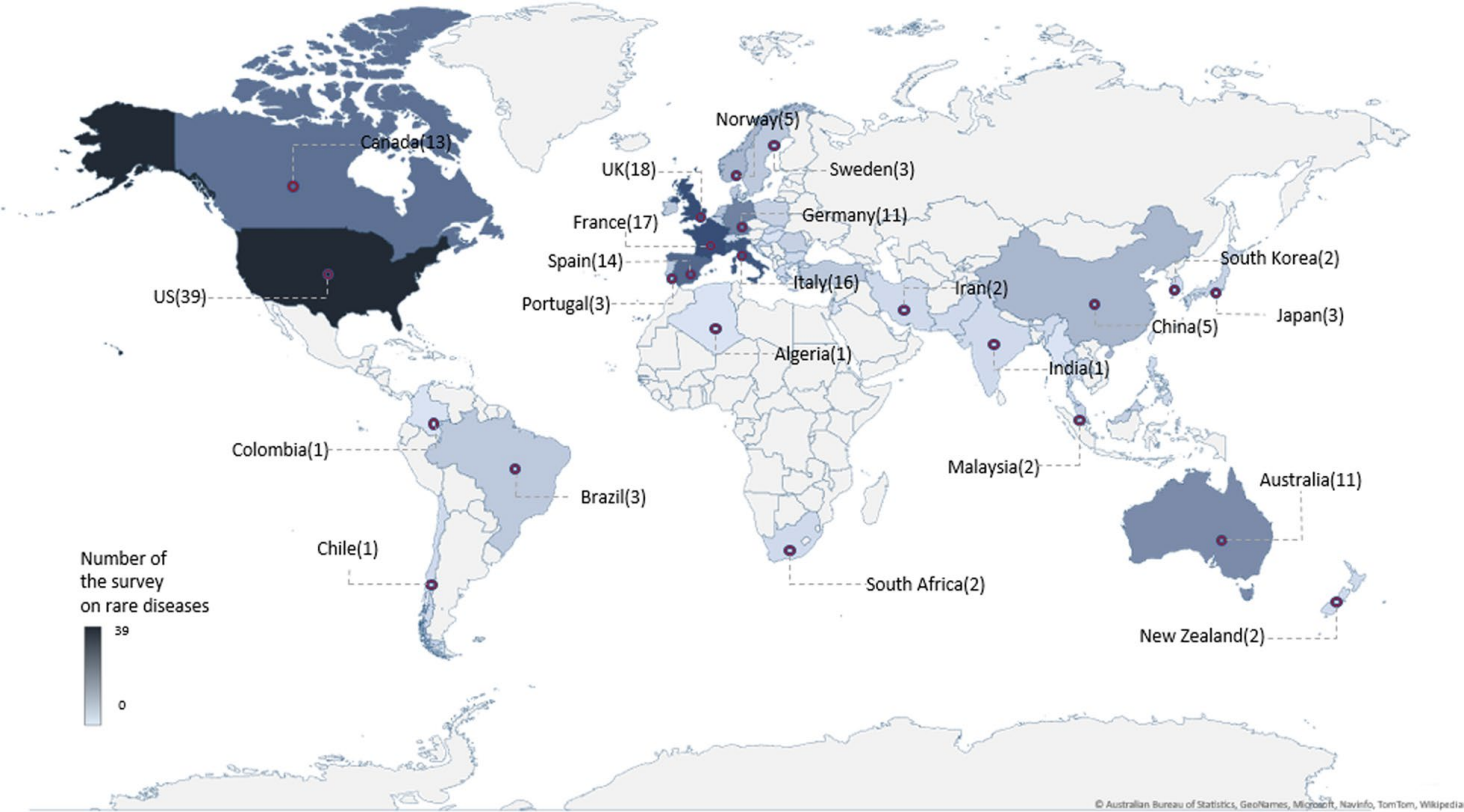
Study locations of the 115 selected studies

Additionally, it was observed that multiple jurisdictions have participated in international studies on RD. Study locations were categorized into six continents and analyzed based on the countries (Fig. 3, Additional file 1: Table S3). Across the 115 selected articles, Europe and North America were most commonly featured in RD research accounting for 93 articles (Table 1, Additional file 1: Table S3). Europe was selected as the exclusive study location in 45 articles, making it the region where the majority of the research in this area was conducted (39.1%). Fifty-two articles (45.2%) examined countries in North America where 13 (11.3%) focused on Canada and 39 (33.9%) focused on the US. Fifteen studies focused on the areas in Asia and Oceania, and only one study included both the regions. No single study was focused on Africa exclusively, but two studies included several countries in Africa (1.7%). The number of studies focused on two or more regions has increased over time, with 14 studies (12.2%) conducted since 2012, suggesting that international collaboration and joint research are becoming more common in areas of research related to RD. Europe has been the most involved in intercontinental studies, participating in 12 out of 14 studies (10.4%). North America was the second most involved continent, participating in 9 studies (10.3%). These two continents were the focus of 8 studies (7.0%). Europe and Asia collaborated in 4 articles (4.6%), and only 1 article featured South America (1.1%).

### Study participants

**Fig. 4:**
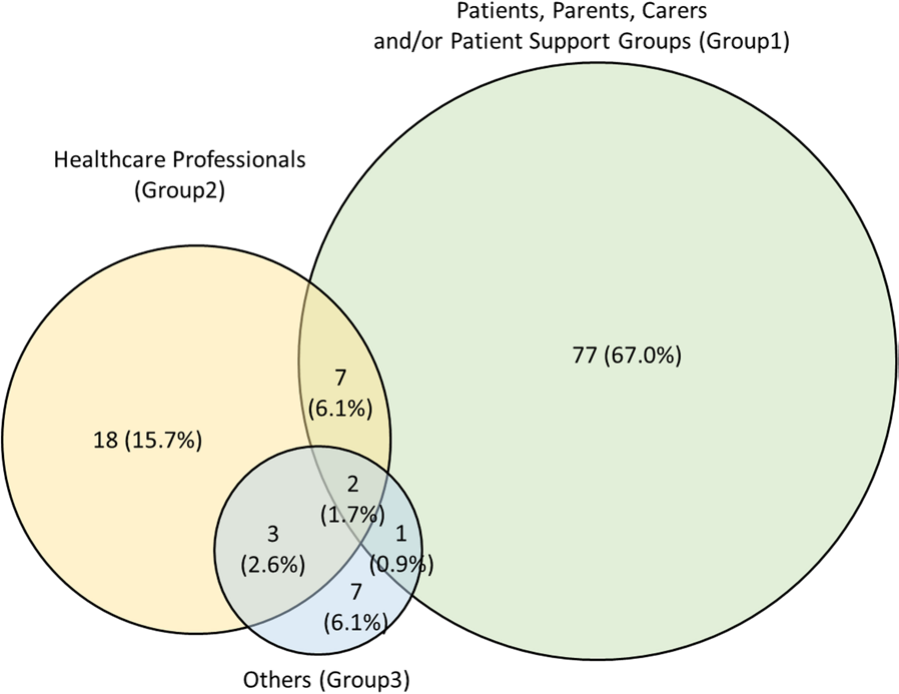
Stakeholders of the 115 selected articles

For this analysis, RD stakeholders were categorized into three groups and studies were categorized by the stakeholder participant (Fig. 4, Additional file 1: Table S4).

- Group 1: Patients, parents, caregivers, or patient organizations. This group was the most involved in RD research, with 87 articles (75.7%) including them as study participants compared with other RD stakeholders (Table 1).
  Patients and patient support groups: 41 studies (35.7%) included only patients or patient support groups.
  Families or caregivers: 18 studies (15.7%) included only families or caregivers.
  Both patients and families or caregivers: 18 studies (15.7%) included both patients and families or caregivers.
- Group 2: Healthcare professionals (HCPs). This group included specialists from specific medical disciplines, general practitioners (GPs), and other healthcare professionals (HCPs). 18 articles (20.7%) included HCPs as study participants.
  Specialists: Nine articles included specialists from specific medical disciplines, such as neurology, pediatrics, genetics, oncology, obstetrics, and pulmonology.
  GPs: Four articles included only GPs.
  Other HCPs: Two articles included nurses, dentists, or healthcare managers.
- Group 3: The public. This group included university students, social support practitioners, and research fund providers. Seven studies (8.1%) included only the public, while six studies included them along with patients, caregivers, or healthcare professionals.

### Types of RD

The types of RD in the 115 selected studies were analyzed in accordance with the International Classification of Diseases–11 Mortality and Morbidity Statistics (ICD-11 MMS). Unspecified RD were the most common among the selected articles in the review, accounting for 46 of the 115 articles (40.0%) (Table 1). Endocrine, nutritional, and metabolic diseases (stem code #05) were examined in 20 articles, being the most studied types of diseases with specific codes (17.4%). Neurological diseases (stem code #08) were exclusively examined in 11 articles (9.6%). Ten articles studied diseases labeled as developmental anomalies (stem code #20), including diseases such as Poland’s syndrome and Rett’s syndrome (8.7%). Autoimmune disorders (stem code #04) were studied in eight articles (7.0%). Five articles covered diseases related to blood or blood-forming organs, with four articles concentrating on hemophilia and one on thrombocytopenic purpura (4.3%). Two articles each focused on the visual system (stem code #09), circulatory system (stem code #11), skin (stem code #14), musculoskeletal system or connective tissue (stem code #15). Rare tumors (stem code #02) and genitourinary diseases (stem code #16) were investigated in only one study, respectively. The type of tumor included was not stated; however, nephrotic syndrome was the singular type of disease under the genitourinary disease category. Five articles investigated more than one disease, while endocrine, nutritional, and metabolic diseases were included in all of them (4.3%).

### Study topics

The majority of the studies included in the review were concerned with stakeholders’ experiences of RD (n = 74/115, 64.3%, Additional file 1: Table S4). A total of 24 studies examined the opinions of various stakeholders, with nine studies exploring knowledge level or education history (7.8%), and eight studies focusing on the prevalence of these diseases (7.0%). The remaining studies were focused on stakeholders’ feelings on diverse and various issues such as treatment preferences, access to information and funding (see Supplementary 4 for detailed breakdown of topic details).

Topics related to experiences with RD were selected as a research subject for more than half of Group 1 (n = 58/77, 75.3%) and Group 2 studies (n = 10/18, 55.6%), followed by the opinions of the stakeholders and their knowledge (n = 5/18, 27.8%, respectively). Questions concerning funding were most commonly surveyed in studies investigating Group 3 participants exclusively (n = 6/7, 85.7%). According to selected articles using questionnaires, four out of six of these studies inquired about the general public’s opinions on the use of governmental funding for RD (Additional file 1: Table S4).

Approximately 60% of the studies surveying participants from both Groups 1 and 2 investigated relevant stakeholders’ various opinions on patient engagement, diagnosis, treatment, or research priorities (n = 4/7, 57.1%), whereas the remaining studies investigated experiences of the diagnosis and treatment processes or QoL issues (n = 37/86, 42.9%). One article studied individuals from both Groups 1 and 3 surveyed their opinions on RD treatment, and two other studies involved participants from Groups 1, 2, and 3 and explored their experiences as far as the accessibility of information relevant to RD. Participants from Groups 2 and 3 were also asked about prevalence (n = 1/3, 33.3%) and their knowledge (n = 2/3, 66.7%) of RD.

In addition to this quantitative analysis, surveys from 36 articles were included in a thematic analysis for in-depth investigation. The number of times specific topics were included in the survey questionnaires was investigated. As questionnaires may cover many topics as well as the same topic multiple times, the total frequencies of each topic-category appearing in questionnaires will necessarily exceed the total number of articles reviewed.

**Fig. 5:**
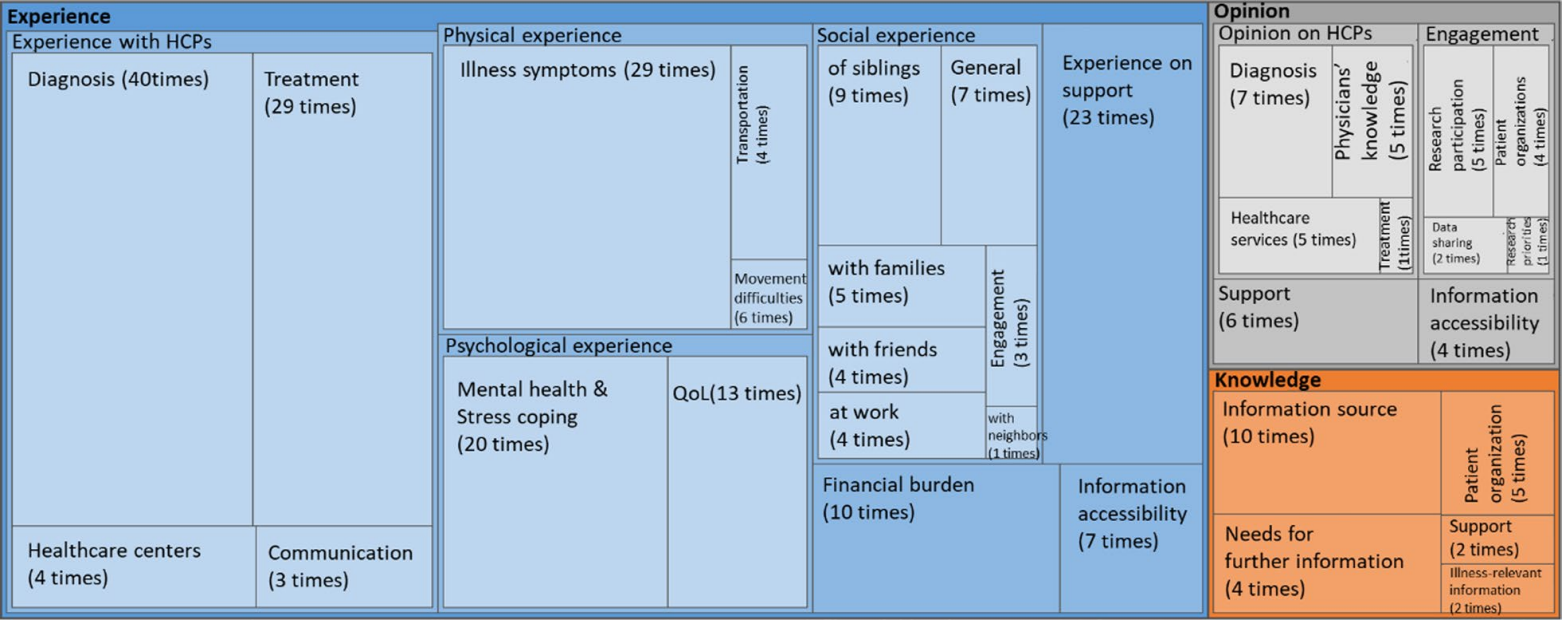
Thematic analysis of Group 1 studies (n = 23). The area of each box represents the details and frequency count of the questionnaires by categories of experience, opinions, and knowledge in the Group 1 studies

A total of 23 studies out of 36 included Group 1 participants as the study subjects (Fig. 5). The topic-category “experience” appeared in the survey questions a total of 230 times. Questions asking about the experience with the HCPs were mentioned 76 times, thereby accounting for the most frequent question topic among the 23 studies. Questions related to physical experiences (such as illness symptoms, transportation, or movement difficulties), psychological experiences (such as stress coping and QoL), and social experiences in various environments were asked 39, 33, and 33 times, respectively. Questions concerning the opinions of patients, families, or caregivers appeared 40 times. Survey participants who were living with RD were also asked their opinions on engagement 12 times. Their perspectives of participation in RD research were mentioned across five questions, their opinions on patient organizations appeared in four questions, and their perceptions of data sharing were queried two times.

**Fig. 6:**
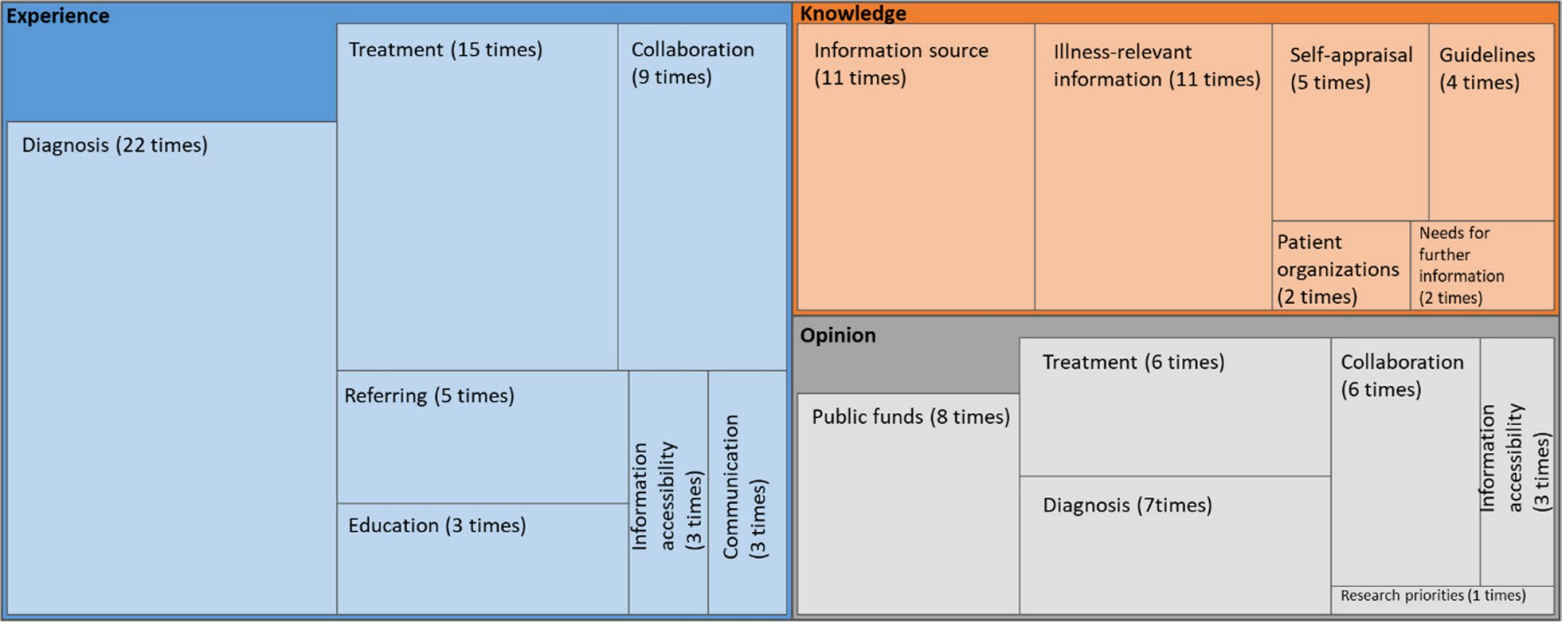
Thematic analysis of Group 2 studies (n = 12). The area of each box represents the details and frequency count of the questionnaires by categories of experience, opinions, and knowledge in the Group 2 studies

Questionnaires from 12 articles that surveyed Group 2 participants were also included in thematic analysis (Fig. 6). Questions about their experiences appeared 71 times, while the questions relating to their knowledge and opinions on RD were asked 35 and 34 times, respectively. Participants from Group 2 were asked to assess the level of their own knowledge of RD five times. Additionally, they were asked whether they were aware of the treatment and diagnosis guidelines four times.

**Fig. 7:**
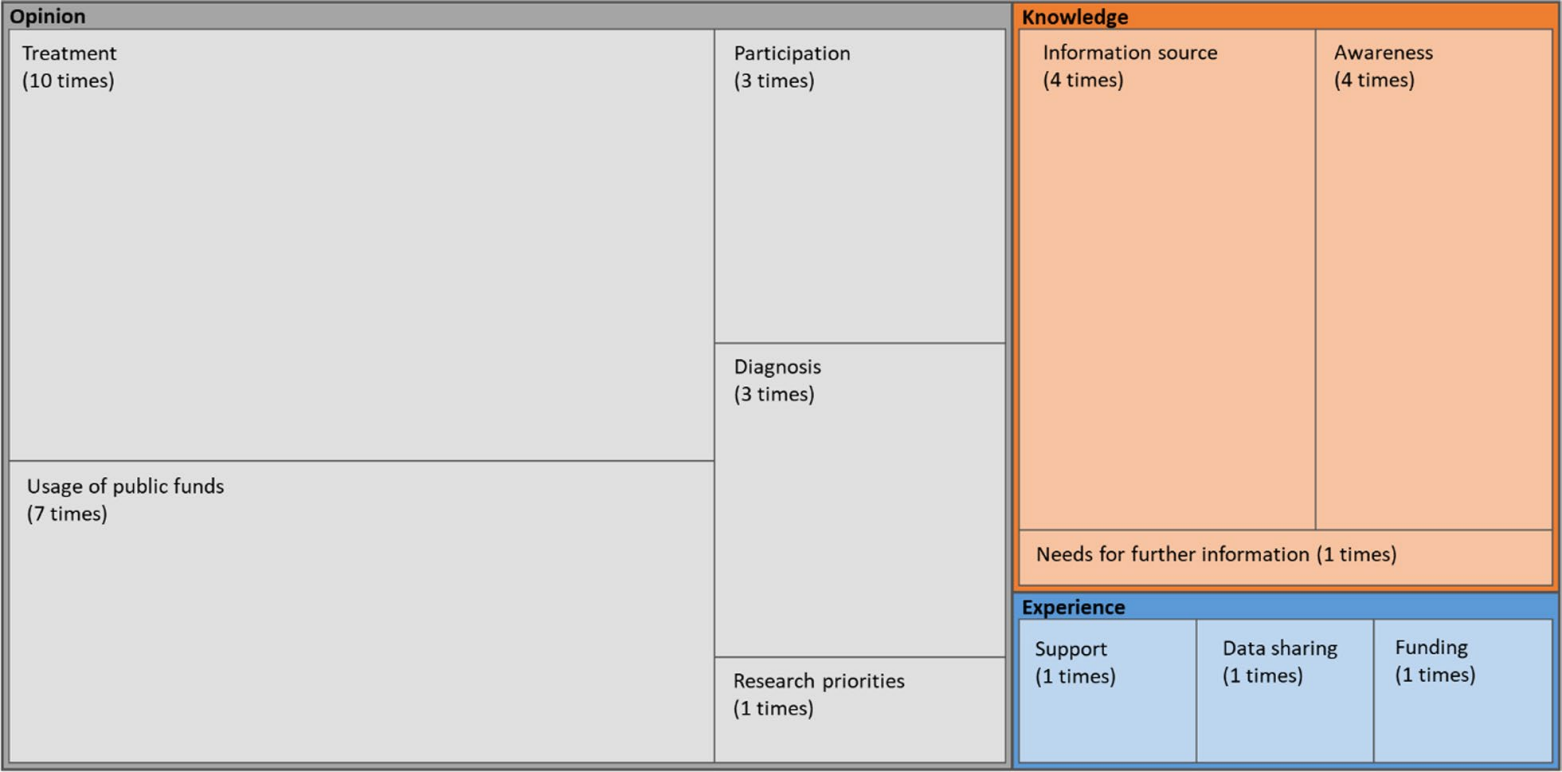
Thematic analysis of Group 3 studies (n = 5). The area of each box represents the details and frequency count of the questionnaires by categories of experience, opinions, and knowledge in the Group 3 studies

For the five Group 3 studies whose questionnaires could be accessed, public opinions were the most explored topic (Fig. 7). Opinions on the use of public funds for activity related to RD were asked seven times.

### Study methodologies

The dominant study methodology was the quantitative research method, which was used in 114 of the 115 selected articles (99.1%, Table 3, Additional file 1: Table S6). 89 studies adopted quantitative methods as exclusive methodologies in their studies (77.4%), while 80 articles used simple questionnaires to study various stakeholders, and nine articles selected diverse quantitative methodologies, such as Discrete Choice Experiments, 36-Item Short-Form Health Survey (SF-36), Acceptance of Illness Scale (AIS), Parental Needs Scale for RD (PNS-RD) (7.8%). On the other hand, 25 studies used both quantitative and qualitative methods for their research (21.7%), while questionnaire methodology was predominantly used as a joint method in 22 articles (19.1%). The interview research method was most aligned with questionnaires and was employed in 11 studies (9.6%). Literature review methodology was used in combination with questionnaires, discrete choice experiments, or the Delphi technique in eight studies (7.0%). Questionnaire methods were also sometimes combined with discussion methods (0.9%) and patient record analysis (0.9%), respectively. Only one study selected a qualitative research method—discussion—as the sole study method (0.9%).

**Table 3:** Study methodologies employed in the 115 selected articles

	Number of studies (%)	
Quantitative methodology	89	(77.4)
Questionnaire	80	(69.6)
Discrete choice experiments	4	(3.5)
36-Item Short-Form Health Survey (SF-36)	3	(2.6)
36-Item Short-Form Health Survey (SF-36) + Acceptance of Illness Scale (AIS)	1	(0.9)
Parental Needs Scale for RD (PNS-RD)	1	(0.9)
Mixed methodology	25	(20.9)
Interview + Questionnaire	11	(9.6)
Literature Review + Questionnaire	5	(4.3)
Open-ended Questionnaire	4	(3.5)
Literature Review + Discrete Choice Experiments	2	(1.7)
Literature Review + Delphi Technique	1	(0.9)
Discussion + Questionnaire	1	(0.9)
Patient Records Analysis + Questionnaire	1	(0.9)
Qualitative methodology	1	(0.9)
Discussion	1	(0.9)

## Discussion

### Importance of patient-centered studies

Medical studies, from clinical trials to health-related policy-making procedures, have rapidly become patient-centered, acknowledging patients, families, and caregivers as core stakeholders with first-hand experiences [7]. For example, studies on chronic diseases and cancers have shown a strong tendency to engage patients and caregivers with the aim of improving care and identifying the diseases [8]–9. Similar trends have been observed in RD sector as well. For instance, the EU supported the establishment of a patient alliance across member states—’European Organization for Rare Diseases (Eurordis)’—which has become one of the most important non-profit patient organizations in terms of bringing together all stakeholders. Since 1997, Eurordis has invited RD patients and caregivers to participate in a range of RD research related activity from surveys or discussion to various policy-making procedures. ‘EurordisCare’, a long-term survey engaged RD patients and caregivers over a period of seven years (2002–2008). This study has been fundamental to the alleviation of the burdens of illness experienced by RD stakeholders by investigating delays or inconvenient experiences in the diagnosis and treatment of RDs [10].

Another recent study also highlighted the need to prioritize the establishment of health policies to improve QoL for patients and develop patient-centered treatments [11]. It emphasized the fact that strong political and legal foundations are critical to addressing challenges related to RD, such as access to clinical information, and access to treatment and diagnosis. Future studies, similarly, should engage patients, families and caregivers and use survey methodologies to identify specific ways to improve the overall QoL and lower the burden of illness. RD patient-centered studies for improving the QoL of patients, families, and caregivers and alleviating the burden of cost associated with lifetime treatment should be the priority research areas for RD related studies.

### Estimating the knowledge of HCPs considering engagements with RD patients

The relationship between HCPs and RD patients or their caregivers is significantly different from the traditional patient-healthcare provider relationship. According to a study, RD patients and caregivers show a higher tendency to depend on and trust HCPs for diagnosis and treatment compared with patients who have non-rare diseases [12]. The professional opinions, perspectives and knowledge that HCPs acquire, become dominant factors in their interaction with patients and caregivers. This review indicates that RD patients lack effective access to relevant information and often experience late diagnosis and misdiagnosis, where there are generally limited options for treatment, despite the considerable benefits afforded by patient-centered studies [13]. Assessing whether the HCPs have the correct information and guidelines for treating and managing RD patients is extremely important. Opinions on their approaches to treatment, patient engagement and research priorities should also be sought from HCPs involved in the management of RDs. This will lead to an improved understanding of the realities faced by patients’ and caregivers’, their experiences and the role HCPs in the process.

### Importance of public studies for social support of RDs

This review found only seven studies seeking the opinions of the general public and their feelings on support for governmental funding in dealing with RDs. Studies involving the public are critical in encouraging democratic debates on policy-making procedures concerning the allocation of public-sector funds. This may help in discussions on priority setting and the allocation of public sector resources, especially for RD due to the low prevalence of these conditions among the population. For example, a study in the UK explored public understanding concerning the use of national funds for protecting RD patients and ensuring their access to orphan drugs [14]. The report indicated that 80% of the public respondents agreed with policies promoting the protection and wellbeing of patients with RD and their access to effective treatments, reaffirming public support for the use of government resources to ensure access to these treatments for RD patients. Although only seven of the selected articles examined public perceptions of RD, the use of public-sector funding was found to be the most common topic in both the systematic review and thematic analysis. Therefore, future studies on RD stakeholders should make efforts to include participants from the general public and determine their opinions on government investment in this area due to the importance of social support for RD patients.

### Increasing trends of national engagement and international cooperation

In addition to efforts to proactively enhance the rights of RD stakeholders in member states of the EU, by the Rare 2030 Foresight Study [15], Asian and Oceanian countries have also recently started to develop national plans and conduct studies with RD stakeholders. In 2015, Japan enacted the Act on Medical Care for Patients with Intractable Diseases and established the Japan Agency for Medical Research and Development plan. The First Initiative on Rare and Undiagnosed Diseases (IRUD) was inaugurated in the same year to identify potential undiagnosed and RD patients through genomic studies at the national level and establish platforms for data sharing [16]. Similarly, the Australian government formulated the National Strategic Action Plan for Rare Diseases in 2020 [17].

In addition to national policy developments, there has also been a marked increase in international partnerships in studies on RD stakeholders since 2010 [18]. EU member countries have experienced the benefits of RD data sharing through the portal for RD and orphan drugs, The Orphanet, and have gradually been able to inspire and facilitate regional cooperation. The study population of RD patients expands when countries collaborate and share data, which will hopefully aid in developing more effective treatment and diagnosis guidelines as well as improving general understanding of rare disease. Consequently, national cooperation and international exchanges related to rare diseases will facilitate more effective RD treatments and diagnosis by employing cross border data sharing initiatives [18].

### Limitations

This study has several limitations. Firstly, it should be noted that the review does not include the most recently published articles related to RD stakeholders, as data collection and analysis were completed in May 2020. In order to address this gap, contemporary initiatives have been considered to some extent, such as the rare 2030 foresight studies. Secondly, the selected articles lacked important and relevant information. For instance, out of the 115 articles analyzed, only 36 questionnaires were included, and among them, only 21 detailed topics related to experience, opinion, knowledge, and prevalence. Given the rapid expansion of RD diagnosis, treatment, and management options, it is important to publish the questionnaires used in RD survey research. By doing so, other researchers can utilize them to enhance and adapt their own investigations. Furthermore, sharing these surveys will aid in the identification of priority topics in RD, guiding public sector funding decisions and establishing a solid foundation for future research endeavors.

## Conclusion

Stakeholder research on RD reveals that there are significant unmet needs and challenges faced by the medical system in dealing with RDs. These stakeholders are motivated to enhance quality of life (QoL) for RD patients through prompt diagnosis and effective treatment. However, it is entirely possible that the research studies are not effectively reaching out to all relevant stakeholders. These studies also fail to identify a common set of priority areas related to RD that can be investigated further and analyzed which presents a considerable challenge for identifying policymaking and research prioritization related to RD. This suggests that studies need to adopt strategies for expanded stakeholder participation in order to increase RD awareness and indicates the importance of public sector funding in improving QoL for RD patients. Identifying and considering stakeholders from broader, more varied perspectives is crucial. Once they are effectively identified, it becomes possible to determine the relevant subjects or issues to include in research using RD stakeholder surveys.

Given the relatively small number of RD patients, it is essential that RD-related information, including research findings, is easily accessible. This will allow multi-country studies to consider the findings and the methodologies used to validate the efficacy and effectiveness of various approaches to diagnosis, treatment, and management. The number of cases within a country may be too small to draw statistically valid conclusions. This review demonstrates the increasing popularity of cross-national data sharing in RD research, emphasizing the need for enhanced robustness and reliability in the field. While small sample sizes may pose potential confidentiality challenges, there are also opportunities for the development of appropriate regulations that will encourage international data sharing—thereby benefiting RD treatment, medical drug or device development, as well as methods of diagnosis, treatment, management, and referral.

### Supplementary Information

**Additional file 1**. Supplementary material.

## Data Availability

Additional file.

## Abbreviations

AIS: Acceptance of illness scale
EU: European Union
HCPs: Healthcare professionals
ICD-11 MMS: International classification of diseases–11 mortality and morbidity statistics
IRUD: The initiative on rare and undiagnosed diseases
PNS-RD: Parental needs scale for RD
QoL: Quality of life
RD: Rare disease
SF-36: 36-Item short-form health survey
