# Supplementary material for: Prioritization of research engaged with rare disease stakeholders: a systematic review and thematic analysis
Source: Orphanet J Rare Dis. 2023 Nov 23;18:363. doi: 10.1186/s13023-023-02892-2 (PMC10668415; doi:10.1186/s13023-023-02892-2)
Supplement: Supplementary file 1 — Additional file 1. Supplementary material. [file 13023_2023_2892_MOESM1_ESM.docx]

**Supplementary material**

Supplementary table 1. Overview of 115 selected studies………………………………………………………… 2

Supplementary table 2. Exclusion criteria ………………………………………………………………………… 16

Supplementary table 3. Study locations …………………………………………………………………………… 17

Supplementary table 4. Stakeholders and study topics of 115 selected studies…………………………………… 20

Supplementary table 5. Types of diseases in accordance with ICD-11 MMS……………………………………… 24

Supplementary table 6. Study methodology ……………………………………………………………………… 28

Supplementary table 7. Specific topics of the 115 selected surveys by participant types ……………………… 31

Supplementary table 8. Excluded articles via screening (n=1,047)………………………………………………… 33

References…………………………………………………………………………………………………………… 87

**Supplementary table 1. Overview of 115 selected studies**

| **No.** | **Author(s) and Year** | **Source** | **Study participant(s)** | **Study location(s)** | **Type(s) of disease (ICD-11 MMS)** | **Study topic(s)** | **Methodology(ies)** | **T/A** |
| --- | --- | --- | --- | --- | --- | --- | --- | --- |
| 1 | Rudebeck et al (2020) [1] | JIMD Rep | Group 1 (Patients) | Asia (Unspecified) + Europe (France, Italy, Netherlands, Slovakia, Spain, UK) | Alkaptonuria (5C50.10) | Experience (diagnosis, treatment) | Quantitative (Questionnaire) | **o** |
| 2 | Mancuso et al (2020) [2] | Neurol Sci | Group 2 (Specialists (Neurologists)) | Europe (Italy) | Unspecified diseases of the nervous system (8E7Z) | Knowledge | Quantitative (Questionnaire) | **o** |
| 3 | Takeuchi et al (2020) [3] | Brain Dev | Group 2 (Specialists (Pediatrics, Neurology, Genetics)) | Asia (China, Hong Kong, India, Iran, Japan, Malaysia, Myanmar, Pakistan, Philippines, Singapore, South Korea, Taiwan, Thailand) + Oceania (Australia, New Zealand) | Duchenne muscular dystrophy (8C70.1) | Experience (treatment) | Quantitative (Questionnaire) | **o** |
| 4 | Yan, He and Dong (2020) [4] | Int J Environ Res Public Health | Group 1 (Patients) | Asia (China) | Unspecified | Experience (diagnosis) | Mixed (Questionnaire + Literature review) |  |
| 5 | Ramalle-Gómara et al (2020) [5] | Orphanet J Rare Dis | Group 2 (Specialists (Gynecology, Hematology, Neurology, Oncology, Pediatric), GPs) | Europe (Spain) | Unspecified | Education | Quantitative (Questionnaire) |  |
| 6 | Hartman et al (2020) [6] | Eur J Hum Genet | Group 3 (Funders) | North America (US) | Unspecified | Experience (ELSI funding, difficulties) | Mixed (Questionnaire + Discussion) | **o** |
| 7 | Requena-Fernández et al (2020) [7] | J Clin Med | Group 2 (Specialists (Pediatrics)) + Group 3 (Medical school students) | Europe (Spain) | α-1-Antitrypsin deficiency (5C5A), Primary ciliary dyskinesia (LA75.Y) | Knowledge | Quantitative (Questionnaire) | **o** |
| 8 | Painous et al (2020) [8] | Eur J Neurol | Group 2 (Specialists (Neurologists)) | Europe (unspecified) | Dystonia (MB47.4), Paroxysmal dyskinesia (8A02.2), Neurodegeneration with brain iron accumulation (5C64.1), Ataxias (MB45.0), Hereditary spastic paraparesis (8B44.0Z), Atypical parkinsonism 9(8A00.1Z), Choreas (10-8A0Y) | Experience (treatment) | Quantitative (Questionnaire) |  |
| 9 | Peeks et al (2020) [9] | J Inherit Metab Dis | Group 1 (Patients, Caregivers, Patient Organizations) + Group 2 (GPs, Other HCPs (Nurses, Dieticians)) | Africa + Asia + Europe + Oceania + South America + North America (unspecified) | Liver glycogen storage disease (5C51.3) | Opinion (research priorities) | Mixed (Questionnaire + Literature review) | **O (G1, G2)** |
| 10 | Byrne et al (2020) [10] | Ir J Med Sci | Group 2 (GPs) | Europe (Ireland) | Unspecified | Experience (access to information) | Quantitative (Questionnaire) |  |
| 11 | Quinn et al (2020) [11] | J Genet Couns | Group 1 (Patients, Families, Caregivers) | North America (US) | Unspecified | Experience (access to information) | Mixed (Questionnaire + Interview) | **o** |
| 12 | Qian et al (2019) [12] | J Community Genet | Group 1 (Patients, Families) | Asia (Malaysia) + North America (US) | Unspecified | Experience (assess to genetics-related information) | Quantitative (Questionnaire) | **o** |
| 13 | Marques-da-Silva et al (2019) [13] | JIMD Rep | Group 1 (Families) | Europe (unspecified) | Congenital disorder of glycosylation (5C54.0) | Experience (liver involvement) | Mixed (Questionnaire + Literature review) |  |
| 14 | Conner et al (2019) [14] | Orphanet J Rare Dis | Group 1 (Families) | North America (US) | Mucopolysaccharidosis Type I (5C56.30) | Experience (burden of illness, medical/educational needs after transplant) | Quantitative (Questionnaire) |  |
| 15 | Khair and Pelentsov (2019) [15] | Haemophilia | Group 1 (Families) | Europe (UK) | Haemophilia (3B12) | Knowledge | Quantitative (PNS-RD) |  |
| 16 | Noone et al (2019) [16] | Haemophilia | Group 1 (Patients) | Europe (Denmark, France, Germany, Netherlands, UK) | Haemophilia (3B12) | Experience (diagnosis, QoL) | Quantitative (Questionnaire) |  |
| 17 | Rush et al (2019) [17] | Orphanet J Rare Dis | Group 1 (Patients) | North America (US) | Hypophosphatasia (5C64.3) | Experience (burden of disease, QoL) | Mixed (Questionnaire + Interview) | **o** |
| 18 | Rice et al (2019) [18] | Disabil Rehabil | Group 1 (Caregivers) | Europe (France, UK) + North America (Canada, US) + Oceania (Australia) | Systemic sclerosis (4A42) | Experience (psychological distress, access of information) | Quantitative (Questionnaire) |  |
| 19 | Crowe et al (2019) [19] | Front Public Health | Group 1 (Patients, Families, Caregivers) +Group 2 (GPs, Other HCPs) + Group 3 (social support practitioners) | Europe (Northern Ireland) | Unspecified | Experience (source of knowledge, awareness) | Mixed (Questionnaire + Literature review) | **o** |
| 20 | Hollis et al (2019) [20] | Paediatr Child Heal | Group 1 (Families, Caregivers) | North America (Canada) | Nephrotic syndrome (GB41) | Experience (diagnosis, treatment) | Quantitative (Questionnaire) | **o** |
| 21 | Baldelli et al (2019) [21] | Orphanet J Rare Dis | Group 1 (Patients) | Europe (Italy) | Poland's syndrome (LB73.10) | Experience (diagnosis) | Quantitative (Questionnaire) |  |
| 22 | Yankouskaya et al (2019) [22] | J Clin Rheumatol | Group 1 (Patients) | Europe (UK) | Behcet's disease (4A62) | Opinion (illness perception, health status) | Quantitative (Questionnaire) |  |
| 23 | Swezey et al (2019) [23] | Osteoporos Int | Group 1 (Patients) | Asia (Unspecified) + Europe (Unspecified) + North America (US) + Oceania (Australia) | Osteogenesis imperfecta (LD24.K0) | Experience (symptoms, QoL) | Qualitative (Discussion) |  |
| 24 | Nakahara et al (2019) [24] | Disabil Rehabil | Group 1 (Patients) | Asia (Japan) | Fibrodysplasia ossificans progressiva (FB31.1) | Experience (QoL) | Quantitative (Questionnaire) |  |
| 25 | Morrison et al (2019) [25] | Orphanet J Rare Dis | Group 1 (Caregivers) | Asia (Turkey) + Europe (Germany, Netherlands, Spain) | Mucopolysaccharidosis type VII (5C56.3Y) | Experience (diagnosis, burden of illness) | Quantitative (Questionnaire) |  |
| 26 | Karaa et al (2019) [26] | Data Br | Group 1 (Patients, Caregivers) + Group 2 (Specialists (Mithochondrial)) | North America (US) | Unspecified | Experience (diagnosis, treatment) | Quantitative (Questionnaire) |  |
| 27 | Courbier et al (2019) [27] | Orphanet J Rare Dis | Group 1 (Patients, Families) | Europe (unspecified) | Unspecified | Opinion (data sharing) | Quantitative (Questionnaire) |  |
| 28 | Yoo et al (2019) [28] | J Allergy Clin Immunol | Group 1 (Caregivers) | North America (Canada, US) | Primary immunodeficiency disorders (4A00) | Experience (psychological distress) | Mixed (Open-ended questionnaire) | **o** |
| 29 | Marshall et al (2019) [29] | Genet Med | Group 1 (Families) | North America (Canada) | Unspecified | Opinion (diagnostic testing) | Quantitative (Discrete choice experiment) |  |
| 30 | Delisle et al (2019) [30] | Disabil Rehabil | Group 1 (Patients, Patients Organizations) | North America (Canada, US) | Scleroderma (EB61.Y) | Experience (Self-efficacy) | Quantitative (Questionnaire) |  |
| 31 | López-Bastida et al (2019) [31] | Health Policy | Group 2 (Other HCPs: healthcare managers, clinical/health economists) | Europe (England, France, Germany, Italy, Spain) | Unspecified | Opinion (societal value) | Mixed (Discrete choice experiment + Literature review) |  |
| 32 | López-Bastida et al (2019) [32] | Orphanet J Rare Dis | Group 1 (Patients) | Europe (Italy) | Cystic fibrosis (CA25), Haemophilia (3B12) | Opinion (societal value) | Mixed (Discrete choice experiment + Literature review) |  |
| 33 | Baqué et al (2019) [33] | Press Medicale | Group 2 (GPs) | Europe (France) | Unspecified | Experience (diagnosis, treatment) | Mixed (Questionnaire + Interview) |  |
| 34 | Ivarsson et al (2018) [34] | Clin Respir J | Group 1 (Patients) | Europe (Sweden) | Primary pulmonary hypertension (BB01.0) | Experience (medication adherence, access to information) | Quantitative (Questionnaire) |  |
| 35 | Fu et al (2018) [35] | Ann Allergy, Asthma Immunol | Group 2 (GPs) | North America (Canada) | Hereditary angioedema (4A00.14) | Experience (treatment) | Quantitative (Questionnaire) |  |
| 36 | Peiris et al (2018) [36] | J Med Devices, Trans ASME | Group 2 (Specialists (Pediatrics, Neurology, Pulmonology, Cardiology)) | North America (US) | Unspecified | Experience (treatment) | Quantitative (Questionnaire) |  |
| 37 | Ho et al (2018) [37] | J Pediatr | Group 1 (Patients, Caregivers) | North America (US) | Epilepsy (8A6Z) | Prevalence (comorbidities) | Quantitative (Questionnaire) |  |
| 38 | Tory et al (2018) [38] | Pediatr Rheumatol | Group 1 (Patients, Families) + Group 2 (Specialists (Rheumatologists), Nurses) | North America (US) | Juvenile idiopathic inflammatory myositis (4A41.01) | Opinion (QoL) | Quantitative (Questionnaire) |  |
| 39 | Schadewald et al (2018) [39] | JIMD Rep | Group 1 (Caregivers) | North America (US) | Mucopolysaccharidosis (5C56.3) | Experience (coping) | Mixed (Open-ended questionnaire) |  |
| 40 | Efthymiadou et al (2018) [40] | Int. J. Technol. Assess. Health Care | Group 1 (Patients) | Europe (France, Romania, UK) + North America (US) | Unspecified | Experience (QoL) | Quantitative (Questionnaire) |  |
| 41 | Lopes et al (2018) [41] | Clinics (Sao Paulo, Brazil) | Group 1 (Patients, Families, Patient Organizations) + Group 2 (Specialists, GPs) | South America (Brazil) | Unspecified | Opinion (diagnosis, treatment) | Mixed (Questionnaire + Interview) |  |
| 42 | Kanavin and Fjermestad (2018) [42] | Orphanet J Rare Dis | Group 1 (Patients) | Europe (Norway) | Hereditary spastic paraplegia (8B44.0) | Prevalence (gastrointestinal and urinary complaints) | Quantitative (Questionnaire) |  |
| 43 | McCausland et al (2018) [43] | Patient | Group 1 (Patients) + Group 2 (Specialists (Nephrologists, Hematologists)) | North America (US) | Light chain amyloidosis (5D00.0) | Experience (diagnosis) | Mixed (Questionnaire + Interview) |  |
| 44 | Fu et al (2018) [44] | J Chinese Pharm Sci | Group 1 (Patients) | Asia (China) | Unspecified | Experience (financial burden of disease, access 45to medical ser46vice) | Quantitative (Questionnaire) |  |
| 45 | Liu et al (2018) [45] | J Investig Med | Group 1 (Patients) | North America (US) | Acromegaly (5A60.0) | Expe47rience (burden48 of diseases, Q49oL, treat50ment) | Quantitative (Questionnaire) |  |
| 46 | Chung et al (2018) [46] | Urology | Group 1 (Patients) | North America (US) | Paget's disease (FB85.Z) | Experience (diagnosis, treatment) | Quantitative (Questionnaire) |  |
| 47 | Peyron et al (2018) [47] | Social Science and Medicine | Group 1 (Families) | Europe (France) | Unspecified | Opinion (treatment) | Quantitative (Discrete choice experiment) |  |
| 48 | Richardson et al (2018) [48] | J Genet Couns | Group 1 (Patients, Families) | Oceania (Australia) | Catecholaminergic polymorphic ventricular tachycardia (BC65.5) | Experience (psychosocial outcomes, QoL) | Mixed (Open-ended questionnaire) | **o** |
| 49 | Kazimierska-Zając et al (2018) [49] | J Neurol Neurosurg Nurs | Group 1 (Patients) | Europe (Poland) | Spinocerebellar ataxia (8A03.16) | Experience (QoL) | Quantitative (SF-36 + AIS) |  |
| 50 | Cockerell et al (2018) [50] | BMC Nephrol | Group 1 (Patients, Families) | Europe (France, Norway) | Tuberous sclerosis (LD2D.2) | Knowledge | Quantitative (Questionnaire) |  |
| 51 | Adachi et al (2018) [51] | Orphanet J Rare Dis | Group 2 (GPs) + Group 3 (University hospitals account executives) | Asia (Japan) | Unspecified | Prevalence | Quantitative (Questionnaire) | **o** |
| 52 | Ivleva et al (2018) [52] | JIMD Rep | Group 1 (Patients) | Europe (UK) | Fabry’s(-Anderson) disease (5C56.01) | Experience (QoL) | Quantitative (Questionnaire) |  |
| 53 | Szczepura et al (2018) [53] | Clin Genet | Group 1 (Families) | Europe (UK) | Unspecified | Experience (diagnosis, care, counselling) | Quantitative (Questionnaire) |  |
| 54 | Xun et al (2018) [54] | J Commun Healthc | Group 1 (Patients) | North America (US) | α-1-Antitrypsin deficiency (5C5A) | Opinion (access to information) | Mixed (Open-ended questionnaire) |  |
| 55 | Zurynski et al (2017) [55] | Orphanet J Rare Dis | Group 1 (Patients, Families) | Oceania (Australia) | Unspecified | Experience (diagnosis) | Quantitative (Questionnaire) | **o** |
| 56 | Richardson et al (2017) [56] | PharmacoEconomics - Open | Group 3 (Public) | Oceania (Australia) | Unspecified | Opinion (funds) | Mixed (Questionnaire + Interview) |  |
| 57 | Chen et al (2017) [57] | Value Health | Group 1 (Patients) | North America (US) | Haemophilia B (3B11.0) | Experience (treatment, symptoms) | Quantitative (Questionnaire) |  |
| 58 | Polisena et al (2017) [58] | BMC Health Serv. Res | Group 3 (Public) | North America (Canada) | Unspecified | Opinion (funds) | Quantitative (Questionnaire) |  |
| 59 | Bogart and Irvin (2017) [59] | Orphanet J Rare Dis | Group 1 (Patients) | North America (US) | Unspecified | Experience (QoL) | Quantitative (Questionnaire) |  |
| 60 | Patriquin et al (2017) [60] | J Clin Apher | Group 2 (Specialists (Nephrologists, Hematologists)) | North America (Canada) | Thrombotic thrombocytopenic purpura (3B64.14) | Experience (treatment) | Quantitative (Questionnaire) | **o** |
| 61 | Amelot et al (2017) [61] | Pharmaceut Med | Group 1 (Patients, Families) | Europe (France) | Friedreich’s ataxia (8A03.10) | Opinion (expectation concerning the announcement of results for the clinical trial, scientific publication, willingness) | Quantitative (Questionnaire) | **o** |
| 62 | Wiss et al (2017) [62] | Med Decis Mak | Group 3 (Public) | Europe (Sweden) | Unspecified | Opinion (treatments) | Quantitative (Questionnaire) |  |
| 63 | Zurynski et al (2017) [63] | BMJ Paediatr Open | Group 2 (Specialists (Pediatricians)) | Oceania (Australia) | Unspecified | Experience (treatment, access to information) | Quantitative (Questionnaire) | **o** |
| 64 | Schwartz et al (2017) [64] | Qual Life Res | Group 1 (Patients) | North America (US) | Unspecified | Experience (QoL, resilience) | Quantitative (Questionnaire) | **o** |
| 65 | Lloyd et al (2017) [65] | Curr Med Res Opin | Group 3 (Public) | Europe (UK) | Fabry’s(-Anderson) disease (5C56.01) | Opinion (treatments) | Quantitative (Discrete choice experiment) | **o** |
| 66 | Bayliss et al (2017) [66] | Orphanet J Rare Dis | Group 1 (Patients) | North America (US) | Light chain amlyoidosis (5D00.0) | Experience (QoL, burden of illness) | Quantitative (SF-36) |  |
| 67 | O’Hara et al (2017) [67] | Orphanet J Rare Dis | Group 1 (Patients) + Group 2 (Specialists (Haematologists)) | Europe (France, Germany, Italy, Spain, UK) | Haemophilia (3B12) | Experience (burden of illness) | Quantitative (Questionnaire) |  |
| 68 | Heuyer et al (2017) [68] | Patient Relat Outcome Meas | Group 1 (Patients, Families) | Europe (France) | Unspecified | Experience (diagnosis, communications) | Quantitative (Questionnaire) |  |
| 69 | Cacioppo et al (2016) [69] | Am J Med Genet Part C Semin Med Genet | Group 1 (Families) | North America (US) | Cornelia de Lange syndrome (LD2F.1Y) | Experience (with internet support group) | Mixed (Questionnaire + Interview) |  |
| 70 | Engelstad et al (2016) [70] | Hum Reprod | Group 1 (Patients, Families) + Group 3 (Donors) | North America (US) | Unspecified | Opinion (treatment) | Quantitative (Questionnaire) |  |
| 71 | Xin et al (2016) [71] | Chin Med J (Engl) | Group 1 (Patients) | Asia (China) | Unspecified | Experience (financial burden) | Quantitative (Questionnaire) |  |
| 72 | Behan et al (2016) [72] | Eur Respir J | Group 1 (Patients, Families) | Africa (Algeria, South Africa) + Asia (Iran, Qatar) + Europe (Austria, Belgium, Cyprus, Denmark, France, Germany, Greece, Ireland, Italy, Norway, Poland, Portugal, Spain, Switzerland, UK) + North America (Canada, US) + Oceania (Australia) + South America (Chile) | Primary ciliary dyskinesia (LA75.Y) | Experience (diagnosis, treatment) | Mixed (Questionnaire + Interview) | **o** |
| 73 | Karaa et al (2016) [73] | Mol Genet Metab | Group 1 (Patients, Families) | North America (US) | Unspecified | Experience (treatment) | Quantitative (Questionnaire) |  |
| 74 | Serrano-Aguilar et al (2016) [74] | Expert Opin. Orphan Drugs | Group 1 (Patients) | Europe (Spain) | Unspecified hereditary retinal dystrophy (9B70) | Experience (health problems, access to information, treatment) | Mixed (Delphi technique + Literature review) | **o** |
| 75 | Darquy et al (2016) [75] | Eur J Hum Genet | Group 1 (Patients, Families) | Europe (Belgium, France, Germany, Italy, Spain) | Leukodystrophies (8A44) | Opinion (data sharing) | Quantitative (Questionnaire) |  |
| 76 | Morel et al (2016) [76] | Orphanet J Rare Dis | Group 1 (Patients, Caregivers) | Europe (UK) | Unspecified | Opinion (preference in treatment) | Quantitative (Discrete choice experiment) | **o** |
| 77 | Delisle et al (2016) [77] | Clin Exp Rheumatol | Group 1 (Patients) | North America (Canada) | Scleroderma (EB61.Y) | Opinion (engagement) | Quantitative (Questionnaire) |  |
| 78 | Péntek et al (2016) [78] | Eur J Heal Econ | Group 1 (Patients, Caregivers) | Europe (Bulgaria, France, Germany, Hungary, Italy, Spain, Sweden) | Mucopolysaccharidosis (5C56.3) | Experience (QoL) | Quantitative (Questionnaire) |  |
| 79 | Senger et al (2016) [79] | Appl Nurs Res | Group 1 (Families) | North America (US) | Unspecified | Experience (stress, coping) | Quantitative (Questionnaire) |  |
| 80 | Molster et al (2016) [80] | Orphanet J Rare Dis | Group 1 (Patients) | Oceania (Australia) | Unspecified | Experience (diagnosis, access to information, support service) | Quantitative (Questionnaire) |  |
| 81 | Merkel et al (2016) [81] | Orphanet J Rare Dis | Group 1 (Patient organizations) | North America (US) | Unspecified | Opinion (engagement, network) | Quantitative (Questionnaire) |  |
| 82 | Carpenter et al (2016) [82] | Curr Rheumatol Rev | Group 1 (Patients) | North America (US) | Vasculitis (4A44), Lupus (4A40.Z) | Experience (support, constraint) | Quantitative (Questionnaire) | **o** |
| 83 | Pelentsov et al (2016) [83] | BMC Fam. Pract | Group 1 (Families) | Oceania (Australia, New Zealand) | Unspecified | Experience (satisfaction with care, needs of providing daily care) | Quantitative (Questionnaire) |  |
| 84 | Bosch et al (2015) [84] | Orphanet J Rare Dis | Group 1 (Patients, Families) | Asia (Turkey) + Europe (France, Germany, Italy, Netherlands, Spain, UK) | Phenylketonuria (5C50.0) | Experience (QoL) | Quantitative (Questionnaire) |  |
| 85 | Dragojlovic et al (2015) [85] | Patient | Group 3 (Public) | North America (Canada) | Unspecified | Opinion (funds) | Quantitative (Questionnaire) | **o** |
| 86 | Ramalle-Gõmara et al (2015) [86] | J Eval Clin Pract | Group 2 (GPs) + Group 3 (University students, vocational health education students, nursing students) | Europe (Spain) | Unspecified | Knowledge | Quantitative (Questionnaire) | **o** |
| 87 | Melo et al (2015) [87] | J Community Genet | Group 2 (GPs, Other HCPs (Nurses, Dentists)) | South America (Brazil) | Unspecified | Knowledge | Quantitative (Questionnaire) |  |
| 88 | Tosi et al (2015) [88] | Orphanet J Rare Dis | Group 1 (Patients) | North America (US) | Osteogenesis imperfecta (LD24.K0) | Experience (epidemiological features) | Quantitative (Questionnaire) | **o** |
| 89 | Oliveira et al (2015) [89] | Orphanet J Rare Dis | Group 1 (Patients, Caregivers) | Europe (Portugal) | Unspecified | Experience (innovative solutions) | Mixed (Questionnaire + Interview) |  |
| 90 | Shin et al (2015) [90] | Cancer Res Treat | Group 2 (Specialists (Oncologists)) | Asia (South Korea) | Rare cancer diseases (No code, Neoplasms) | Experience (treatment) | Quantitative (Questionnaire) | **o** |
| 91 | Samoutis and Samoutis (2015) [91] | Int J Caring Sci | Group 2 (Specialists (Orthopedics, Rheumatologists)) | Europe (Cyprus) | Paget's disease (FB85.Z) | Prevalence | Quantitative (Questionnaire) |  |
| 92 | Forsythe et al (2015) [92] | J Comp Eff Res | Group 1 (Patients) + Group 2 (GPs) | North America (US) | Unspecified | Opinion (comparative effectiveness research, engagement) | Quantitative (Questionnaire) |  |
| 93 | Johnson et al (2015) [93] | Muscle Nerve | Group 1 (Patients) | North America (US) | Charcot-Marie-tooth disease (8C21.Z) | Prevalence (frequency, location, severity, duration) | Quantitative (Questionnaire) | **o** |
| 94 | Houyez et al (2014) [94] | Interact J Med Res | Group 1 (Patients, Families) + Group 2 (Other HCPs) + Group 3 (Public) | Europe (Bulgaria, Croatia, Denmark, France, Italy, Portugal, Romania, Spain) | Unspecified | Experience (access to information) | Quantitative (Questionnaire) | **o** |
| 95 | McClain et al (2014) [95] | Clin. Pediatr. (Phila) | Group 2 (GPs) | North America (US) | Unspecified | Experience (care) | Quantitative (Questionnaire) |  |
| 96 | Hendriksz et al (2014) [96] | Orphanet J. Rare Dis | Group 1 (Patients, Caregivers) | Asia (Turkey) + Europe (Germany, Spain, UK) +South America (Brazil, Columbia) | Morquio syndrome (5C56.32) | Experience (burden of illness, QoL) | Quantitative (Questionnaire) |  |
| 97 | Smorti et al (2014) [97] | Psychol Heal Med | Group 1 (Patients) | Europe (Italy) | Light chain amyloidosis (5D00.0) | Experience (depression, cardiac symptoms) | Quantitative (Questionnaire) |  |
| 98 | Johnson et al (2014) [98] | Neuromuscul Disord | Group 1 (Patients) | Europe (Italy, UK) + Oceania (Australia) + North America (US) | Charcot-Marie-tooth disease (8C21.Z) | Experience (symptoms, QoL) | Mixed (Questionnaire + Interview) |  |
| 99 | Greulich et al (2013) [99] | Respir Med | Group 2 (Specialists (Pulmonologists, Internal medicine specialists), GPs) | Europe (Germany, Italy) | α-1-Antitrypsin deficiency (5C5A) | Knowledge | Mixed (Questionnaire + Patient records analysis) | **o** |
| 100 | Zhou et al (2013) [100] | Ophthalmic Physiol Opt | Group 1 (Patients, Carriers) | Europe (UK) + North America (Canada, US) | Choroideremia (9B61) | Prevalence | Quantitative (Questionnaire) | **o** |
| 101 | Anderson et al (2013) [101] | Orphanet J. Rare Dis | Group 1 (Families, Caregivers) | Oceania (Australia) | Lysosomal storage disease (5C56.Z), Unspecified | Experience (diagnosis, healthcare service use) | Quantitative (Questionnaire) | **o** |
| 102 | Calvert et al (2013) [102] | Qual Life Res | Group 1 (Patients) | Oceania (Australia) | Charcot-Marie-tooth disease (8C21.Z) + Huntington’s disease (8A01.10) + Motor neuron disease (8B60.Z) + Multiple system atrophy (8D87.0Z) + Post Polio syndrome (8B62) + Progressive supranuclear palsy (8A00.10) + Ataxia (MB45.0) | Experience (QoL) | Quantitative (Questionnaire) |  |
| 103 | Desser (2013) [103] | Soc Sci Med | Group 2 (Specialists, GPs) | Oceania (Australia) | Unspecified | Opinion (treatment) | Quantitative (Questionnaire) | **o** |
| 104 | Tozzi et al (2013) [104] | Orphanet J. Rare Dis | Group 1 (Families) | Europe (Italy) | Unspecified | Experience (internet use- access to information, online communities) | Quantitative (Questionnaire) |  |
| 105 | Motil et al (2012) [105] | J Pediatr Gastroenterol Nutr | Group 1 (Families) | North America (Canada, US) | Rett’s syndrome (LD90.4) | Prevalence (gastrointestinal and nutritional disorders) | Quantitative (Questionnaire) | **o** |
| 106 | Carpenter et al (2012) [106] | J Am Pharm Assoc | Group 1 (Patients) | Europe (Unspecified) + Oceania (Australia) + North America (Canada, US) | Vasculitis (4A44) | Experience (source of knowledge) | Quantitative (Questionnaire) |  |
| 107 | Henschke (2012) [107] | Health Policy (New York) | Group 1 (Patients) | Europe (Germany) | Duchenne muscular dystrophy (8C70.1), Amyotrophic lateral sclerosis (8B60.5) | Experience (financial burden, financial supports/regulations) | Mixed (Questionnaire + Interview) |  |
| 108 | Zhao et al (2012) [108] | Intractable Rare Dis Res | Group 2 (Specialists (Maternal and Child Health)) | Asia (China) | Polyostotic fibrous dysplasia (FB80.0), Marfan’s syndrome (LD28.0), Haemophilia (3B12), Osteogenesis imperfecta (LD24.K0), Classical phenylketonuria (5C50.00), Neurofibromatosis (LD2D.1Z) | Knowledge | Mixed (Questionnaire + Literature review) |  |
| 109 | Wicks et al (2010) [109] | Intractable Rare Dis Res | Group 1 (Patients) | North America (US) | Unspecified | Experience (access to information) | Quantitative (Questionnaire) | **o** |
| 110 | Desser et al (2010) [110] | BMJ (Online) | Group 3 (Public) | Europe (Norway) | Unspecified | Opinion (funds) | Quantitative (Questionnaire) |  |
| 111 | Imrie et al (2009) [111] | J Med Econ | Group 1 (Patients, Families, Caregivers) | Europe (UK) | Niemann-Pick disease type C (5C56.0Y) | Experience (economic burden, healthcare use) | Quantitative (Questionnaire) |  |
| 112 | Abularrage et al (2008) [112] | J Vasc Surg | Group 1 (Patients) | North America (US) | Takayasu's arteritis (4A44.1) | Experience (QoL) | Quantitative (SF-36) |  |
| 113 | Galéra (2006) [113] | Am J Med Genet | Group 1 (Patients) | Europe | Costello syndrome (LD2F.1Y) | Prevalence (Behavioral aspects, temperamental features) | Quantitative (Questionnaire) |  |
| 114 | Pasculli et al (2004) [114] | Qual Life Res | Group 1 (Patients) | Europe (Italy) | Hereditary haemorrhagic telangiectasia (LA90.00), Rendu-Osler-Weber disease (LA90.00) | Experience (QoL) | Quantitative (SF-36) |  |
| 115 | Robey et al (2003) [115] | Dev Med Child Neurol | Group 1 (Families) | North America (US) | Lesch-Nyhan syndrome (5C55.01) | Experience (psychological distress, self-mutilation) | Quantitative (Questionnaire) |  |

*In determining the order of numbers of the 115 seleted studies, we used the publichation year as a criterion (by descending order). The article number is aligned with the following tables (Supplementary table 3-7). T/A: Thematic Analysis using NVivo

**Supplementary table 2. Exclusion criteria**

| **No.** | **Exclusion criteria** | | **Number of studies (%)** | |
| --- | --- | --- | --- | --- |
| 1 | Non-English surveys | | 6 | (0.6) |
| 2 | Non-rare disease surveys | | 106 | (10.1) |
| 3 | Non-human stakeholder surveys | | 55 | (5.3) |
| 4 | Non-Peer Reviewed Journal | | 722 | (69.0) |
|  | 4-1 | Conference/Meeting abstract | (456) |  |
|  | 4-2 | Comment/Review/Editorial/Discussion/Letter | (66) |  |
|  | 4-3 | Case Report | (72) |  |
|  | 4-4 | Clinical Paper | (125) |  |
|  | 4-5 | Book/Dissertation | (3) |  |
| 5 | Surveys aiming to establish clinical trial protocols | | 101 | (9.6) |
| 6 | Secondary data analysis studies | | 37 | (3.5) |
| 7 | Published before 2002 | | 15 | (1.4) |
| 8 | Full text unavailable | | 5 | (0.5) |
| **Total** | | | **1,047** | (100.0) |

**Supplementary table 3. Study locations**

| **Article #** | **Continent** | | **Countries** |
| --- | --- | --- | --- |
| 9* | Africa+Asia+Europe+Oceania+North America+South America | C1+C2+C3+C4+C5+C6 | Canada, US |
| 72 | Africa+Asia+Europe+Oceania+North America+South America | C1+C2+C3+C4+C5+C6 | Belgium, Denmark, France, Germany, UK, US, Italy, Australia, Switzerland, Canada, Cyprus, Ireland, Greece, South Africa, Austria, Chile, Portugal, Qatar, Poland, Iran, Norway, Spain, Algeria |
| 4 | Asia | C3 | China |
| 24 | Asia | C3 | Japan |
| 44 | Asia | C3 | China |
| 51 | Asia | C3 | Japan |
| 71 | Asia | C3 | China |
| 90 | Asia | C3 | South Korea |
| 108 | Asia | C3 | China |
| 1 | Asia+Europe | C1+C3 | France, Italy, Netherlands, Slovakia, Spain, UK |
| 25 | Asia+Europe | C1+C3 | Germany, Netherlands, Spain, Turkey |
| 84 | Asia+Europe | C1+C3 | France, Germany, Italy, Netherlands, Spain, Turkey, UK |
| 23* | Asia+Europe+Oceania+North America | C1+C2+C3+ C4 | Australia, US |
| 96 | Asia+Europe+South America | C1+C3+C5 | Brazil, Colombia, Germany, Spain, Turkey, UK |
| 12 | Asia+North America | C2+C3 | Malaysia, US |
| 3 | Asia+Oceania | C3+C4 | Australia, China, Hong Kong, India, Iran, Japan, Malaysia, Myanmar, New Zealand, Pakistan, Philippines, Singapore, South Korea, Taiwan, Thailand |
| 2 | Europe | C1 | Italy |
| 5 | Europe | C1 | Spain |
| 7 | Europe | C1 | Spain |
| 8* | Europe | C1 |  |
| 10 | Europe | C1 | Ireland |
| 13* | Europe | C1 |  |
| 15 | Europe | C1 | UK |
| 16 | Europe | C1 | Denmark, France, Germany, Netherlands, UK |
| 19 | Europe | C1 | Norther Ireland |
| 21 | Europe | C1 | Italy |
| 22 | Europe | C1 | UK |
| 27* | Europe | C1 |  |
| 31 | Europe | C1 | England, France, Germany, Italy, Spain |
| 32 | Europe | C1 | Italy |
| 33 | Europe | C1 | France |
| 34 | Europe | C1 | Sweden |
| 42 | Europe | C1 | Norway |
| 47 | Europe | C1 | France |
| 49 | Europe | C1 | Poland |
| 50 | Europe | C1 | France, Norway |
| 52 | Europe | C1 | UK |
| 53 | Europe | C1 | UK |
| 61 | Europe | C1 | France |
| 62 | Europe | C1 | Sweden |
| 65 | Europe | C1 | UK |
| 67 | Europe | C1 | France, Germany, Italy, Spain, UK |
| 68 | Europe | C1 | France |
| 74 | Europe | C1 | Spain |
| 75 | Europe | C1 | Belgium, France, Germany, Italy, Spain |
| 76 | Europe | C1 | UK |
| 78 | Europe | C1 | Bulgaria, France, Germany, Hungary, Italy, Spain, Sweden |
| 86 | Europe | C1 | Spain |
| 89 | Europe | C1 | Portugal |
| 91 | Europe | C1 | Cyprus |
| 94 | Europe | C1 | Bulgaria, Croatia, Denmark, France, Italy, Portugal, Romania, Spain |
| 97 | Europe | C1 | Italy |
| 99 | Europe | C1 | Germany, Italy |
| 102 | Europe | C1 | UK |
| 103 | Europe | C1 | Norway |
| 104 | Europe | C1 | Italy |
| 107 | Europe | C1 | Germany |
| 110 | Europe | C1 | Norway |
| 111 | Europe | C1 | UK |
| 113 | Europe | C1 | France |
| 114 | Europe | C1 | Italy |
| 40 | Europe+North America | C1+C2 | France, Romania, UK, US |
| 100 | Europe+North America | C1+C2 | UK, Canada, US |
| 18 | Europe+Oceania+North America | C1+C2+C4 | Australia, Canada, France, UK, US |
| 98 | Europe+Oceania+North America | C1+C2+C4 | Australia, Italy, UK, USA |
| 106* | Europe+Oceania+North America | C1+C2+C4 | US, Cadana, Europe, Australia |
| 6 | North America | C2 | US |
| 11 | North America | C2 | US |
| 14 | North America | C2 | US |
| 17 | North America | C2 | US |
| 20 | North America | C2 | Canada |
| 26 | North America | C2 | US |
| 28 | North America | C2 | Canada, US |
| 29 | North America | C2 | Canada |
| 30 | North America | C2 | Canada, US |
| 35 | North America | C2 | Canada |
| 36 | North America | C2 | US |
| 37 | North America | C2 | US |
| 38 | North America | C2 | US |
| 39 | North America | C2 | US |
| 43 | North America | C2 | US |
| 45 | North America | C2 | US |
| 46 | North America | C2 | US |
| 54 | North America | C2 | US |
| 57 | North America | C2 | US |
| 58 | North America | C2 | Canada |
| 59 | North America | C2 | US |
| 60 | North America | C2 | Canada |
| 64 | North America | C2 | US |
| 66 | North America | C2 | US |
| 69 | North America | C2 | US |
| 70 | North America | C2 | US |
| 73 | North America | C2 | US |
| 77 | North America | C2 | Canada |
| 79 | North America | C2 | US |
| 81 | North America | C2 | US |
| 82 | North America | C2 | US |
| 85 | North America | C2 | Canada |
| 88 | North America | C2 | US |
| 92 | North America | C2 | US |
| 93 | North America | C2 | US |
| 95 | North America | C2 | US |
| 105 | North America | C2 | Canada, US |
| 109 | North America | C2 | US |
| 112 | North America | C2 | US |
| 115 | North America | C2 | US |
| 48 | Oceania | C4 | Australia |
| 55 | Oceania | C4 | Australia |
| 56 | Oceania | C4 | Australia |
| 63 | Oceania | C4 | Australia |
| 80 | Oceania | C4 | Australia |
| 83 | Oceania | C4 | Australia, New Zealand |
| 101 | Oceania | C4 | Australia |
| 41 | South America | C5 | Brazil |
| 87 | South America | C5 | Brazil |

*Article 8, 9, 13, 23, 27, and 106 did not identify specific countries. C1 is Europe, C2 is North America, C3 is Asia, C4 is Oceania, C5 is South America, C6 is Africa.

**Supplementary table 4. Stakeholders and study topics of 115 selected studies**

| **Article #** | **Subject group** | **Subject** | **Key topic** | **Topic details** |
| --- | --- | --- | --- | --- |
| 18 | 1 | Caregivers | Experience |  |
| 25 | 1 | Caregivers | Experience | Diagnosis, burden of illness |
| 28 | 1 | Caregivers | Experience | Psychological distress |
| 39 | 1 | Caregivers | Experience | Coping |
| 13 | 1 | Families | Experience | Liver involvement |
| 14 | 1 | Families | Experience | Burden of illness, medical/educational needs after transplant |
| 15 | 1 | Families | Knowledge |  |
| 29 | 1 | Families | Opinion | Diagnostic testing |
| 47 | 1 | Families | Opinion | Treatment |
| 69 | 1 | Families | Experience |  |
| 79 | 1 | Families | Experience | Stress, coping |
| 83 | 1 | Families | Experience | Satisfaction with care, needs of providing daily care |
| 104 | 1 | Families | Experience | Internet use- access to information, online communities |
| 105 | 1 | Families | Prevalence | Gastrointestinal and nutritional disorders |
| 115 | 1 | Families | Experience | Self-mutilation |
| 53 | 1 | Families | Experience | Diagnosis, care, counselling |
| 20 | 1 | Families, Caregivers | Experience | Diagnosis, treatment |
| 101 | 1 | Families, Caregivers | Experience | Diagnosis, healthcare service use |
| 81 | 1 | Patient support groups | Opinion | Engagement, network |
| 1 | 1 | Patients | Experience | Diagnosis, treatment |
| 4 | 1 | Patients | Experience | Diagnosis |
| 16 | 1 | Patients | Experience | Diagnosis, QoL |
| 21 | 1 | Patients | Experience | Diagnosis |
| 22 | 1 | Patients | Opinion | Illness perception, health status |
| 23 | 1 | Patients | Experience |  |
| 24 | 1 | Patients | Experience | QoL |
| 32 | 1 | Patients | Opinion | Societal value |
| 34 | 1 | Patients | Experience | Medication adherence, access to information |
| 40 | 1 | Patients | Experience | QoL |
| 42 | 1 | Patients | Prevalence | Gastrointestinal and urinary complaints |
| 44 | 1 | Patients | Experience | Ginancial burden of disease, access to medical service |
| 45 | 1 | Patients | Experience | Burden of diseases, QoL, treatment |
| 46 | 1 | Patients | Experience | Diagnosis, treatment |
| 52 | 1 | Patients | Experience | QoL |
| 54 | 1 | Patients | Opinion | Access to information |
| 57 | 1 | Patients | Experience | Treatment, symptoms |
| 59 | 1 | Patients | Experience | QoL |
| 64 | 1 | Patients | Experience | QoL, resilience |
| 66 | 1 | Patients | Experience | QoL, burden of illness |
| 71 | 1 | Patients | Experience | Financial burden |
| 74 | 1 | Patients | Experience | Health problems, access to information, treatment |
| 77 | 1 | Patients | Experience |  |
| 80 | 1 | Patients | Experience | Diagnosis, access to information, support service |
| 82 | 1 | Patients | Experience | Support, constraint |
| 88 | 1 | Patients | Experience | Epidemiological features |
| 93 | 1 | Patients | Prevalence | Grequency, location, severity, duration |
| 97 | 1 | Patients | Experience | Depression, cardiac symptoms |
| 98 | 1 | Patients | Experience | Symptoms, QoL |
| 102 | 1 | Patients | Experience | QoL |
| 106 | 1 | Patients | Experience | Source of knowledge |
| 107 | 1 | Patients | Experience | Financial burden, financial supports/regulations |
| 109 | 1 | Patients | Experience | Access to information |
| 112 | 1 | Patients | Experience | QoL |
| 113 | 1 | Patients | Prevalence | Behavioral aspects, temperamental features |
| 114 | 1 | Patients | Experience | QoL |
| 17 | 1 | Patients | Experience | Burden of disease, QoL |
| 49 | 1 | Patients | Experience | QoL |
| 37 | 1 | Patients, Caregivers | Prevalence | Comorbidities |
| 76 | 1 | Patients, Caregivers | Opinion | Preference in treatment |
| 78 | 1 | Patients, Caregivers | Experience | QoL |
| 89 | 1 | Patients, Caregivers | Experience | Innovative solutions |
| 96 | 1 | Patients, Caregivers | Experience | Burden of illness, QoL |
| 30 | 1 | Patients, Patients Support Group | Self-efficacy |  |
| 100 | 1 | Patients, Carriers | Prevalence |  |
| 12 | 1 | Patients, Families | Experience | Assess to genetics-related information |
| 27 | 1 | Patients, Families | Opinion | Data sharing |
| 48 | 1 | Patients, Families | Experience | Psychosocial outcomes, QoL |
| 50 | 1 | Patients, Families | Knowledge |  |
| 55 | 1 | Patients, Families | Experience | Diagnosis |
| 61 | 1 | Patients, Families | Opinion | Expectation concerning the announcement of results for the clinical trial, scientific publication, willingness |
| 68 | 1 | Patients, Families | Experience | Diagnosis, communications |
| 72 | 1 | Patients, Families | Experience | Diagnosis, treatment |
| 73 | 1 | Patients, Families | Experience | Treatment |
| 75 | 1 | Patients, Families | Opinion | Data sharing |
| 84 | 1 | Patients, Families | Experience | QoL |
| 11 | 1 | Patients, Families, Caregivers | Experience | Access to information |
| 111 | 1 | Patients, Families, Caregivers | Experience | Economic burden, healthcare use |
| 99 | 2 | GP, Specialists | Knowledge | . |
| 103 | 2 | GP, Unidentified specialists | Opinion | Treatment |
| 31 | 2 | Other HCPs | Opinion | Preferences |
| 10 | 2 | Physicians | Experience | . |
| 33 | 2 | Physicians | Experience | . |
| 35 | 2 | Physicians | Experience | Treatment |
| 95 | 2 | Physicians | Experience | . |
| 87 | 2 | Physicians, Other HCPs | Knowledge | . |
| 5 | 2 | Physicians, Specialists | Education | . |
| 2 | 2 | Specialists | Knowledge | . |
| 3 | 2 | Specialists | Experience | Treatment |
| 8 | 2 | Specialists | Opinion | . |
| 36 | 2 | Specialists | Experience | . |
| 60 | 2 | Specialists | Experience | Treatment |
| 63 | 2 | Specialists | Experience | Treatment, access to information |
| 90 | 2 | Specialists | Experience | Treatment |
| 91 | 2 | Specialists | Prevalence | . |
| 108 | 2 | Specialists | Knowledge | . |
| 56 | 3 | Public | Opinion | Funds |
| 58 | 3 | Public | Opinion | Funds |
| 62 | 3 | Public | Opinion | Preference |
| 65 | 3 | Public | Opinion | Treatments |
| 85 | 3 | Public | Opinion | Funds |
| 110 | 3 | Public | Opinion | Funds |
| 6 | 3 | Others | Experience | ELSI funding, difficulties |
| 86 | Mixed (2+3) | Physicians, Others | Knowledge | . |
| 70 | Mixed (1+3) | Others, Patients, Families | Opinion | Treatment |
| 9 | Mixed (1+2) | Patients, Caregivers, Other HCPs, Physicians, Patient Support Organizations | Opinion | Research priorities |
| 26 | Mixed (1+2) | Patients, Caregivers, Specialists | Experience | Diagnosis, treatment |
| 19 | Mixed (1+2+3) | Patients, Families, Carers, HCP, medical doctors, social support practitioners etc | Experience | Psychological, access to informatin, communication needs) |
| 38 | Mixed (1+2) | Patients, Families, Nurses, Specialists (rheumatologists) | Opinion | . |
| 41 | Mixed (1+2) | Patients, Families, Support groups, NGOs, Physicians, Specialists (unidentified) | Opinion | Diagnosis, treatment |
| 94 | Mixed (1+2+3) | Patients, HCP, Families, Students | Experience | . |
| 92 | Mixed (1+2) | Patients, Physicians | Opinion | Comparative effectiveness research, engagement |
| 43 | Mixed (1+2) | Patients, Specialists | Experience | Diagnosis |
| 51 | Mixed (2+3) | Physicians, University hospitals | Prevalence | . |
| 7 | Mixed (2+3) | Specialists, Medical school students | Knowledge | . |
| 67 | Mixed (1+2) | Specialists, Patients | Experience | Burden of illness |

**Supplementary table** **5. Types of diseases of 115 selected studies, in accordance with ICD-11 MMS**

| **Article #** | **Disease name** | **Disease type** | **ICD 11** |
| --- | --- | --- | --- |
| 90 | - | 02. Neoplasms | General (tumor, no code) |
| 15 | Haemophilia | 03. Disease of the blood or blood-forming organs | 3B12 |
| 16 | Haemophilia | 03. Disease of the blood or blood-forming organs | 3B12 |
| 57 | Haemophilia B | 03. Disease of the blood or blood-forming organs | 3B11.0 |
| 60 | Thrombotic thrombocytopenic purpura | 03. Disease of the blood or blood-forming organs | 3B64.14 |
| 67 | Haemophilia | 03. Disease of the blood or blood-forming organs | 3B12 |
| 18 | Systemic Sclerosis | 04. Diseases of the immune system | 4A42 |
| 22 | Behcet's disease | 04. Diseases of the immune system | 4A62 |
| 28 | Primary Immunodeficiency Disorders | 04. Diseases of the immune system | 4A00 |
| 35 | Hereditary angioedema | 04. Diseases of the immune system | 4A00.14 |
| 38 | Juvenile Idiopathic Inflammatory Myositis | 04. Diseases of the immune system | 4A41.01 |
| 82 | Vasculitis Lupus | 04. Diseases of the immune system | 4A40.Z + 4A44 |
| 106 | Vasculitis | 04. Diseases of the immune system | 4A44 |
| 112 | Takayasu's Arteritis | 04. Diseases of the immune system | 4A44.1 |
| 1 | Alkaptonuria | 05. Endocrine, nutritional or metabolic diseases | 5C50.10 |
| 9 | Liver Glycogen Storage Disease | 05. Endocrine, nutritional or metabolic diseases | 5C51.3 |
| 13 | Congenital disorder of glycosylation | 05. Endocrine, nutritional or metabolic diseases | 5C54.0 |
| 14 | Mucopolysaccharidosis Type I | 05. Endocrine, nutritional or metabolic diseases | 5C56.30 |
| 17 | Hypophosphatasia | 05. Endocrine, nutritional or metabolic diseases | 5C64.3 |
| 25 | Mucopolysaccharidosis Type VII | 05. Endocrine, nutritional or metabolic diseases | 5C56.3Y |
| 39 | Mucopolysaccharidosis | 05. Endocrine, nutritional or metabolic diseases | 5C56.3 |
| 43 | Light Chain Amyloidosis | 05. Endocrine, nutritional or metabolic diseases | 5D00.0 |
| 45 | Acromegaly | 05. Endocrine, nutritional or metabolic diseases | 5A60.0 |
| 52 | Fabry’s(-Anderson) disease | 05. Endocrine, nutritional or metabolic diseases | 5C56.01 |
| 54 | α-1-Antitrypsin deficiency | 05. Endocrine, nutritional or metabolic diseases | 5C5A |
| 65 | Fabry’s(-Anderson) disease | 05. Endocrine, nutritional or metabolic diseases | 5C56.01 |
| 66 | Light Chain Amlyoidosis | 05. Endocrine, nutritional or metabolic diseases | 5D00.0 |
| 78 | Mucopolysaccharidosis | 05. Endocrine, nutritional or metabolic diseases | 5C56.3 |
| 84 | Phenylketonuria | 05. Endocrine, nutritional or metabolic diseases | 5C50.0 |
| 96 | Morquio syndrome | 05. Endocrine, nutritional or metabolic diseases | 5C56.32 |
| 97 | Light Chain Amyloidosis | 05. Endocrine, nutritional or metabolic diseases | 5D00.0 |
| 99 | α-1-Antitrypsin deficiency | 05. Endocrine, nutritional or metabolic diseases | 5C5A |
| 111 | Niemann-Pick disease type C | 05. Endocrine, nutritional or metabolic diseases | 5C56.0Y |
| 115 | Lesch-Nyhan syndrome | 05. Endocrine, nutritional or metabolic diseases | 5C55.01 |
| 32 | Cystic Fibrosis (endocrine)  Haemophilia (vascular) | 05. Endocrine, nutritional or metabolic diseases + 03. Disease of the blood or blood-forming organs | CA25 + 3B12 |
| 7 | α-1-Antitrypsin deficiency, Primary ciliary dyskinesia | 05. Endocrine, nutritional or metabolic diseases + 20. Developmental anomalies | 5C5A + LA75.Y |
| 101 | lysosomal storage disease (endocrine), mitochondrial disease (neurological) | 05. Endocrine, nutritional or metabolic diseases + General | 5C56.Z + General (mitochondrial disorders) |
| 8 | Dystonia, Paroxysmal dyskinesia, neurodegeneration with brain iron accumulation, ataxias, hereditary spastic paraparesis, atypical parkinsonism, choreas | 08. Disease of the nervous system + 05. Endocrine, nutritional or metabolic diseases | MB47.4 + 8A02.2 + 5C64.1 + MB45.0 + 8B44.0Z + 8A00.1Z + 8A0Y |
| 2 | - | 08. Diseases of the nervous system | 8E7Z |
| 3 | Duchenne muscular dystrophy | 08. Diseases of the nervous system | 8C70.1 |
| 37 | Epilepsy | 08. Diseases of the nervous system | 8A6Z |
| 42 | Hereditary spastic paraplegia | 08. Diseases of the nervous system | 8B44.0 |
| 49 | Spinocerebellar Ataxia | 08. Diseases of the nervous system | 8A03.16 |
| 61 | Friedreich’s ataxia(autosomal recessive) | 08. Diseases of the nervous system | 8A03.10 |
| 75 | Leukodystrophies | 08. Diseases of the nervous system | 8A44 |
| 93 | Charcot-Marie-Tooth disease | 08. Diseases of the nervous system | 8C21.Z |
| 98 | Charcot-Marie-Tooth disease | 08. Diseases of the nervous system | 8C21.Z |
| 102 | Charcot-Marie-Tooth disease, Huntington's Disease, Motor Neuron Disease, Multiple System Atrophy, Postpolio Syndrome, Progressive Supranuclear Palsy, Ataxia | 08. Diseases of the nervous system | 8C21.Z + 8A01.10 + 8B60.Z + 8D87.0Z + 8B62 + 8A00.10 + MB45.0 |
| 107 | Duchenne muscular dystrophy, Amyotrophic Lateral Sclerosis | 08. Diseases of the nervous system | 8C70.1 + 8B60.5 |
| 74 | Unspecified hereditary retinal dystrophy | 09. Diseases of the visual system | 9B70 |
| 100 | Choroideremia | 09. Diseases of the visual system | 9B61 |
| 34 | Primary pulmonary hypertension | 11. Disease of the circulatory system | BB01.0 |
| 48 | Catecholaminergic polymorphic ventricular tachycardia | 11. Disease of the circulatory system | BC65.5 |
| 30 | Scleroderma | 14. Diseases of the skin | EB61.Y |
| 77 | Scleroderma | 14. Diseases of the skin | EB61.Y |
| 46 | Paget's disease | 15. Diseases of the musculoskeletal system or connective tissue | FB85.Z |
| 91 | Paget's disease | 15. Diseases of the musculoskeletal system or connective tissue | FB85.Z |
| 108 | Polyostotic fibrous dysplasia, Marfan’s syndrome, Haemophilia,  Osteogenesisimperfecta  Classicalphenylketonuria,  Neurofibromatosis | 15. Diseases of the musculoskeletal system or connective tissue + 20. Developmental anomalies + 03. Diseases of the blood or blood-forming organs + 05. Endocrine, nutritional or metabolic diseases | FB80.0 + LD28.0 + 3B12 + LD24.K0 + 5C50.00 + LD2D.1Z |
| 20 | Nephrotic Syndrome | 16. Disease of the genitourinary system | GB41 |
| 21 | Poland's Syndrome | 20. Developmental anomalies | LB73.10 |
| 23 | Osteogenesis imperfecta | 20. Developmental anomalies | LD24.K0 |
| 24 | Fibrodysplasia ossificans progressiva | 20. Developmental anomalies | FB31.1 |
| 50 | Tuberous sclerosis | 20. Developmental anomalies | LD2D.2 |
| 69 | Cornelia de Lange Syndrome | 20. Developmental anomalies | LD2F.1Y |
| 72 | Primary ciliary dyskinesia | 20. Developmental anomalies | LA75.Y |
| 88 | Osteogenesis imperfecta | 20. Developmental anomalies | LD24.K0 |
| 105 | Rett’s syndrome | 20. Developmental anomalies | LD90.4 |
| 113 | Costello syndrome | 20. Developmental anomalies | LD2F.1Y |
| 114 | Hereditary haemorrhagic telangiectasia Rendu-Osler-Weber disease | 20. Developmental anomalies | LA90.00 |
| 4 | - | General | General |
| 5 | - | General | General |
| 6 | - | General | General |
| 10 | - | General | General |
| 11 | - | General | General |
| 12 | - | General | General |
| 19 | - | General | General |
| 26 | - | General | General (mitochondrial disorders) |
| 27 | - | General | General |
| 29 | - | General | General |
| 31 | - | General | General |
| 33 | - | General | General |
| 36 | - | General | General |
| 40 | - | General | General |
| 41 | - | General | General |
| 44 | - | General | General |
| 47 | - | General | General |
| 51 | - | General | General |
| 53 | - | General | General (Rare Chromosome disorder) |
| 55 | - | General | General |
| 56 | - | General | General |
| 58 | - | General | General |
| 59 | - | General | General |
| 62 | - | General | General |
| 63 | - | General | General |
| 64 | - | General | General |
| 68 | - | General | General |
| 70 | - | General | General (mitochondrial disorders) |
| 71 | - | General | General |
| 73 | - | General | General (mitochondrial disorders) |
| 76 | - | General | General |
| 79 | - | General | General (mitochondrial disorders) |
| 80 | - | General | General |
| 81 | - | General | General |
| 83 | - | General | General |
| 85 | - | General | General |
| 86 | - | General | General |
| 87 | - | General | General |
| 89 | - | General | General |
| 92 | - | General | General |
| 94 | - | General | General |
| 95 | - | General | General |
| 103 | - | General | General |
| 104 | - | General | General |
| 109 | - | General | General |
| 110 | - | General | General |

**Supplementary table 6. Study methodologies of 115 selected studies**

| **Article #** | **Methodology** | **Details** |
| --- | --- | --- |
| 35 | Quantitative | Survey |
| 95 | Quantitative | Survey |
| 60 | Quantitative | Survey |
| 2 | Quantitative | Survey |
| 63 | Quantitative | Survey |
| 36 | Quantitative | Survey |
| 115 | Quantitative | Survey |
| 14 | Quantitative | Survey |
| 101 | Quantitative | Survey |
| 20 | Quantitative | Survey |
| 81 | Quantitative | Survey |
| 109 | Quantitative | Survey |
| 80 | Quantitative | Survey |
| 46 | Quantitative | Survey |
| 88 | Quantitative | Survey |
| 40 | Quantitative | Survey |
| 59 | Quantitative | Survey |
| 102 | Quantitative | Survey |
| 64 | Quantitative | Survey |
| 57 | Quantitative | Survey |
| 42 | Quantitative | Survey |
| 106 | Quantitative | Survey |
| 82 | Quantitative | Survey |
| 100 | Quantitative | Survey |
| 55 | Quantitative | Survey |
| 27 | Quantitative | Survey |
| 30 | Quantitative | Survey |
| 85 | Quantitative | Survey |
| 103 | Quantitative | Survey |
| 92 | Quantitative | Survey |
| 86 | Quantitative | Survey |
| 51 | Quantitative | Survey |
| 38 | Quantitative | Survey |
| 26 | Quantitative | Survey |
| 7 | Quantitative | Survey |
| 94 | Quantitative | Survey |
| 70 | Quantitative | Survey |
| 5 | Quantitative | self-reported survey |
| 75 | Quantitative | questionnaire+survey |
| 76 | Quantitative | questionnaire+discrete choice experiment |
| 10 | Quantitative | questionnaire |
| 8 | Quantitative | questionnaire |
| 90 | Quantitative | questionnaire |
| 91 | Quantitative | questionnaire |
| 3 | Quantitative | questionnaire |
| 18 | Quantitative | questionnaire |
| 25 | Quantitative | questionnaire |
| 104 | Quantitative | questionnaire |
| 83 | Quantitative | questionnaire |
| 79 | Quantitative | questionnaire |
| 105 | Quantitative | questionnaire |
| 53 | Quantitative | questionnaire |
| 77 | Quantitative | questionnaire |
| 45 | Quantitative | questionnaire |
| 97 | Quantitative | questionnaire |
| 21 | Quantitative | questionnaire |
| 16 | Quantitative | questionnaire |
| 1 | Quantitative | questionnaire |
| 44 | Quantitative | questionnaire |
| 71 | Quantitative | questionnaire |
| 34 | Quantitative | questionnaire |
| 24 | Quantitative | questionnaire |
| 52 | Quantitative | questionnaire |
| 22 | Quantitative | questionnaire |
| 113 | Quantitative | questionnaire |
| 93 | Quantitative | questionnaire |
| 78 | Quantitative | questionnaire |
| 96 | Quantitative | questionnaire |
| 37 | Quantitative | questionnaire |
| 12 | Quantitative | questionnaire |
| 73 | Quantitative | questionnaire |
| 68 | Quantitative | questionnaire |
| 84 | Quantitative | questionnaire |
| 50 | Quantitative | questionnaire |
| 61 | Quantitative | questionnaire |
| 111 | Quantitative | questionnaire |
| 58 | Quantitative | questionnaire |
| 110 | Quantitative | questionnaire |
| 62 | Quantitative | questionnaire |
| 87 | Quantitative | questionnaire |
| 67 | Quantitative | questionnaire |
| 15 | Quantitative | Parental Needs Scale for Rare Diseases |
| 112 | Quantitative | Health Survey SF-36 |
| 114 | Quantitative | Health Survey SF-36 |
| 66 | Quantitative | Health Survey SF-36 |
| 47 | Quantitative | discrete choice experiment |
| 29 | Quantitative | discrete choice experiment |
| 65 | Quantitative | discrete choice experiment |
| 49 | Quantitative | Acceptance Illness Scale + the Health Survey SF-36 |
| 23 | Qualitative | discussions |
| 9 | Mixed | systematic review+survey; JLA methodology |
| 4 | Mixed | systematic review+questionnaire |
| 32 | Mixed | systematic review+discrete choice experiment |
| 74 | Mixed | systematic review+Delphi |
| 108 | Mixed | systematic review + questionnaire |
| 6 | Mixed | survey+discussion |
| 99 | Mixed | registry data+survey |
| 39 | Mixed | questionnaire+open ended questions |
| 28 | Mixed | questionnaire+open ended questions |
| 48 | Mixed | questionnaire+open ended questions |
| 13 | Mixed | lit review+survey |
| 19 | Mixed | lit review+survey |
| 31 | Mixed | lit review+discrete choice experiment |
| 98 | Mixed | interview+survey |
| 72 | Mixed | interview+survey |
| 43 | Mixed | interview+survey |
| 107 | Mixed | interview+questionnaire |
| 17 | Mixed | interview+questionnaire |
| 89 | Mixed | interview+questionnaire |
| 56 | Mixed | interview+questionnaire |
| 33 | Mixed | interview, observational study, survey |
| 69 | Mixed | focus group interview+survey |
| 41 | Mixed | focus group interview+survey |
| 11 | Mixed | focus group interview+questionnaire |
| 54 | Mixed | comments+survey |

**Supplementary table 7. Specific topics of the 115 selected surveys by participant types**

| **Study topics** | **Single Group** | | | | | | | | | | **Two groups or more** | | | | **Total** |
| --- | --- | --- | --- | --- | --- | --- | --- | --- | --- | --- | --- | --- | --- | --- | --- |
|  | **Group 1** | | | **Group 2** | | | | | **Group 3** | |  |  |  |  |  |
|  | **Families, Caregivers** | **Patients** | **Patients, Families, Caregivers** | **GPs** | **GPs, Other HCPs** | **Other HCPs** | **Specialists** | **Specialists, GPs** | **Funders** | **Public** | **Group 1, Group 2** | **Group 1, Group 3** | **Group 2, Group 3** | **Group 1, Group 2, Group 3** |  |
| **Experience** |  |  |  |  |  |  |  |  |  |  |  |  |  |  |  |
| access to information | 1 | 4 | 2 | 1 |  |  |  |  |  |  |  |  |  | 2 | **10** |
| burden of diseases, QoL | 6 | 15 | 5 |  |  |  |  |  |  |  | 1 |  |  |  | **27** |
| diagnosis | 4 | 6 | 3 |  |  |  |  |  |  |  | 2 |  |  |  | **15** |
| engagement | 1 |  |  |  |  |  |  |  |  |  |  |  |  |  | **1** |
| funding |  |  |  |  |  |  |  |  | 1 |  |  |  |  |  | **1** |
| health problems | 1 | 5 |  |  |  |  |  |  |  |  |  |  |  |  | **6** |
| self-efficacy |  | 1 |  |  |  |  |  |  |  |  |  |  |  |  | **1** |
| support | 1 | 1 |  |  |  |  |  |  |  |  |  |  |  |  | **2** |
| treatment |  |  | 2 | 3 |  |  | 6 |  |  |  |  |  |  |  | **11** |
| **Opinion** |  |  |  |  |  |  |  |  |  |  |  |  |  |  |  |
| access to information |  | 1 |  |  |  |  |  |  |  |  |  |  |  |  | **1** |
| burden of diseases, QoL |  |  |  |  |  |  |  |  |  |  | 1 |  |  |  | **1** |
| data sharing |  |  | 3 |  |  |  |  |  |  |  |  |  |  |  | **3** |
| diagnosis | 1 |  |  |  |  |  |  |  |  |  | 1 |  |  |  | **2** |
| engagement |  | 2 |  |  |  |  |  |  |  |  | 1 |  |  |  | **3** |
| funds |  |  |  |  |  |  |  |  |  | 4 |  |  |  |  | **4** |
| illness perception |  | 1 |  |  |  |  |  |  |  |  |  |  |  |  | **1** |
| research priorities |  |  |  |  |  |  |  |  |  |  | 1 |  |  |  | **1** |
| societal value |  | 1 |  |  |  | 1 |  |  |  |  |  |  |  |  | **2** |
| treatment | 1 |  | 1 |  |  |  |  | 1 |  | 2 |  | 1 |  |  | **6** |
| **Knowledge** | 1 |  | 1 |  | 1 |  | 2 | 2 |  |  |  |  | 2 |  | **9** |
| **Prevalence** | 1 | 4 | 1 |  |  |  | 1 |  |  |  |  |  | 1 |  | **8** |
| **Total** | **18** | **41** | **18** | **4** | **1** | **1** | **9** | **3** | **1** | **6** | **7** | **1** | **3** | **2** | **115** |

**Supplementary table 8. Excluded articles via screening (n=1,047)**

| **NO.** | **Exclusive criteria #** | **Authors** | **Title** | **Year** |
| --- | --- | --- | --- | --- |
| **1** | 1 | Medić B, Divac N, Stopić N, Savić-Vujović K, Glišić A, Cerovac N, et al. | The Attitudes of Medical Students Towards Rare Diseases: A Cross-Sectional Study | 2016 |
| **2** | 1 | Queiroz MC, Pedrosa RC, Berensztejn AC, Pereira Bde B, Nascimento EM, Duarte MM, et al. | Frequency of Cardiovascular Involvement in Familial Amyloidotic Polyneuropathy in Brazilian Patients | 2015 |
| **3** | 1 | Ayça A, Pınar G, Korcan D, Gönül Ç, Ayhan A, Ece B | Erken Başlangıçlı Graves Olgusu | 2013 |
| **4** | 1 | Ayça A, Pınar G, Korcan D, Gönül Ç, Ayhan A, Ece B | Infantile Graves Disease | 2013 |
| **5** | 1 | Ernst, G, Szczepanski, R, Lange, K | Patient Education Programs for Children, Adolescents and Parents - Survey on the Current Status of Concepts and Education Requirements in Germany | 2013 |
| **6** | 1 | Debrix I, André T, Flahault A, Kalu O, Gligorov J, Lotz JP, et al. | Anticancer Drugs Use Evaluation: Limits of the Approved Labeling | 2004 |
| **7** | 2 | Ballard LM, Horton RH, Dheensa S, Fenwick A, Lucassen AM | Exploring Broad Consent in the Context of the 100,000 Genomes Project: A Mixed Methods Study | 2020 |
| **8** | 2 | Şimşek İE, Şimşek TT, Erel S, Atasavun Uysal S | Factors Affecting Health Related Quality of Life and Depression Levels of Mothers in Families Having Children with Chronic Disabilities | 2020 |
| **9** | 2 | Inês M. et al | The Future Is Now: Discuss a Specific Program in Which the Leveraging of Data and Analytics Helped Uncover Insights That Might Have Been Missed in an Earlier Era | 2020 |
| **10** | 2 | Inês M, Coelho T, Conceição I, Ferreira L, de Carvalho M, Costa J | Health-Related Quality of Life in Hereditary Transthyretin Amyloidosis Polyneuropathy: A Prospective, Observational Study | 2020 |
| **11** | 2 | Rohner A, Gutbrod K, Kohler B, Lidzba K, Fischer U, Goeggel-Simonetti B, et al. | Health-Related Quality of Life in Young Adults Following Pediatric Arterial Ischemic Stroke | 2020 |
| **12** | 2 | Evans WRH, Tranter J, Rafi I, Hayward J, Qureshi N | How Genomic Information Is Accessed in Clinical Practice: An Electronic Survey of Uk General Practitioners | 2020 |
| **13** | 2 | Jandhyala R | Influence of Pharmaceutical Company Engagement Activities on the Decision to Prescribe: A Pilot Survey of Uk Rare Disease Medicine Prescribers | 2020 |
| **14** | 2 | Lacheta L, Dekker TJ, Goldenberg BT, Horan MP, Rosenberg SI, Pogorzelski J, et al. | Minimum 5-Year Clinical Outcomes, Survivorship, and Return to Sports after Hamstring Tendon Autograft Reconstruction for Sternoclavicular Joint Instability | 2020 |
| **15** | 2 | Izquierdo-García E, Escobar-Rodríguez I, Moreno-Villares JM, Iglesias-Peinado I | Social and Health Care Needs in Patients with Hereditary Fructose Intolerance in Spain | 2020 |
| **16** | 2 | Lagae L, Irwin J, Gibson E, Battersby A | Caregiver Impact and Health Service Use in High and Low Severity Dravet Syndrome: A Multinational Cohort Study\ | 2019 |
| **17** | 2 | Chim L, Salkeld G, Kelly PJ, Lipworth W, Hughes DA, Stockler MR | Community Views on Factors Affecting Medicines Resource Allocation: Cross-Sectional Survey of 3080 Adults in Australia | 2019 |
| **18** | 2 | Howell DA, Hart RI, Smith AG, Macleod U, Patmore R, Roman E | Disease-Related Factors Affecting Timely Lymphoma Diagnosis: A Qualitative Study Exploring Patient Experiences | 2019 |
| **19** | 2 | Rizzardo S, Bansback N, Dragojlovic N, Douglas C, Li KH, Mitton C, | Evaluating Canadians' Values for Drug Coverage Decision Making | 2019 |
| **20** | 2 | Horrow C, Pacyna JE, Cosenza C, Sharp R | Examining Physician Interactions with Disease Advocacy Organizations | 2019 |
| **21** | 2 | Rosell L, Wihl J, Hagberg O, Ohlsson B, Nilbert M | Function, Information, and Contributions: An Evaluation of National Multidisciplinary Team Meetings for Rare Cancers | 2019 |
| **22** | 2 | Cahan EM, Frick SL | Orthopaedic Phenotyping of Ngly1 Deficiency Using an International, Family-Led Disease Registry | 2019 |
| **23** | 2 | Bate J, Wingrove J, Donkin A, Taylor R, Whelan J | Patient Perspectives on a National Multidisciplinary Team Meeting for a Rare Cancer | 2019 |
| **24** | 2 | David MP, Funderburg A, Selig JP, Brown R, Calishak PM, Cove L, et al. | Perspectives of Patients with Dermatofibrosarcoma Protuberans on Diagnostic Delays, Surgical Outcomes, and Nonprotuberance | 2019 |
| **25** | 2 | Nagamatsu Y, Oze I, Aoe K, Hotta K, Kato K, Nakagawa J, et al. | Physician Requests by Patients with Malignant Pleural Mesothelioma in Japan | 2019 |
| **26** | 2 | Andolina JR, Burke MJ, Hijiya N, Chaudhury S, Schultz KR, Roth ME | Practice Patterns of Physician Treatment for Pediatric Chronic Myelogenous Leukemia | 2019 |
| **27** | 2 | Lee H, Kim SY, Kim G, Kang HY | Public Preferences for Corporate Social Responsibility Activities in the Pharmaceutical Industry: Empirical Evidence from Korea | 2019 |
| **28** | 2 | Hanisch M, Sielker S, Jung S, Kleinheinz J, Bohner L | Self-Assessment of Oral Health-Related Quality of Life in People with Ectodermal Dysplasia in Germany | 2019 |
| **29** | 2 | Gay EA, Byers PH, Bennett RL, Bird TD, Hisama FM | Trends over 42 Years in the Adult Medical Genetics Clinic at the University of Washington | 2019 |
| **30** | 2 | Gonano C, Pasquier J, Daccord C, Johnson SR, Harari S, Leclerc V, et al | Air Travel and Incidence of Pneumothorax in Lymphangioleiomyomatosis | 2018 |
| **31** | 2 | Stewart M, Shaffer S, Murphy B, Loftus J, Alvir J, Cicchetti M, et al. | Characterizing the High Disease Burden of Transthyretin Amyloidosis for Patients and Caregivers | 2018 |
| **32** | 2 | Dufendach KA, Timothy K, Ackerman MJ, Blevins C, Pflaumer A, Etheridge S, et al. | Clinical Outcomes and Modes of Death in Timothy Syndrome: A Multicenter International Study of a Rare Disorder | 2018 |
| **33** | 2 | Millogo T, Bicaba BW, Soubeiga JK, Dabiré E, Médah I, Kouanda S | Diabetes and Abnormal Glucose Regulation in the Adult Population of Burkina Faso: Prevalence and Predictors | 2018 |
| **34** | 2 | Takashima K, Maru Y, Mori S, Mano H, Noda T, Muto K. | Ethical Concerns on Sharing Genomic Data Including Patients' Family Members | 2018 |
| **35** | 2 | Slightam CA, Brandt K, Jenchura EC, Lewis ET, Asch SM, Zulman DM. | “I Had to Change So Much in My Life to Live with My New Limitations”: Multimorbid Patients’ Descriptions of Their Most Bothersome Chronic Conditions | 2018 |
| **36** | 2 | Haller J, David MP, Lee NE, Shalin SC, Gardner JM | Impact of Pathologist Involvement in Sarcoma and Rare Tumor Patient Support Groups on Facebook: A Survey of 542 Patients and Family Members | 2018 |
| **37** | 2 | Barashi NS, Pearce SM, Cohen AJ, Pariser JJ, Packiam VT, Eggener SE | Incidence, Risk Factors, and Outcomes for Rectal Injury During Radical Prostatectomy: A Population-Based Study | 2018 |
| **38** | 2 | Rocha HM, Savatt JM, Riggs ER, Wagner JK, Faucett WA, Martin CL | Incorporating Social Media into Your Support Tool Box: Points to Consider from Genetics-Based Communities | 2018 |
| **39** | 2 | Hua C, Bosc R, Sbidian E, De Prost N, Hughes C, Jabre P, Chosidow O, Le Cleach L | Interventions for Necrotizing Soft Tissue Infections in Adults | 2018 |
| **40** | 2 | Giménez-Ayala A, González-Brítez N, de- Arias AR | Knowledge, Attitudes, and Practices Regarding the Leishmaniases among Inhabitants from a Paraguayan District in the Border Area between Argentina, Brazil, and Paraguay | 2018 |
| **41** | 2 | Zolkipli-Cunningham Z, Xiao R, Stoddart A, McCormick EM, Holberts A, Burrill N, et al. | Mitochondrial Disease Patient Motivations and Barriers to Participate in Clinical Trials | 2018 |
| **42** | 2 | Mastboom MJ, Planje R, van de Sande MA | The Patient Perspective on the Impact of Tenosynovial Giant Cell Tumors on Daily Living: Crowdsourcing Study on Physical Function and Quality of Life | 2018 |
| **43** | 2 | Shaul E, Roth M, Lo Y, Geller DS, Hoang B, Yang R, et al. | Pediatric Oncologist Willingness to Offer Germline Tp53 Testing in Osteosarcoma | 2018 |
| **44** | 2 | Chen W, Mo S, Luo G, Wang Y, Deng X, Zhu J, et al. | Progressive Pseudorheumatoid Dysplasia with New-Found Gene Mutation of Wntl Inducible Signaling Pathway Protein 3 | 2018 |
| **45** | 2 | Dakić I, Arandjelović I, Savić B, Jovanović S, Tošić M, Kurucin T, et al. | Pulmonary Isolation and Clinical Relevance of Nontuberculous Mycobacteria During Nationwide Survey in Serbia, 2010-2015 | 2018 |
| **46** | 2 | Dzemaili S, Tiemensma J, Quinton R, Pitteloud N, Morin D, Dwyer AA | Beyond Hormone Replacement: Quality of Life in Women with Congenital Hypogonadotropic Hypogonadism | 2017 |
| **47** | 2 | Valkema PA, Luymes CH, Witteveen JE, le Cessie S, Appelman-Dijkstra NM, Hogendoorn PC, et al. | High Prevalence of Autoimmune Disease in the Rare Inflammatory Bone Disorder Sternocostoclavicular Hyperostosis: Survey of a Dutch Cohort | 2017 |
| **48** | 2 | Gemmell AP, Veach PM, MacFarlane I, Riesgraf R, LeRoy BS | If It Helps, It's Worth a Try: An Investigation of Perceptions and Attitudes About Genetic Counseling among Southern Manitoba Hutterites | 2017 |
| **49** | 2 | Mori Y, Downs J, Wong K, Anderson B, Epstein A, Leonard H | Impacts of Caring for a Child with the Cdkl5 Disorder on Parental Wellbeing and Family Quality of Life | 2017 |
| **50** | 2 | Shimizu S, Kobayashi T, Tomioka H, Ohtsu K, Matsui T, Hibi T | Involvement of Herbal Medicine as a Cause of Mesenteric Phlebosclerosis: Results from a Large-Scale Nationwide Survey | 2017 |
| **51** | 2 | Wang J, Al-Ouran R, Hu Y, Kim SY, Wan YW, Wangler MF, et al. | Marrvel: Integration of Human and Model Organism Genetic Resources to Facilitate Functional Annotation of the Human Genome | 2017 |
| **52** | 2 | Schmidt BM, Wrobel JS, Holmes CM | Physician Knowledge of a Rare Foot Condition - Influence of Diabetic Patient Population on Self-Described Knowledge and Treatment | 2017 |
| **53** | 2 | Ranft A, Seidel C, Hoffmann C, Paulussen M, Warby AC, van den Berg H, et al. | Quality of Survivorship in a Rare Disease: Clinicofunctional Outcome and Physical Activity in an Observational Cohort Study of 618 Long-Term Survivors of Ewing Sarcoma | 2017 |
| **54** | 2 | Seanehia J, Treibich C, Holmberg C, Müller-Nordhorn J, Casin V, Raude J, et al. | Quantifying Population Preferences around Vaccination against Severe but Rare Diseases: A Conjoint Analysis among French University Students, 2016 | 2017 |
| **55** | 2 | Gnanasakthy A, Mordin M, Evans E, Doward L, DeMuro C | A Review of Patient-Reported Outcome Labeling in the United States (2011-2015) | 2017 |
| **56** | 2 | Gupta N, Kopras EJ, Henske EP, James LE, El-Chemaly S, et al. | Spontaneous Pneumothoraces in Patients with Birt-Hogg-Dubé Syndrome | 2017 |
| **57** | 2 | Senier L, Lee R, Nicoll L | The Strategic Defense of Physician Autonomy: State Public Health Agencies as Countervailing Powers | 2017 |
| **58** | 2 | Neamsuvan O, Ruangrit T | A Survey of Herbal Weeds That Are Used to Treat Gastrointestinal Disorders from Southern Thailand: Krabi and Songkhla Provinces | 2017 |
| **59** | 2 | Dessens A, Guaragna-Filho G, Kyriakou A, et al. | Understanding the Needs of Professionals Who Provide Psychosocial Care for Children and Adults with Disorders of Sex Development | 2017 |
| **60** | 2 | Iskrov G, Ivanov S, Wrenn S, Stefanov R | Whole-Genome Sequencing in Newborn Screening-Attitudes and Opinions of Bulgarian Pediatricians and Geneticists | 2017 |
| **61** | 2 | Ingersoll B, Wainer AL, Berger NI, Pickard KE, Bonter N | Comparative Efficacy of Self-Directed & Therapist-Assisted Telehealth Parent Training Intervention for Children with Asd | 2016 |
| **62** | 2 | Beavers KM | Effect of High Protein Weight Loss for Seniors | 2016 |
| **63** | 2 | Pasick RJ, Joseph G, Stewart S, Kaplan C, Lee R, Luce J, et al. | Effective Referral of Low-Income Women at Risk for Hereditary Breast and Ovarian Cancer to Genetic Counseling: A Randomized Delayed Intervention Control Trial | 2016 |
| **64** | 2 | Kim H, Bar Ad V, McAna J, Dicker AP. | Evaluating the Quality, Clinical Relevance, and Resident Perception of the Radiation Oncology in-Training Examination: A National Survey | 2016 |
| **65** | 2 | Lion A, Schummer C, Delagardelle C, Urhausen A, Seil R, Theisen D | Promotion of Physical Activity in Patients with Non-Communicable Diseases in Luxembourg: A Follow-up of the Sport-Sante Inventory from 2014 | 2016 |
| **66** | 2 | Schrøder S, Homøe P, Wagner N, Vataire AL, Lundager Madsen HE, Bardow A | Does Drinking Water Influence Hospital-Admitted Sialolithiasis on an Epidemiological Level in Denmark? | 2015 |
| **67** | 2 | Monti G, Saccardo F, Castelnovo L, Novati P, Sollima S, Riva A, et al. | Fontan-Associated Protein-Losing Enteropathy and Plastic Bronchitis | 2015 |
| **68** | 2 | Ulm E, Feero WG, Dineen R, Charrow J, Wicklund C | Genetics Professionals' Opinions of Whole-Genome Sequencing in the Newborn Period | 2015 |
| **69** | 2 | Kent EE, Ambs A, Mitchell SA, Clauser SB, Smith AW, Hays RD | Health-Related Quality of Life in Older Adult Survivors of Selected Cancers: Data from the Seer-Mhos Linkage | 2015 |
| **70** | 2 | Riesgraf RJ, Veach PM, MacFarlane IM, LeRoy BS | Perceptions and Attitudes About Genetic Counseling among Residents of a Midwestern Rural Area | 2015 |
| **71** | 2 | Berody S, Galeotti C, Koné-Paut I, Piram M | A Restrospective Survey of Patients's Journey before the Diagnosis of Mevalonate Kinase Deficiency | 2015 |
| **72** | 2 | Kesselheim AS, Tan YT, Avorn J | The Roles of Academia, Rare Diseases, and Repurposing in the Development of the Most Transformative Drugs | 2015 |
| **73** | 2 | Hossain Shahcheraghi G, Javid M, Arasteh MM | Thromboembolic Disease after Knee Arthroplasty Is Rare in Southern Iran | 2015 |
| **74** | 2 | Rossi-Semerano L, Fautrel B, Wendling D, et al. | Tolerance and Efficacy of Off-Label Anti-Interleukin-1 Treatments in France: A Nationwide Survey | 2015 |
| **75** | 2 | Sundmacher L, Fischbach D, Schuettig W, Naumann C, Augustin U, Faisst C | Which Hospitalisations Are Ambulatory Care-Sensitive, to What Degree, and How Could the Rates Be Reduced? Results of a Group Consensus Study in Germany | 2015 |
| **76** | 2 | Cueva M, Cueva K, Dignan M, Lanier A, Kuhnley R | Evaluating Arts-Based Cancer Education Using an Internet Survey among Alaska Community Health Workers | 2014 |
| **77** | 2 | Dwyer AA, Quinton R, Morin D, Pitteloud N | Identifying the Unmet Health Needs of Patients with Congenital Hypogonadotropic Hypogonadism Using a Web-Based Needs Assessment: Implications for Online Interventions and Peer-to-Peer Support | 2014 |
| **78** | 2 | Henrike HW, Schultz C | The Impact of Health Care Professionals' Service Orientation on Patients' Innovative Behavior | 2014 |
| **79** | 2 | Monti G, Saccardo F, Castelnovo L, Novati P, Sollima S, Riva A, et al. | Prevalence of Mixed Cryoglobulinaemia Syndrome and Circulating Cryoglobulins in a Population-Based Survey: The Origgio Study | 2014 |
| **80** | 2 | Pabinger S, Dander A, Fischer M, Snajder R, Sperk M, Efremova M, et al. | A Survey of Tools for Variant Analysis of Next-Generation Genome Sequencing Data | 2014 |
| **81** | 2 | Hadker N, Egan J, Sanders J, Lagast H, Clarke BL | Understanding the Burden of Illness Associated with Hypoparathyroidism Reported among Patients in the Paradox Study | 2014 |
| **82** | 2 | Shams PN, Selva D; ANZSOPS Eyelid Retraction in Anophthalmia Survey Group | Upper Eyelid Retraction in the Anophthalmic Socket: Review and Survey of the Australian and New Zealand Society of Ophthalmic Plastic Surgeons (Anzsops) | 2014 |
| **83** | 2 | Lange M, Niedoszytko M, Renke J, Gleń J, Nedoszytko B | Clinical Aspects of Paediatric Mastocytosis: A Review of 101 Cases | 2013 |
| **84** | 2 | Oi N, Ohi K | Comparison of the Symptoms of Menopause and Symptoms of Thyroid Disease in Japanese Women Aged 35-59 Years | 2013 |
| **85** | 2 | de Groot EP, Duiverman EJ, Brand PL | Dysfunctional Breathing in Children with Asthma: A Rare but Relevant Comorbidity | 2013 |
| **86** | 2 | Gallin EK, Bond E, Califf RM, Crowley WF Jr, Davis P, Galbraith R, et al. | Forging Stronger Partnerships between Academic Health Centers and Patient-Driven Organizations | 2013 |
| **87** | 2 | Zhao W, Ye H, Zhao X, Zhang Z, Sun S, Jiang Y, et al. | A Network Investigation on Idiopathic Hypogonadotropic Hypogonadism in China | 2013 |
| **88** | 2 | Linley WG, Hughes DA | Societal Views on Nice, Cancer Drugs Fund and Value-Based Pricing Criteria for Prioritising Medicines: A Cross-Sectional Survey of 4118 Adults in Great Britain | 2013 |
| **89** | 2 | Wada-Isoe K, Ito S, Adachi T, Yamawaki M, Nakashita S, Kusumi M, et al. | Epidemiological Survey of Frontotemporal Lobar Degeneration in Tottori Prefecture, Japan | 2012 |
| **90** | 2 | Gilbert DL | Training the Next Generation of Child Neurologists: A Child Health-Based Approach | 2012 |
| **91** | 2 | Malcolm C, Forbat L, Anderson G, Gibson F, Hain R | Challenging Symptom Profiles of Life-Limiting Conditions in Children: A Survey of Care Professionals and Families | 2011 |
| **92** | 2 | Fehr A, Thürmann P, Razum O | Expert Delphi Survey on Research and Development into Drugs for Neglected Diseases | 2011 |
| **93** | 2 | Dallapiccola B, Torrente I, Agolini E, Morena A, Mingarelli R | A Nationwide Genetic Testing Survey in Italy, Year 2007 | 2010 |
| **94** | 2 | Kinder BW, Sherman AC, Young LR, Hagaman JT, Oprescu N, Byrnes S, et al. | Predictors for Clinical Trial Participation in the Rare Lung Disease Lymphangioleiomyomatosis | 2010 |
| **95** | 2 | de Beaucoudrey L, Samarina A, Bustamante J, Cobat A, Boisson-Dupuis S, Feinberg J, Al-Muhsen S, et al. | Revisiting Human Il-12rβ1 Deficiency: A Survey of 141 Patients from 30 Countries | 2010 |
| **96** | 2 | Bernal-Delgado E, Garcia-Armesto S, Oliva J, Sanchez Martinez FI, Repullo JR, et al. | Spain: Health System Review | 2010 |
| **97** | 2 | Milena M, Trofor A, Alexandrescu D, Balanescu O, Neagoe I, et al. | Survival Patients with Pulmonary Metastases in Testicular Cancer | 2010 |
| **98** | 2 | He S, Zurynski YA, Elliott EJ | What Do Paediatricians Think of the Australian Paediatric Surveillance Unit? | 2010 |
| **99** | 2 | Bager P, Meyer B, Brandt G | Assessment of the Need for Information among Patients with Neuroendocrine Tumors. A Pilot Study | 2009 |
| **100** | 2 | Bygum A | Hereditary Angio-Oedema in Denmark: A Nationwide Survey | 2009 |
| **101** | 2 | Muhm JM, Rock PB, McMullin DL, et al. | Effects of Aircraft Cabin Altitude on Passenger Comfort and Discomfort | 2006 |
| **102** | 2 | Mahmud FH, Murray JA, Kudva YC, Zinsmeister AR, Dierkhising RA, Lahr BD, et al. | Celiac Disease in Type 1 Diabetes Mellitus in a North American Community: Prevalence, Serologic Screening, and Clinical Features | 2005 |
| **103** | 2 | Ben Chaim J, Livne PM, Binyamini J, Hardak B, Ben-Meir D, Mor Y | Complications of Circumcision in Israel: A One Year Multicenter Survey | 2005 |
| **104** | 2 | Hirschhorn JN | Genetic and Genomic Approaches to Studying Stature and Pubertal Timing | 2005 |
| **105** | 2 | Sen S, Fawson P, Cherrington G, Friedman N, Maljanina R, Ftizner K, et al. | Patient Satisfaction Measurement in the Disease Management Industry | 2005 |
| **106** | 2 | Osborn JM, Stevenson TR | Pneumothorax as a Complication of Breast Augmentation | 2005 |
| **107** | 2 | Temte JL, Zinkel AR | The Primary Care Differential Diagnosis of Inhalational Anthrax | 2004 |
| **108** | 2 | Temte JL, Anderson AL. | Rapid Assessment of Agents of Biological Terrorism: Defining the Differential Diagnosis of Inhalational Anthrax Using Electronic Communication in a Practice-Based Research Network | 2004 |
| **109** | 2 | Sommer, F., Schwarzer, U., Wassmer, G. Bloch W, Braun M, Klotz T, et al. | Epidemiology of Peyronie's Disease | 2002 |
| **110** | 2 | Cullen RJ | In Search of Evidence: Family Practitioners' Use of the Internet for Clinical Information | 2002 |
| **111** | 2 | Schwarzer U, Sommer F, Klotz T, Braun M, Reifenrath B, Engelmann U | The Prevalence of Peyronie's Disease: Results of a Large Survey | 2001 |
| **112** | 2 | Yeaworth RC | Use of the Internet in Survey Research | 2001 |
| **113** | 3 | Heard JM, Vrinten C, Schlander M, Bellettato CM, VanLingen C, Scarpa M, et al. | Availability, Accessibility and Delivery to Patients of the 28 Orphan Medicines Approved by the European Medicine Agency for Hereditary Metabolic Diseases in the Metabern Network | 2020 |
| **114** | 3 | Kreuzer M, Drube J, Prüfe J, Schaefer F, Pape L; Members of the ERKNet Taskforce ‘QoL & Transition’ | Current Management of Transition of Young People Affected by Rare Renal Conditions in the Erknet | 2019 |
| **115** | 3 | Rowczenio D, Shinar Y, Ceccherini I, Sheils K, van Gijin M, Patton SJ, et al. | Current Practices for the Genetic Diagnosis of Autoinflammatory Diseases: Results of a European Molecular Genetics Quality Network Survey | 2019 |
| **116** | 3 | Yen E, Davis JM, Milne CP | Impact of Regulatory Incentive Programs on the Future of Pediatric Drug Development | 2019 |
| **117** | 3 | Sawai H, Oka K, Ushioda M, Nishimura G, Omori T, Numache H, et al. | National Survey of Prevalence and Prognosis of Thanatophoric Dysplasia in Japan | 2019 |
| **118** | 3 | Hong YD, Villalonga-Olives E, Perfetto EM | Patient-Reported Outcomes in Orphan Drug Labels Approved by the Us Food and Drug Administration | 2019 |
| **119** | 3 | Crowley S, Holgersen MG, Nielsen KG | Variation in Treatment Strategies for the Eradication of Pseudomonas Aeruginosa in Primary Ciliary Dyskinesia across European Centers | 2019 |
| **120** | 3 | Wong ZH, Davenport M | What Happens after Kasai for Biliary Atresia? A European Multicenter Survey | 2019 |
| **121** | 3 | Kamusheva M, Manova M, Savova AT, Petrova GI, Mitov K, Harsanyi A, et al. | Comparative Analysis of Legislative Requirements About Patients' Access to Biotechnological Drugs for Rare Diseases in Central and Eastern European Countries | 2018 |
| **122** | 3 | Kanamori Y, Watanabe T, Yorifuji T, Masue M, Sasaki H, Nio M | Congenital Hyperinsulinism Treated by Surgical Resection of the Hyperplastic Lesion Which Had Been Preoperatively Diagnosed by 18f-Dopa Pet Examination in Japan: A Nationwide Survey | 2018 |
| **123** | 3 | Mano H, Fujiwara S, Takamura K, Kitoh H, Takayama S, Ogata T, et al. | Congenital Limb Deficiency in Japan: A Cross-Sectional Nationwide Survey on Its Epidemiology | 2018 |
| **124** | 3 | Wood L, Bassez G, Bleyenheuft C, Campbell C, Cossette L, Jimenez-Moreno AC, et al. | Eight Years after an International Workshop on Myotonic Dystrophy Patient Registries: Case Study of a Global Collaboration for a Rare Disease | 2018 |
| **125** | 3 | Bertalan R, Lucas-Herald A, Kolesinska Z, Berra M, Cools M, Balsamo A, Hiort O | Evaluation of Dsd Training Schools Organized by Cost Action Bm1303 "Dsdnet" 11 Medical and Health Sciences 1117 Public Health and Health Services | 2018 |
| **126** | 3 | Reimer A, Bruckner-Tuderman L, Ott H | Mapping Health Care of Rare Diseases: The Example of Epidermolysis Bullosa in Germany 11 Medical and Health Sciences 1117 Public Health and Health Services | 2018 |
| **127** | 3 | Jahnel J, Zöhrer E, Fischler B, D’Antiga L, Debray D, Dezsofi A, et al. | Attempt to Determine the Prevalence of Two Inborn Errors of Primary Bile Acid Synthesis: Results of a European Survey | 2017 |
| **128** | 3 | Lazzarotto D, Candoni A, Filì C, Forghier F, Pagano L, Busca A, et al. | Clinical Outcome of Myeloid Sarcoma in Adult Patients and Effect of Allogeneic Stem Cell Transplantation. Results from a Multicenter Survey | 2017 |
| **129** | 3 | Blöß S, Klemann C, Rother AK, Mehmecke S, Schumacher U, Mucke U, et al. | Diagnostic Needs for Rare Diseases and Shared Prediagnostic Phenomena: Results of a German-Wide Expert Delphi Survey | 2017 |
| **130** | 3 | Minghetti P, Lanati EP, Godfrey J, Solà-Morales O, Wong O, Selletti S | From Off-Label to Repurposed Drug in Non-Oncological Rare Diseases: Definition and State of the Art in Selected Eu Countries | 2017 |
| **131** | 3 | De Sanctis V, Soliman AT, Canatan D, Elsedfy H, Karimi M, Daar S, et al. | An Icet- a Survey on Hypoparathyroidism in Patients with Thalassaemia Major and Intermedia: A Preliminary Report | 2017 |
| **132** | 3 | Roman H; FRIENDS group (French coloRectal Infiltrating ENDometriosis Study group) | A National Snapshot of the Surgical Management of Deep Infiltrating Endometriosis of the Rectum and Colon in France in 2015: A Multicenter Series of 1135 Cases | 2017 |
| **133** | 3 | Gong S, Wang Y, Pan X, Zhang L, Huang R, Chen X, et al. | The Availability and Affordability of Orphan Drugs for Rare Diseases in China | 2016 |
| **134** | 3 | Kyriakou A, Dessens A, Bryce J, Lotova V, Juul A, Krawczynski M, et al. | Current Models of Care for Disorders of Sex Development - Results from an International Survey of Specialist Centres | 2016 |
| **135** | 3 | Pinto D, Martin D, Chenhall R | The Involvement of Patient Organisations in Rare Disease Research: A Mixed Methods Study in Australia | 2016 |
| **136** | 3 | Nakano E, Masaki T, Kanda F, et al. | The Present Status of Xeroderma Pigmentosum in Japan and a Tentative Severity Classification Scale | 2016 |
| **137** | 3 | Habra O, Heinzer R, Haba-Rubio J, Rossetti AO | Prevalence and Mimics of Kleine-Levin Syndrome: A Survey in French-Speaking Switzerland | 2016 |
| **138** | 3 | Campo I, Luisetti M, Griese M, et al. | Whole Lung Lavage Therapy for Pulmonary Alveolar Proteinosis: A Global Survey of Current Practices and Procedures | 2016 |
| **139** | 3 | Hyry HI, Manuel J, Cox TM, Roos JC | Compassionate Use of Orphan Drugs | 2015 |
| **140** | 3 | Hattori M, Sako M, Kaneko T, Ashida A, Matsunaga A, Igarashi T, et al. | End-Stage Renal Disease in Japanese Children: A Nationwide Survey During 2006–2011 | 2015 |
| **141** | 3 | Williams DA, Bennett C, Bertuch A, Arcecti RJ, Bennett C, Bertuch A, et al. | Diagnosis and Treatment of Pediatric Acquired Aplastic Anemia (Aaa): An Initial Survey of the North American Pediatric Aplastic Anemia Consortium (Napaac) | 2014 |
| **142** | 3 | Žnidar I, Collin-Histed T, Niemeyer P,Parkkinene J, Lauridsen AG, Zarina S, et al. | The European Gaucher Alliance: A Survey of Member Patient Organisations' Activities, Healthcare Environments and Concerns | 2014 |
| **143** | 3 | Fischer KE, Rogowski WH | Funding Decisions for Newborn Screening: A Comparative Review of 22 Decision Processes in Europe | 2014 |
| **144** | 3 | McMillan K | Hidradenitis Suppurativa: Number of Diagnosed Patients, Demographic Characteristics, and Treatment Patterns in the United States | 2014 |
| **145** | 3 | Taguchi T, Kobayashi H, Kanamori Y, et al. | Isolated Intestinal Neuronal Dysplasia Type B (Ind-B) in Japan: Results from a Nationwide Survey | 2014 |
| **146** | 3 | Read N, Lim E, Tarzi MD, Hildick-Smith P, Burns S, Fidler KJ | Paediatric Hereditary Angioedema: A Survey of Uk Service Provision and Patient Experience | 2014 |
| **147** | 3 | Ampuero J, Bañales JM, Soriano G, Crespo J, Olcoz JL, Diago M, et al. | Polycystic Liver in the Adult (Pla) in Spain: Analysis of a Structured Survey Analysing the Experience and Attitude of Spanish Gastroenterologists | 2014 |
| **148** | 3 | Moore SW, Tshifularo N, Banieghbal B, Le Grange E, Millar A, Lakhoo K | Anorectal Atresia with Gross Terminal Colonic Distension in Africa | 2013 |
| **149** | 3 | Sfeir R, Bonnard A, Khen-Dunlop N, Auber F, Gelas T, Michaud L, et al. | Esophageal Atresia: Data from a National Cohort | 2013 |
| **150** | 3 | Walzer S, Travers K, Rieder S, Erazo-Fischer E, Matusiewicz D | Homozygous Familial Hypercholesterolemia (Hofh) in Germany: An Epidemiological Survey | 2013 |
| **151** | 3 | Angastiniotis M, Vives Corrons JL, Soteriades ES, Eleftheriou A | The Impact of Migrations on the Health Services for Rare Diseases in Europe: The Example of Haemoglobin Disorders | 2013 |
| **152** | 3 | Handfield R, Feldstein | Insurance Companies' Perspectives on the Orphan Drug Pipeline | 2013 |
| **153** | 3 | Tibussek D, Distelmaier F, von Kries R, Mayatepek E | Pseudotumor Cerebri in Childhood and Adolescence -- Results of a Germany-Wide Esped-Survey | 2013 |
| **154** | 3 | Hasegawa H, Kawasaki K, Inoue H, Umehara M, Takase M; Japanese Society of Pediatric Pulmonology Working Group (JSPPWG) | Epidemiologic Survey of Patients with Congenital Central Hypoventilation Syndrome in Japan | 2012 |
| **155** | 3 | Migita K, Uehara R, Nakamura Y, Yasunami M, Tsuchyua-Suzuki A, et al. | Familial Mediterranean Fever in Japan | 2012 |
| **156** | 3 | Sakushima Reinecke M, Rommel K, Schmidtke J.K, Tsuboi S, Yabe I, et al. | Nationwide Survey on the Epidemiology of Syringomyelia in Japan | 2012 |
| **157** | 3 | Reinecke M, Rommel K, Schmidtke J | Funding of Rare Disease Research in Germany: A Pilot Study | 2011 |
| **158** | 3 | Denis A, Mergaert L, Fostier C, Cleemput I, Simoens S | A Comparative Study of European Rare Disease and Orphan Drug Markets | 2010 |
| **159** | 3 | Winter Y, Schepelmann K, Spottke AE, Spottke AE, Claus D, Grothe C, et al. | Health-Related Quality of Life in Als, Myasthenia Gravis and Facioscapulohumeral Muscular Dystrophy | 2010 |
| **160** | 3 | Lainka E, Neudorf U, Lohse P, Timmann C, Stojanov S, Huss K, et al. | Incidence of Tnfrsf1a Mutations in German Children: Epidemiological, Clinical and Genetic Characteristics | 2009 |
| **161** | 3 | Gingter C, Wilm S, Abholz HH | Is Copd a Rare Disease? Prevalence and Identification Rates in Smokers Aged 40 Years and over within General Practice in Germany | 2009 |
| **162** | 3 | Neu A, Feldhahn L, Ehehalt S, Hub R, Ranke MB; DIARY group Baden-Württemberg | Type 2 Diabetes Mellitus in Children and Adolescents Is Still a Rare Disease in Germany: A Population-Based Assessment of the Prevalence of Type 2 Diabetes and Mody in Patients Aged 20 Years | 2009 |
| **163** | 3 | Herrgott I, Riemekasten G, Hunzelmann N, Sunderkötter C | Management of Cutaneous Vascular Complications in Systemic Scleroderma: Experience from the German Network | 2008 |
| **164** | 3 | Javeria, Samiullah, Marium, Neelofar Samad F, Babi G, et al. | Urbach-Wiethe Disease: Experience at a Tertiary Care Hospital in Abbottabad, Pakistan | 2008 |
| **165** | 3 | Delgado J, Villar A, Jimenez-Yuste V, Gago J, Quintana M, Hernandez-Navarro F | Phyllodes Tumours of the Breast | 2005 |
| **166** | 3 | Dionisi-Vici C, Rizzo C, Burlina AB, Gago J, Quintana M, Hernandez-Navarro F, et al. | Acquired Hemophilia: A Single-Center Survey with Emphasis on Immunotherapy and Treatment-Related Side-Effects | 2002 |
| **167** | 3 | Dionisi-Vici C, RizzoC, Burlina AB, Caruso U, Sabetta G, Uziel G, et al. | Inborn Errors of Metabolism in the Italian Pediatric Population: A National Retrospective Survey | 2002 |
| **168** | 4 | Johnson R, Fairchild A, Huang L, Srivastava A | Assessing Disease Experience across the Life Span for Individuals with Osteogenesis Imperfecta: Challenges and Opportunities for Patient-Reported Outcomes (Pros) Measurement: A Pilot Study | 2019 |
| **169** | 4 | Tan H, Yu K, Yu Y, An Z, Li J | 281. Understanding Patient Preferences for Meningococcal Serogroup B Vaccines | 2020 |
| **170** | 4 | Khan A, Gnanalingham K, Kearney T | A Rare Case of Isolated Hypothalamic-Pituitary Langerhans' Cell Histiocytosis | 2020 |
| **171** | 4 | Rasamison R, Alili JM, Berleur MP, Husson MC | Arginine and Citrulline: Survey on the Uses in French Hospitals | 2019 |
| **172** | 4 | Rhee J, Whitehead M, Fraser J, Tochen L | Clinical and Neuroimaging Features of Labrune Syndrome (Leukoencephalopathy with Calcifications and Cysts) | 2019 |
| **173** | 4 | Tiede A, Giangrande P, Teitel J, Amano K, Benso G, Nemes L, et al. | Delphi Expert Consensus Statements on Clinical Evaluation of Bleeds in Patients with Acquired Haemophilia | 2019 |
| **174** | 4 | Silva J, ferreira B, Resende G | Fertility-Preserving Surgery in Low-Grade Endometrial Stromal Sarcoma with Diffuse Peritoneal Disease | 2019 |
| **175** | 4 | Doğan Ü, Sertan D, Isik SR, Olgac M,Coskun, R, Terzioglu K, et al. | Hereditary Angioedema Awareness in Turkish Emergency Department Physicians and the Results of a Brief Video Assisted Education | 2019 |
| **176** | 4 | Sheard D, Greenfield J, Fere RL, Hall A, Griffiths A, Haughton M | How Do Patients and Caregivers Access Scientific Literature? | 2019 |
| **177** | 4 | Yarlas A, Kessler AS, Lovely A, Guthrie S, Pollock M, White MK, et al. | The Impact of Disease and Treatment on Generic Health-Related Quality of Life in Patients with Hattr Amyloidosis: Sf-36v2 Results from a Double-Blind Placebocontrolled Trial of Inotersen | 2019 |
| **178** | 4 | Rasamison R, Alili JM, Berleur MP, Husson MC | Isoleucine and Valine: Survey on the Uses in French Hospitals | 2019 |
| **179** | 4 | Rudebeck M, Scott C, Sireau N, Ranganath L | Patients' Experiences of Living with Alkaptonuria (Aku) from Diagnosis to Receiving Treatment and Care | 2019 |
| **180** | 4 | Skinner M, O'Hara J, Nugent D | Physical Activity and Treatment Adherence in Persons with Hemophilia Receiving Standard Half-Life (Shl) Vs. Extended Half-Life (Ehl) Replacement Factor Viii (Fviii): Findings from the Hemactive Patient Survey | 2019 |
| **181** | 4 | Samanek A, Waterton L | Rare Health Professionals | 2019 |
| **182** | 4 | Rajpurkar M, Williams S, Goldenberg NA, et al. | Results of an International Survey of Practice in the Diagnosis and Management of Pediatric Pulmonary Embolism | 2019 |
| **183** | 4 | Walker R, Jinnah H, Violante MR, Gonzalez C, Gatto E | Telemedicine Impact on Hemophilia Treatment Management | 2019 |
| **184** | 4 | Walker R, Jinnah H, Violante MR, Gonzalez C, Gatto E | MDS rare movement disorders study group global genetic testing survey: Exploring the unmet needs | 2019 |
| **185** | 4 | Vollstedt EJ, Kasten M, Klein C | Identifying genetic Parkinson's disease patients worldwide: Exploiting novel ways of team science | 2019 |
| **186** | 4 | Visser J, Perry L, Mitchell A, Bomken S, English M, Glaser A, et al. | A rare complication of a rare disease: Challenges in studying langerhans cell histiocytosis neurodegeneration in Uk and Ireland | 2019 |
| **187** | 4 | Villa S, Peyvandi F, De Cristofaro R, Di Minno G, Pippo L, Finardi A, et al. | PRO95 FROM DRUG ACCESS TO VALUE BASED HEALTHCARE: INTEGRATED CARE PATHWAYS FOR HEMOPHILIA IN ITALY | 2019 |
| **188** | 4 | Tsang VW, El-Sayegh L, Thompson C | Perceptions of transition from pediatric to adult care for youth with chronic and rare diseases: A participatory health research project | 2019 |
| **189** | 4 | Tomazos I, Sierra R, Cheung A, Brodsky R, Weitz I, Johnston K | PSY12 COST BURDEN OF BREAKTHROUGH HEMOLYSIS IN PATIENTS WITH PAROXYSMAL NOCTURNAL HEMOGLOBINURIA ON ECULIZUMAB TREATMENT | 2019 |
| **190** | 4 | Stouraca P, Bednarova J | The survey of autoimmune paraneoplastic neurological syndromes in the Southern Moravia region of the Czech Republic during 2005-2018 period | 2019 |
| **191** | 4 | Marchi E, Tobinai K, Maruyama D, Nagai H, O’Connor OA | Analysis of Published Treatment Options for Relapsed or Refractory (R/R) Peripheral T-Cell Lymphoma (Ptcl): An Evidence Based Decision Making Approach | 2019 |
| **192** | 4 | Rahman A, Choe M, Giza C, McArthur D, Wilson RB | Assessing Effectiveness of an Educational Intervention on Gaps in Child Neurology Knowledge for Pediatric Residents | 2019 |
| **193** | 4 | Kusunoki S, Yoshikawa K, Morikawa M, Kuwahara, M | Clinical Features of Bickerstaff Brainstem Encephalitis with or without Igg Antibodies against Gq1b and Glycolipid Complexes Containing Gq1b | 2019 |
| **194** | 4 | Kayikcioglu M, Kuman-Tunçel O, Pirildar S, Yilmaz M, Kaynar L, Aktan M, et al. | Clinical Management, Psychosocial Characteristics, and Quality of Life in Patients with Homozygous Familial Hypercholesterolemia Undergoing Ldl-Apheresis in Turkey: Results of a Nationwide Survey (a-Hit1 Registry) | 2019 |
| **195** | 4 | Matsumura T, Komakib H | The Effects of a Practical Guideline on Medical Management of Patients with Duchenne Muscular Dystrophy | 2019 |
| **196** | 4 | Parracciani M, Tona C, Gamba P, Dall’Oglio L, Conforti A, Cheli S | Esophageal Atresia and Long-Term Quality of Life in Italian Population: A Pilot Study | 2019 |
| **197** | 4 | Jesenk M, Kapustova L, Hrubiskova K, Dallos T, Banovcin P | Familial Mediterranean Fever in Slovakia-Clinical and Genetic Characteristics of Slovak Cohort | 2019 |
| **198** | 4 | Nuzzi A, Naor S, Inbar N, Gurevich T, Manor Y | A Global Survey on Speech Language Therapists' Awareness to Huntington's Disease | 2019 |
| **199** | 4 | Jensen E. Abonia J. Aceves S | High Patient-Reported Disease Burden for Eosinophilic Gastrointestinal Disorders | 2019 |
| **200** | 4 | Hamed N, Alimohammad M, Yasin KM, Saman N, Malek-hosseini, Ali S | Intestinal Failure Management in the Middle-Income Countries without Home Parenteral Nutrition | 2019 |
| **201** | 4 | Kruse A, Kruse C, Potthast N, Milligan K, Bussel JB | Mental Health and Treatment in Patients with Immune Thrombocytopenia (Itp); Data from the Platelet Disorder Support Association (Pdsa) Patient Registry | 2019 |
| **202** | 4 | Lazzarotto D, Candoni A, Melillo L, et al. | Multicenter Survey on Management and Outcome of Rare and Unpredictable Orphan Diseases: Adult Onset Histiocytoses | 2019 |
| **203** | 4 | Jimenez-Moreno AC, Gorman G | Neuromuscular Disorders and Prefer Project: Understanding Patient Preferences and Their Importance in the Drug Development Process | 2019 |
| **204** | 4 | Marchi E, Tobinai K, Maruyama D, Nagai H, O’Connor OA | An Objective Based Model of Published Treatment Options for Relapsed or Refractory (R/R) Peripheral T-Cell Lymphoma (Ptcl): An Evidence-Based Decisionmaking Approach | 2019 |
| **205** | 4 | Roberts S | Patient Advocacy Leadership in Advancing Clinical Recommendations in Ultra-Rare Disease: The Cln1 Batten Disease Experience | 2019 |
| **206** | 4 | Petkova V | A Pilot Questionnaire-Based Survey About Bulgarian Pharmacists' Opinion for Implementation of Pharmaceutical Care for Patients with Rare Diseases | 2019 |
| **207** | 4 | Pullen L, Picone M, Tan L, Johnston C, Stark H | Pitolisant Treatment Improves Multiple Clinical Symptoms of Prader-Willi Syndrome (Pws) in Children | 2019 |
| **208** | 4 | Jung G, Leinweber ME, Gray D, Derwich W, Grundmann RT, Schmitz-Rixen T | Popart—a Registry for Popliteal Artery Aneurysm Repair | 2019 |
| **209** | 4 | Lloyd A, Granerod J, Marshall JD, Domínguez-González C, Schneider-Gold C, Farrugia M | Pro119 a Novel Mapping Approach for Estimating Utilities in Non-Dystrophic Myotonia | 2019 |
| **210** | 4 | Lloyd AJ, Gallop K, Ali S, Myren KJ, Sierra JR, Anokhina K, Patriquin C, Hill A, Tomazos | Psy9 Preference Weights for Quality-Adjusted Life-Years Estimation for Treatments of Paroxysmal Nocturnal Hemoglobinuria in the United Kingdom | 2019 |
| **211** | 4 | Perry L, Visser J, Mitchell A, Bomken S, Enligh M, Glaser A, et al. | A Rare Complication of a Rare Disease-Challenges in Studying Langerhans Cell Histiocytosis Neurodegeneration in the UK | 2019 |
| **212** | 4 | Lei Q, Li C, Shi X | Remission of Paradoxical Skin Lesions by Tripterygium Wilfordii Hook F Treatment in Sapho Syndrome: A Case Report | 2019 |
| **213** | 4 | Mejias M, De Guevara ML, Soriano M, Seisdedos R | Use of Sirólimus 0.4% Ointment in Vascular Malformations | 2019 |
| **214** | 4 | Hayes DM, Rampon G, Rawal R, Buckhold FR | Addressing Resident-Identified Barriers to Attending Noon Conference | 2019 |
| **215** | 4 | Ishii T, Nakano E , Watanabe T , Higashi T | Analysis of Epidemiological Characteristics and Treatment for Male Breast Cancer Patients in Japan | 2019 |
| **216** | 4 | Curry M, Cruz R, Jarecki J, Belter L | Assessment of Awareness of the Early Clinical Features of Spinal Muscular Atrophy (Sma) Amongst Pediatricians | 2019 |
| **217** | 4 | Castro R, Dan D, Tschank J, Sparring V, Vana I, Glinsner B | Bridging the Gaps between Health, Social and Local Services, to Improve Care for People Living with Rare and Complex Conditions: Key Findings of the Eu-Funded Innovcare Project and Its Case Management Pilot | 2019 |
| **218** | 4 | Castaldo AJ, Jervelund C, Kirk AR, et al. | A Comprehensive Approach to Assessing the Value of Prophylactic Therapy for the Ultra Rare Disease Hereditary Angioedema Using Real World Patient Data | 2019 |
| **219** | 4 | Garg G, Hanna B, Tan NB, Gear R, Gabbett M | Creating a Bi-National Clinical Genetics Lecture Series: Its Successes and Challenges | 2019 |
| **220** | 4 | Duncan A, Kellum A, Peltier S, Cooper DL, Saad H | Disease Burden in Patients with Glanzmann Thrombasthenia: Perspectives from the Glanzmann Thrombasthenia Patient/Caregiver Questionnaire | 2019 |
| **221** | 4 | Yamaguchi I, Furusawa Y, Kawaguchi T, Yagishita N, Tanzawa K, Yamano Y, et al. | Establishment of a Comprehensive Information Infrastructure and a Support Organization for Rare Disease Research in Japan (Raddar-J)...The 17th World Congress of Medical and Health Informatics, 25-30 August 2019, Lyon, France | 2019 |
| **222** | 4 | Giannuzzi V, Felisi M, Devlieger H, et al. | Ethical Issues and Barriers for Multi-National Paediatric Clinical Trials: The Challenging Experience of the Deep Project | 2019 |
| **223** | 4 | Alsaffar H, Elawwa AS, Aljaser F, Chaturvedi D | High Demand for Collaborative Work between Paediatric Endocrinologists in Arab Countries | 2019 |
| **224** | 4 | Marchi E, Tobinai K, Maruyama D, Nagai H, O'Connor OA | Hui, Wpai, and Neuromuscular Pedsql Scores in Patients with Spinal Muscular Atrophy: Findings from the 2019 Cure Sma Community Update Survey | 2019 |
| **225** | 4 | Izquierdo MCP, Rio-Garma JD, Rubia JDL, et al. | Incidence, Diagnosis, and Outcome of Acquired Thrombotic Thrombocytopenic Purpura (Attp): A Nationwide Survey by the Spanish Apheresis Group | 2019 |
| **226** | 4 | Ishihara T, Soejima T, Maebayashi K, Yamamoto T, Kohzuki H, Okada K, Hara J, Nishikawa R | Investigation of Recurrence Factors in Patients Who Received Radiotherapy for Ependymoma: A Retrospective Multicenter Analysis Conducted by the Japan Children's Cancer Group | 2019 |
| **227** | 4 | Bajbaj A, Vitali C. Cuchel M, Rader D | Literature Survey of Lcat Deficiency: Natural History and Biomarker Identification | 2019 |
| **228** | 4 | Celano A, Mingolla S, Santopietro M | Living with a Rheumatic Disease: A Research About Quality of Life and Work Conditions | 2019 |
| **229** | 4 | Henriques BJ, Lucas TG, Martins E, Gaspar A, Banderia A, Nogueria C, et al. | Molecular and Clinical Investigations on Portuguese Patients with Multiple Acyl-Coa Dehydrogenase Deficiency | 2019 |
| **230** | 4 | Brook R, Sax MJ, Carlisle JA, Smeeding JE | Pns113 the 2019 United States Payor Landscape: Trends and Results from Surveys on Formulary Management | 2019 |
| **231** | 4 | Dheensa S, Sterckx S, Cockbain J, Fenwick A, Lucassen A | Pooling Genomic Data into a "National Data Lake": The First Steps Towards Establishing a Uk Learning Healthcare System | 2019 |
| **232** | 4 | Boussahoua M, Godard C, Tehard B | Pro122 Economic Evaluation of Nusinersen in Spinal Muscular Atrophy: A Comparison of Nice and Has Opinions | 2019 |
| **233** | 4 | Dolezalova P, Little M, Wulffraat N | Rare Is What Matters: Childhood Vasculitis in Eu Projects | 2019 |
| **234** | 4 | Goutaki M, Eich MO, Halbeisen FS, Barben J, Casaulta C, Clarenbach C, et al. | The Swiss Primary Ciliary Dyskinesia Registry: An Update | 2019 |
| **235** | 4 | Hsairi M, Gargouri L, Rgaieg C | Wilson's Disease in Children: About 8 Cases | 2019 |
| **236** | 4 | Yarlas A, Guthrie S, Pollock M, Lovley A, White MK | Pro59 Association between Clinical Outcomes and Quality of Life in Patients with Hereditary Transthyretin Amyloidosis | 2019 |
| **237** | 4 | Yooo J, Campwala I, Gupta S | Comparing What Clinicians Believe with Actual Patient Experiences: A Crowd Sourcing Study on Hidradenitis Suppurativa | 2018 |
| **238** | 4 | Sherber RM, Whyte LJ, Woehrle M, Duekck AC | Creation and Validation of the “Mympn” Prospective Patient Registry of Myeloproliferative Neoplasm Patients | 2018 |
| **239** | 4 | Yarlas A, Lovley A, Guthrie S, Pollock M, Sikora Kessler A, White M | Disease Burden as Assessed by the Norfolk Quality of Life Scale in Hereditary Transthyretin Amyloidosis Relative to Diabetic Neuropathy | 2018 |
| **240** | 4 | Sangiorgi L, De La Calle M | European Reference Network on Bone Rare Diseases (Bond Ern) | 2018 |
| **241** | 4 | Valerie Salvatore V, Alan Gilstrap A, Karren R Williams KR, et al. | Evaluating the Impact of Peer Support and Connection on the Quality of Life of Patients with Familial Chylomicronemia Syndrome | 2018 |
| **242** | 4 | Yadav D, Kumar R, Bal C | F-18 Fdg Pet/Ct Imaging in Staging and Response Assessment in Children with Langerhans Cell Histiocytosis | 2018 |
| **243** | 4 | Serra M, Morgunovsky-Michell I, Barrientos S | How Much Do Physicians Know About Hht? A Survey-Based Cross Sectional Study | 2018 |
| **244** | 4 | Yarlas A, Sikora Kessler A, Lovley A, Guthrie S, Pollock M, White MK | Impact of Inotersen on Generic Health-Related Quality of Life for Patients with Hattr Amyloidosis: Results from a Double-Blind Placebo-Controlled Trial | 2018 |
| **245** | 4 | Wirrell EC, Vanderwiel AJ, Nickels L, Vanderwiel SL, Nickels KC | Impact of Prior Authorization of Aeds on Children with Epilepsy | 2018 |
| **246** | 4 | Polydefis M, Cruz MW, Dyck P | Inotersen Improved Norfolk Quality of Life-Diabetic Neuropathy (Norfolk Qol-Dn) Measures in Patients with Hereditary Transthyretin (Hattr) Amyloidosis Treated in the Phase-3 Study Neuro-Ttr | 2018 |
| **247** | 4 | Zurynski YA, Deverell M, Christodoulou J, Leonard H, Dalkeith T, Johnson S, Elliott EJ | It's Worse When You're Sick in the Country: Access Barriers and High out-of-Pocket Health Costs for Children with Rare Chronic and Complex Diseases | 2018 |
| **248** | 4 | Yamamoto N, Ohashi N, Kato K | Making Supportive Guidance for Gathering and Sharing Clinical and -Omics Data to Develop Next-Generation Medicine under the Recent Legislative Efforts to Protect Personal Information | 2018 |
| **249** | 4 | Spencer-Tansley R, Hunter A | Mental Health and Rare Diseases: Mixed Methods Study and Policy Recommendations | 2018 |
| **250** | 4 | González GM, Casillas-Vega N, Garza-González E, et al. | Molecular Typing of Clinical Cryptococcus Neoformans and C. Gattii Species Complexes and Cryptococcal Antigen Screening Program in Iran-What Can We Do?...5th Iranian Congress in Medical Mycology, Tehran, Iran, Dec 4-6, 2018 | 2018 |
| **251** | 4 | Schroder C, Cardoso F, Dijkstra N | Quality of Life (Qol) in Male Breast Cancer (Bc): Prospective Study of the Eortc10085/Tbcrc029/Big2-07/Nabcg International Male Bc Program | 2018 |
| **252** | 4 | Sparring V, Tschank J, Handler K, Mansoory E | Social and Economic Impact of a Case Management Approach for People with Rare and Complex Conditions in Salaj, Romania | 2018 |
| **253** | 4 | Suzuki H, Matsuzaki J, SHimoda M, Mori H, Fukuhara S, Kanai Y, Kanai T | Specific Prediction of Duodenal Tumor Using Demographic and Endoscopic Characteristics: An Age-and Sex-Matched Case-Control Study | 2018 |
| **254** | 4 | Vassoler H, Spoors J | Tools of the Trade: Payer Approaches to High Patient Volume, Premium Priced Therapies | 2018 |
| **255** | 4 | Zhang X, Zhang L, Gijsen M, Cong Z | Treatment Pattern and Unmet Need in Adults with Philadelphia Positive (Ph+) Relapsed or Refractory (R/R) B-Cell Precursor (Bcp) Acute Lymphoblastic Leukemia (All) in Eu-5 Countries | 2018 |
| **256** | 4 | Schiffmann I, Freund M, Engels K, Weierstall R | Understanding Magnetic Resonance Imaging in Multiple Sclerosis (Umims): Effect of an Interactive Online Education Tool on Emotions, Attitude and Knowledge in People with Multiple Sclerosis | 2018 |
| **257** | 4 | Upadhyay Z, Vani H, Raghupathy P | Unusual and Rare Association of Type I Diabetes Mellitus with Epiphyseal Dysplasia | 2018 |
| **258** | 4 | Shovlin CL, Botella L, Geisthoff, UW | Vascern Hht Priority Evaluations 2016-2017 | 2018 |
| **259** | 4 | Sahu M, Wells A, Wolfe L, Game D, Thomas K, Bultitude M | Are Patient Information Days Useful for Patients with Cystinuria?: A Patients' Perspective | 2018 |
| **260** | 4 | Robinson L, Ellison E, Rooney R, Brown C, Arnold S | Assessing Consenters Attitudes and Opinions of Genetic Biorepository Research in Children | 2018 |
| **261** | 4 | Li J | Clinical Characteristics and Treatment of 25 Children with Systemic Lupus Erythematosus Complicated with Thrombotic Microangiopathy in China | 2018 |
| **262** | 4 | Calais JM, Debza Y, Leroy C, Maurey H, Wassmer E, Deiva K | Clinical Trials in Pediatric Multiple Sclerosis : A British-French Patient and Parent Perspective | 2018 |
| **263** | 4 | Narayanan S, Olesen D | Diagnosis of Patients with Duchenne Muscular Dystrophy (Dmd): Results from a Global Survey of Healthcare Providers from Nine Countries | 2018 |
| **264** | 4 | Lovley A, Guthrie S, Pollokc M, White M, Sikora Kessler A, Yarlas A | Disease Burden in Patient Subgroups of Hereditary Transthyretin Amyloidosis | 2018 |
| **265** | 4 | Ramchandren S, Tirella C | Dysautonomia in Charcot-Marie-Tooth Disease and Correlations with Patient-Reported Disability | 2018 |
| **266** | 4 | Pierre-Louis MS, Malidor JJ, Pierre-Louis L | Establishment of Oral Care for Caribbean Patients with Congenital Blood Coagulation Deficiency | 2018 |
| **267** | 4 | Rahman A, Choe M, Bhatt Wilson R | Identifying Gaps in Education in Child Neurology for Pediatric Residents at a Quaternary Care Academic Hospital | 2018 |
| **268** | 4 | Maza G*,* Yanez-Siller JC*,* Subramaniam S*,* Otto BA*,* Daniel PM*,* Carrau RL | Incidence of Empty Nose Syndrome Following Endoscopic Endonasal Skull Base Surgery: A Preliminary Trial | 2018 |
| **269** | 4 | Moore-Hepburn C | Influencing the National Debate on Medical Assistance in Dying (Maid): Leveraging the Canadian Paediatric Surveillance System (Cpsp) to Inform the Position of the Canadian Paediatric Society (Cps) | 2018 |
| **270** | 4 | Lommelé A, De Schoolmeester M | Involving Patients in Research from an Industry-Sponsored Registry | 2018 |
| **271** | 4 | Quock T | Overcoming Research Challenges in Observational Studies of Rare Diseases: Lessons Learned from Amyloid Light Chain Amyloidosis | 2018 |
| **272** | 4 | Nestler-Parr S, Upadhyaya S | Patient Goup Experiences of Engagement in Health Technology Appraisals for Rare Disease Treatments-a Pilot Study in the Uk | 2018 |
| **273** | 4 | Morrison A, Weigl M, Prӓhofer A | Patient Organisations Working in Partnership to Research the Patient Experience of Rare Diseases: The Mps Iii Survey | 2018 |
| **274** | 4 | Müller von der Grün J, Bon D, Rödel C, Balermpas P | Patterns of Care Analysis for Head & Neck Cancer of Unknown Primary Site: A Survey inside the German Society of Radiation Oncology (Degro) | 2018 |
| **275** | 4 | Palma JA, Krismer F, Meissner W, Kaufmann H, Norcliffe-Kaufmann L | Preliminary Results of the Global Multiple System Atrophy Registry: An Internet-Based Patient-Reported Registry | 2018 |
| **276** | 4 | Regier DS, Ottolini M, Summar M | Rare Diseases Research Certificate Program: Equipping the Next Generation in Rare Disease Research | 2018 |
| **277** | 4 | Glenn TL, Pereira N, Madeira J, et al. | Reproductive Endocrinology Infertility (Rei) Specialists' Utilization and Attitudes Towards Expanded Carrier Screening for Oocyte Donors | 2018 |
| **278** | 4 | Sasaki A, Ogawa M, Tono C, Obara S, Hosoi N, Wakabayashi G | Single-Port Versus Multiport Laparoscopic Cholecystectomy - a Randomized Controlled Trial with Long-Term Follow-Up | 2018 |
| **279** | 4 | Mason N, Parviainen L, Withe M, Porter J | Treatment Effects in Paediatric Adrenal Insuficiency and Associated Resource Use: A Delphi Study | 2018 |
| **280** | 4 | Pelentsov L | The Uk Haemophilia Parental Support Study - Are Families with Children with Bleeding Disorders Being Supported Appropriately? A Single Site Pilot Study Using the Parental Needs Scale for Rare Diseases (Pns-Rd) | 2018 |
| **281** | 4 | Matsushima M, Sakushima K, Yabe I, et al. | Usefulness of a Simple Symptom Assessment Scale for Multiple System Atrophy: Data from Horc-Msa Project | 2018 |
| **282** | 4 | Creekmore J, Hayes H, Bromberg M, Gibson S, Leatham J | Awareness and Understanding of Amyotrophic Lateral Sclerosis in Newly Diagnosed Patients, Family, and Friends | 2018 |
| **283** | 4 | Conceicao I, Berk J, Wang A | Baseline Characteristics of Patients with Hereditary Transthyretin (Hattr) Amyloidosis with Polyneuropathy Enrolled in the Phase 3 Study Neuro-Ttr Demonstrate Significant Disease Burden | 2018 |
| **284** | 4 | Boyd J, Cunningham S, Schwerk N, Gilbert C, Powell P, Schamberger M | Challenges Facing Parents/Caregivers of Children with Interstitial Lung Disease (Child): International Survey | 2018 |
| **285** | 4 | Kim E, Bae S, Jeong D, Argyropoulos A, Chen S, Tan B, Kim B | The Comparative Analysis of Risk-Sharing Agreements in Korea, Australia, Taiwan Focusing on Operation | 2018 |
| **286** | 4 | Fingerhutova S, Franova J, Hlavackova E, Jancova E, Prochazkova L, Tesarova M, et al. | Cryopyrinopathy across Generations: Longterm Disease Outcome | 2018 |
| **287** | 4 | Hittinger A, Boiu S, Bader-Meunier B, Carine W, Quartier-Dit-Martire P | Diversity in Presentation and Evolution of Autoinflammatory Diseases in a French Pediatric Cohort of Referral Center of Rheumatology | 2018 |
| **288** | 4 | Doyle S, Moss E, Hartley L, Knigth C, Bell J, Ahdesmaki O, et al. | An Economic Systematic Literature Review of Eosinophilic Granulomatosis with Polyangiitis | 2018 |
| **289** | 4 | Daghbashyan S, Melkikyan N, Sahakyan L, Tovmasyan A | A Few Words About Aplastic Anemia in Armenia | 2018 |
| **290** | 4 | Desyatik EA, Shadrivova OV, Borzova JV, et al. | First Case of Non-Imported Pulmonary Coccidioidomycosis in Russia | 2018 |
| **291** | 4 | Kruse A, Kruse C, Lambert MP | The Itp Natural History Study Registry: Preliminary Findings on the Immune Thrombocytopenia Patient Experience | 2018 |
| **292** | 4 | Hua L | Level of Serum 25(Oh)D with Disease Activity of a Cohort Study | 2018 |
| **293** | 4 | Iberahim Shah MF, Wahid AMA, Baharin SS, Jonathan R | Osteopoikilosis: A Benign Bone Spots | 2018 |
| **294** | 4 | D'Souza A | Patient-Reported Outcomes in Clinical Care and Clinical Trial Settings: A Clinician's Experience in Amyloid Light Chain (Al) Amyloidosis | 2018 |
| **295** | 4 | Dekker HK, Effing-Boele MC, Hammann MJA | Patients Experiences Are Essential for Improving the Quality of Health Care | 2018 |
| **296** | 4 | Finnegan T, Maeglin J | Practice Patterns and Knowledge of Spinal Muscular Atrophy Care among Pediatricians and Neurologists | 2018 |
| **297** | 4 | Guth A, Cheikh N, Vieux R, Thierry L, Leverger G, Bertrand Y, et al. | Predictive Factors for Developing Systemic Lupus Erythematosus after Childhood Autoimmune Cytopenia | 2018 |
| **298** | 4 | Kay D, Urbine DR | Pulmonary Nodular Lymphoid Hyperplasia Associated with Multiple Sclerosis | 2018 |
| **299** | 4 | Evers P, Jansen A | Quality of Life in Health Technology Assessment: The Dutch Experience | 2018 |
| **300** | 4 | Dubey S, Gelder C, Wong R, Shakespeare J, Hart N, D'Cruz D | Six Cases of Relapsing Polychondritis Presenting as Severe Airways Disease to a Secondary Care Centre in One Year | 2018 |
| **301** | 4 | Feinmann JC, Rivett A, Souto M,et al. | Survey of the Nhs Care Experience of People Living with Rare Autoimmune Rheumatic Diseases | 2018 |
| **302** | 4 | Chen K, Hadker N, Abibova I, Hanbury-Brown L, Vera-Llonch M, Clarke B | Symptom Burden and Hrqol Reported among Patients with Chronic Hypoparathyroidism: Impact of Treatment with Rhpth (1-84) and with Standard Therapy | 2018 |
| **303** | 4 | Diling D, Nair A, Gries CJ, Leard LE, et al. | Use of Sirolimus in Patients with Lymphangioleiomyomatosis (Lam) on Waiting Lists for Lung Transplant (Ltx) | 2018 |
| **304** | 4 | Buscarini E, Botella LM, Geisthoff UW | Vascern Hht Survey 2: Drug Registry-Part 1 | 2018 |
| **305** | 4 | Ozcan MA, Davulcu EA, Ulusoy Y | Adult Onset Langerhans Cell Histiyocytosis: A Single Center Experience | 2018 |
| **306** | 4 | Yarlas A, Sikora Kessler A, Lovley A , Guthrie S , Pollock , White MK | Analysis of Responses to Sf-36v2 Items for Patients with Hattr Amyloidosis: Results from a Double-Blind Placebo-Controlled Trial | 2018 |
| **307** | 4 | Boulanger V, Seebald A, Rossov S | Building a Rare Disease Research Consortium with Harmonized, Patient-Powered Natural History Registries | 2018 |
| **308** | 4 | Boother EJ, Shovlin CL | The Characterisation and Investigation of Pulmonary Arteriovenous Malformations and Cerebral Abscess Risk Factors within a Rare Vascular Disease: Results from a Targeted 125-Question, International Patient Survey | 2018 |
| **309** | 4 | Boxell E, Bernarde J, Rzepa E, Baldock L, Rylands AJ | Early Engagement of the “Patient Voice” to Inform Patient Reported Outcome (Pro) Measurement in Global Pivotal Treatment Trial of Cholangiocarcinoma (Cca) | 2018 |
| **310** | 4 | Yarlas A, Merlini G, White MK, et al. | Impact of Inotersen on Subgroups of Patients with Hereditary Ttr Amyloidosis: Results from a Double-Blind Placebo-Controlled Trial | 2018 |
| **311** | 4 | Adachi T, Kawamura K, Furusawa Y | Japans Initiative on Rare and Undiagnosed Diseases Patients: To Bring Their Diagnostic Odyssey to an End, and Beyond | 2018 |
| **312** | 4 | Agbo-Kpati KP, Quartier P, Breton S, Bader-Meunier B | Muscle Aseptic Abscesses in Pediatric Patients Diagnosed with Systemic Onset Juvenile Idiopathic Arthritis or Polyarthritis: Is It a New Auto-Inflammatory Disease? | 2018 |
| **313** | 4 | Ballinger R, Eliasson L | Pilot Study to Assess Impacts on Researchers of Conducting Emotionally Challenging Research from Consultancy or Pharmaceutical Settings | 2018 |
| **314** | 4 | Sharda AV, Bauer KA | Aspirin Thromboprophylaxis in Joint Replacement Surgery | 2017 |
| **315** | 4 | Shah R, Bentley JP, Banahan B, Patel A, Holmes E, Barnard M, Khanna R | An Assessment of Psychosocial Determinants of Healthrelated Quality of Life (Hrqol) among Adults with Hemophilia in the United States | 2017 |
| **316** | 4 | Upton CM, Wordsworth J, Cork D, Ralston S | Assessment of Public Opinion Regarding the Ethics of Nice Cdf, Hst, and End-of-Life Criteria for Drug Reimbursement | 2017 |
| **317** | 4 | Stewart M, Mundayat R, Alvir J, Grima D, Tran D, Ong M, et al. | Clinical Characteristics and Health State Utilities in Patients with Transthyretin Familial Amyloid Polyneuropathy in Brazil | 2017 |
| **318** | 4 | Wu CCC, Econs MJ, NDi Meglio LA, Insogna K, Levine MA, Orchard P, et al. | Consensus Guidelines on the Medical Management of Osteopetrosis | 2017 |
| **319** | 4 | Zulian F | Consensus-Based Recommendations (Share) for the Management of Juvenile Scleroderma | 2017 |
| **320** | 4 | Yeh S | Ebola Virus Disease: What Do Physicians Know and Are They Willing to Provide Care? | 2017 |
| **321** | 4 | Vu H, Gaitonde P,Shaya F, Rodriguez De Bittner M | Evaluation of Outcomes among Hiv Patients Taking Antiretroviral Regimens Using the National Ambulatory Health Care Survey (Namcs) Data | 2017 |
| **322** | 4 | ​Zabrodska L | Hereditary Angioedema in Ukraine: A National Survey | 2017 |
| **323** | 4 | Subramanian L, Wei Y,Nguyen C,Hicks R, P Chitra P, Campbe[l](https://www.cambridge.org/core/search?filters%5BauthorTerms%5D=C%20Campbell&eventCode=SE-AU) C | Myopathic Aspects of Mowat-Wilson Syndrome | 2017 |
| **324** | 4 | Schmoeller M, Ernst F, Gee A, Hooten J | Quantitative Assessment of Ophthalmologist Prescribing Patterns for Severe Vernal Keratoconjunctivitis (Vkc) to Inform Payer Decision Making in the Eu | 2017 |
| **325** | 4 | Yu J, Paranagama D, Parasuraman S | Recruitment Strategies and Geographic Representativeness for Patient Surveys in Rare Diseases | 2017 |
| **326** | 4 | Stringer KA, Schumacher, KR, Caruthers RL, Nasr SZ, Myers JL | Study Design: Phase Ii Safety and Efficacy of Inhaled Tissue Plasminogen Activator (Activase) for the Treatment of Acute Pediatric Plastic Bronchitis | 2017 |
| **327** | 4 | Quock TP, McCausland KL, Yokota M, Bayliss M, Guthrie S, White MK | Work Productivity and Impairment among Patients with Light Chain Amyloidosis | 2017 |
| **328** | 4 | Kragl B, Wassenberg C, Steinmetz HT, Springer G, Jentisch-Ullrich K, Gaumann A, et al. | Analyses of 845 Patients with Pmf, Pet-Mf and Ppv-Mf Treated in 35 German Hematology Centers - a Retrospective Field Study | 2017 |
| **329** | 4 | Ing SW, Clarke BL, Khan AA, Mannstadt M, McDermott MT, Piccolo R, et al. | Chronic Hypoparathyroidism Disease Profile: Initial Analysis from the Paradighm™ Natural History Global Registry | 2017 |
| **330** | 4 | Muenzer J, Giugliani R, Scarpa M, et al. | Clinical Outcomes after 3 Years of Idursulfase Treatment in Patients with Mps Ii: Data from the Hunter Outcome Survey (Hos) | 2017 |
| **331** | 4 | Kohalmi KV, Andrási N, Szilágyi T, Veszeli N, Varga L, Farkas H | Comorbidities of Patients with Hereditary Angioedema Due to C1-Inhibitor Deficiency in Hungary | 2017 |
| **332** | 4 | Pathirana L, Sekhar M, Patch D | Development of a Mobile Phone App to Empower and Support Self-Monitoring in Patients with Myeloproliferative Neoplasm and Splanchnic Vein Thrombosis (Mpn-Svt) | 2017 |
| **333** | 4 | Hu G, Koganov M, Shinozaki R, Harigaya K | Differences in Timing of Memantine Initiation in Mild-Moderate and Moderate-Severe Alzheimer's Patients in USA Vs. Japan | 2017 |
| **334** | 4 | Khan M, Chaudary N | Effect of Early Family Meetings and Patient Education in Adults with Cystic Fibrosis: A Quality Improvement Initiative | 2017 |
| **335** | 4 | Kim JH, Chu SH, Chang HJ | Factors Affecting the Quality of Life among the Patients with Pulmonary Artery Hypertension | 2017 |
| **336** | 4 | Maurer M, Caballero T, Bouillet L | Gender Analysis of Baseline Characteristics and Treatment Outcomes in Patients (Pts) with Hereditary Angioedema Type I/Ii: Findings from the Icatibant Outcomes Survey | 2017 |
| **337** | 4 | Jesenak M, Hrubiskova K, Hrubisko M, Vavrova L, Banovcin P | Hereditary Angioedema in Slovakia-Results from National Pilot Study | 2017 |
| **338** | 4 | Mechin H, Albrecht C, Siraeva AD, Vernet S,  Tissier JC, Thevenon J | Impact of Using an Electronic Platform for an Early-Access Program for Daratumumab in France: A System Utilisation and Satisfaction Study | 2017 |
| **339** | 4 | Hare H, Gagnon B, Kinel S, et al. | Industry Collaborations in Clinical Trial Development: Leveraging in-Person Patient and Caregiver Input in Huntington Disease | 2017 |
| **340** | 4 | Pina-Rivera Y, Godfrey RM, Sesay SO | Mccune-Albright Syndrome with Growth Hormone Deficiency-Case Report | 2017 |
| **341** | 4 | Krysanova V, Krysanov I, Ermakova V | The Multicriteria Decision Analysis of Using Tetrabenazine for Patients with Hungtington's Disease in Russia | 2017 |
| **342** | 4 | Kaussen T, Zellmer A, Käditzz H, Seidemann K, von Kries R, Sasse M, Beerbaum P | P065. Espedacs-Surveillance Study on Epidemiology, Diagnostics and Therapy of Abdominal Compartment Syndrom in Critical Ill Children in Germany: Preliminary Data | 2017 |
| **343** | 4 | Kamiya C | Peripartum Cardiomyopathy: How Can We Approach Such a Heterogeneous Disease? | 2017 |
| **344** | 4 | Pin A, Yuen C, Wong C | Poems Syndrome: Rare Disease That Is Usually Overlooked | 2017 |
| **345** | 4 | Palma JA, Porciuncula A, Krismer F, Meissner W, Kaufmann H, Norcliffe-Kaufmann L | Preliminary Results of the Global Multiple System Atrophy Registry (Glomsar): An Internet-Based Patient-Reported Registry | 2017 |
| **346** | 4 | Matsushima M, Sakushima K, Yabe I | Progress Report of the Registry Study for Multiple System Atrophy in Hokkaido, Japan: Horc-Msa Project 2014-17 | 2017 |
| **347** | 4 | Parthenaki L, Balvanyos J, Crowley G, Tomazos LC | Quality of Life in Adults with Hypophosphatasia: Results from a Multicountry Survey | 2017 |
| **348** | 4 | Matsushima M, Sakushima K, Yabe I | A Registry Study of Multiple System Atrophy in Hokkaido, Japan: Horc-Msa Project 2014-16 | 2017 |
| **349** | 4 | Gonano C, Pasquier J, Daccord C, Lazor R | Risk of Pneumothorax Due to Air Travel in Pulmonary Lymphangioleiomyomatosis | 2017 |
| **350** | 4 | Mol P, Vries S | Which Patients Are Interested in an App for Reporting Adverse Drug Reactions and Receiving Drug Safety Information? | 2017 |
| **351** | 4 | Dabaj I, Coqué N, Chemaly N, Nabbout R | Antiepileptic Treatment in Dravet Syndrome: An Additional Complexity for the Families | 2017 |
| **352** | 4 | Ferrada M, Sinaii N, Sikora KA | Children with Relapsing Polychondritis Are Likely to Be Seen in the Emergency Room Prior to Establishing the Diagnosis | 2017 |
| **353** | 4 | Kim C, Delaney K, McNamara M, Chia V, Romanov V | Cross-Sectional Physician Survey on the Use of Minimal Residual Disease (Mrd) Testing in the Management of Pediatric and Adult Patients with Acute Lymphoblastic Leukemia (All) | 2017 |
| **354** | 4 | Allen G, Hall A, Hanman K, Le Fevre R, Griffiths A | Do Eu5 Countries with Favourable Healthcare Expenditure and Reimbursement Indicators Have Better Patient-Reported Access to Treatments for Rare Diseases? | 2017 |
| **355** | 4 | Bertolin C, Querin G, Martinelli I, Meo G, Pegoraro E, Sorarù G | Epidemiological Survey of Sbma in Italian North-East Regions | 2017 |
| **356** | 4 | Dong D, Batista J, Belk K | Evolving Observational Registries: An Evidence Based Approach to Understanding Data Availability in the "Sanofi Genzyme Rare Disease Registries" | 2017 |
| **357** | 4 | Cozmuta R, Fraenkel L | Factors Impacting Patients' Decision to Start Treatment Vary by Race | 2017 |
| **358** | 4 | Gaudet D, Blom D, Bruckert E, Stroes E, Kastelein J, Kane J, et al. | Familial Chylomicronemia Syndrome (Fcs) Patients Recruited to the Approach Trial of Volanesorsen Therapy Are Representative of Subjects with Fcs | 2017 |
| **359** | 4 | Desyatik EA, Shadrivova OV, Borzova JV, Bogomolva TS, Mitrofanov Vs, Vasileva NV, et al. | First Case of Imported Pulmonary Coccidioidomycosis in Russia | 2017 |
| **360** | 4 | Fu L, Kanani A, Lacuesta G, Waserman S, Betschel S | An in Depth Look at the Medical Management of Hereditary Angioedema through a Canadian | 2017 |
| **361** | 4 | Brown C, Bodamer O, Levy H, Waisbren S | International Pku Patient Registry | 2017 |
| **362** | 4 | Craig M, Wiehn J, Descours A | Patient and Disease Registry Publications: An Online Survey of Medical Publication Professionals | 2017 |
| **363** | 4 | Durães J, Tábuas-Pereira M, Lacerda L, Alonso I, Do Carmo Macário M | Quality of Life in Adult Patients with Leukodystrophies: The Impact of Perceived Fatigue | 2017 |
| **364** | 4 | Bogart KR, Frandrup E, Locke T, Thompson H, Weber N, Yates J, et al. | “Rare Place Where I Feel Normal”: Perceptions of a Social Support Conference among Parents of and People with Moebius Syndrome | 2017 |
| **365** | 4 | Bodemer C, Paller A, Wisk J, Giuliano J, Cantor E, Lagast H | A Survey of Patient Registries in Epidermolysis Bullosa: A Call for Standardized Methodology and Increased Collaboration | 2017 |
| **366** | 4 | Ferrara L, Morando V, Tozzi V | The Transition of Patients with Rare Diseases between Providers: The Patient Journey from the Patient Perspective | 2017 |
| **367** | 4 | Dakhoul L, Fogel R, Vuppalanchi R, Lammert C | Unconventional Immunosuppressive Therapies for Treatment of Autoimmune Hepatitis and Finding the Current Trends and Patterns Using Social Media Approach | 2017 |
| **368** | 4 | White MK, Bayliss M, McCausland K, Oliver C, Guthrie SD | Changes in Health-Related Quality of Life (Hrqol) between an Initial and Six-Month Follow-up Survey in an Observational Community-Based Study of Patients with Al Amyloidosis | 2016 |
| **369** | 4 | Szentes B, Witt S, Bush A, Cunningham S, Emiraliouglu N, Goldbeck L, et al. | Current Practice of Drug Treatment in Children with Ild: First Insights from the Child-Eu Registry | 2016 |
| **370** | 4 | Wu X, Long E, Lin H, Liu Y | Global Prevalence and Epidemiological Characteristics of Congenital Cataract: A Systematic Review and Meta-Analysis | 2016 |
| **371** | 4 | Zanichelli A, Magerl M, Longhurst H, Aberer W | Improvement in Hereditary Angioedema Diagnosis: Findings from the Icatibant Outcome Survey | 2016 |
| **372** | 4 | Tulloh R, Ramanan A, Brogan P, Tizard J, Conolly GM, Harnden A, et al | Kawasaki Disease. Results of the Bpsu Survey in Uk and Ireland | 2016 |
| **373** | 4 | Sucipto, Estiasari R, Imran D | Survey of Multiple Sclerosis in Indonesia: Diagnostic Approach and Treatment Selection | 2016 |
| **374** | 4 | McCausland K, Bayliss M, White, MK Guthrie S | Burden of Disease and Clinical Characteristics of an Al Amyloidosis Disease Population with and without Cardiac Involvement | 2016 |
| **375** | 4 | Reed DA, Liao J, Stauffer C, Kornreich R, Yang AC | Clinical Diagnosis of Gaucher Disease Type 3 with a Rare Genotype | 2016 |
| **376** | 4 | Lazzarotto D, Candoni A, Fili C, Forghieri F, Pagano L, Busca A, et al. | Clinical Outcome and Effect of Allo-Sct in Adult Patients with De Novo or Aml-Related Myeloid Sarcoma. Results from an Italian Multicenter Survey | 2016 |
| **377** | 4 | Nagel s, Geisbuesch C, Busse O, Harmann G, Ringleb P | Diagnostic and Therapeutic Workup for Cerebralvenous Thrombosis-Results from a Survey on German Stroke Units | 2016 |
| **378** | 4 | Rahaghi F, Alnuaimat H, Awdish R, Balasubramanian V, Bourge R, Burger C, et al. | Expert Consensus on the Prescribing Practice and Management of Adverse Events Associated with the Treatment of Patients Taking Macitentan for Pah: A Delphi Consensus Study | 2016 |
| **379** | 4 | Liu G, Needleman K, Lewis D, Rao G. | Fda's New Natural History Grant Program: Support to Build a Solid Foundation for Development of Products for Rare Diseases | 2016 |
| **380** | 4 | McCormick N, Reimer K, Marra C, Sidi S, Smith SP, Avina-Zubieta A | Feasibility of an Approach to Contact Individuals from Administrative Health Data to Estimate the Economic Burden of Systemic Autoimmune Rheumatic Diseases at the General Population Level | 2016 |
| **381** | 4 | Silva-Paredes G, Cornejo-Olivas M, Inca-Martinez M, Espinoza-Huertas K, Vishnevetsky A, Mazzetti P, et al. | A Health Cost Analysis for Huntington Disease in Peru | 2016 |
| **382** | 4 | Longhurst H, Aberer W, Bouillet L, Cabllero T, Bygum A, Grumach AS, et al. | Hereditary Angioedema Presents in Childhood but Is Diagnosed in Adulthood - Findings from the Icatibant Outcome Survey | 2016 |
| **383** | 4 | Stumpo M, Guttmann S, Volpini M, Volpinin M, Monaldini C, Manzaroli D, et al | High Prevalence of Huntington's Disease in San Marino Republic: An Epidemiological Survey | 2016 |
| **384** | 4 | Maurer M, Caballero T, Aberer W, Zanichelli A, Bouillet L, Fabien V, et al. | The Icatibant Outcome Survey: More Than 2900 Icatibant-Treated Attacks in Patients with Type I or Ii Hereditary Angioedema | 2016 |
| **385** | 4 | Low R, Kay-Jones C, Dutt T. | The Impact of Ttp Specialist Centres on Patient Survival and Satisfaction | 2016 |
| **386** | 4 | Mossman J, Efthymiadou O, Kanavos P | An International Patient Survey on Health Related Quality of Life Aspects Not C Patured by Eq-5d-5l | 2016 |
| **387** | 4 | Huang B, Morgan E, Chen C, Adamas M, Sivaganisan S, Brunner H, et al. | Is Early Initiation of Biologic Treatment More Effective Than a Step up Treatment Approach for New Onset Juvenile Idiopathic Art Hritis?-Using a Causal Inference Approach to Achieve a Double Robust Estimate | 2016 |
| **388** | 4 | Requena MA, Dasí F, Barajas F, Castillo S, Benavent O, Escribano A | Knowledge of Alpha-1 Deficiency and Primary Ciliary Dyskinesia by Medical Students and Health Professionals | 2016 |
| **389** | 4 | Paoli X | An Online Survey of Neurologists About Charcot-Marie-Tooth Disease Type 1a | 2016 |
| **390** | 4 | Jain A, Mehta S, Joshi M, Garg K, Togal LK, Parnes A, et al. | Patient Reported Outcomes to Assess Quality of Hemophilia Care in North India-Results of a Global Partnership | 2016 |
| **391** | 4 | Nakajima T, Yoshifuji H, Terao C, et al. | Severe Complications and Immunosuppressive Treatments in 33 Patients with Relapsing Polychondritis | 2016 |
| **392** | 4 | Reyes M, Camps B, Knebelman N, de Solà-Morales O | Social Preferences for Rare Diseases in Spain | 2016 |
| **393** | 4 | Roman H | Surgical Management of Deep Colorectal Endometriosis in France: A Multicentric 1,135 Case-Series. On Behalf of the Friends Group (French Colorectal Infiltrating Endometriosis Study Group) | 2016 |
| **394** | 4 | Ogata K, Suzuki M, Yatabe K, Monna K, Tanaka Y, Nonaka I, et al | Survey on Usage of Telecommunication Terminals in Japanese Patients with Neuromuscular Diseases | 2016 |
| **395** | 4 | Philina Lee P, George TI, Shi H, Evans EK, Sing T, Boral AL, et al. | Systemic Mastocytosis Patient Experience from Mast Cell Connect, the First Patient-Reported Registry for Mastocytosis | 2016 |
| **396** | 4 | Simpson A, Hunter A, Whitaker MJ | Understanding the Wider Impact of Adrenal Insufficiency: Patient Organisation Involvement in the Tain Project | 2016 |
| **397** | 4 | Greywal T, Zaenglein AL, Baldwin HE, Bhatia N, Chernoff KA, Del Rosso JQ, et al. | Acne Fulminans and Its Variants: Evidence-Based Expert Panel Recommendations | 2016 |
| **398** | 4 | Gala S, Mwamburi M | Assessment and Characterization of Gaps in Literature of Resource Utilization and Economic Burden in Inflammatory Myopathies | 2016 |
| **399** | 4 | Cohen J, Chiang AL, Kumar NL, Horst D | Boston's Gastroenterology Interhospital Evening Rounds: A Novel Model for Academic Competitive Collaboration | 2016 |
| **400** | 4 | Dalin F, Nordling Eriksson G, Dahlqvist P, Hallgren Å, Wahlberg J, et al. | Clinical and Immunological Characteristics of Autoimmune Addison's Disease in Sweden: A Nationwide Multicenter Analysis of 660 Patients | 2016 |
| **401** | 4 | Park DS, Chang ™, Young IC, Koh YC, Kim HY, Roh HG | Diagnostic and Therapeutic Work-up for Cerebralvenous Thrombosis &Ndash; Results Froma Surveyongerman Stroke Units | 2016 |
| **402** | 4 | Efthymiadou O, Kanavos P1, Mossman J | Differentiation of Health Related Quality of Life Outcomes between Patient Populations; Results from Patients with Breast Cancer, Rheumatoid Arthritis, Multiple Sclerosis, Rare Cancers and Rare Diseases | 2016 |
| **403** | 4 | Goring SM, Risebrough N, Wilson JB, Watch J, Gallagher JR, Heao KJ, et al. | The Economic Burden of Non-Tuberculous Mycobacterial Pulmonary Disease in Canada, France, Unitedkingdom and Germany | 2016 |
| **404** | 4 | Benedikt H, Celina S, Cornelia B, Edda H, Peter W, Steffen B, et al. | Epilepsy and Its Treatment in Nicolaidesbaraitser Syndrome | 2016 |
| **405** | 4 | Fernandeza I, Ahmed SF, Bishop N, Brandi ML, Doulgeraki A, Haeusler G, et al. | A European Survey to Identify New Roads for Care, Training and Research around Rare Metabolic Bone Diseases | 2016 |
| **406** | 4 | Hammann MJA, Effing-Boele MC, Dekker HK | Expertise for Rare Diseases Mapped | 2016 |
| **407** | 4 | Jessop S, Crudgington D, London K, Kellie S, Howman-Giles R | Fdg-Pet/Ct for the Evaluation of Non-Osseous Sites of Langerhans Cell Histiocytosis | 2016 |
| **408** | 4 | Guthrie S, White MK, McCausland KL, Bayliss M. | The Impact of Al Amyloidosis on Absenteeism, Reduced Productivity, and Job Loss | 2016 |
| **409** | 4 | Dhonncha EN, Clowry J, Hennessy B, Stratton J, Field S | Langerhans-Cell Histiocytosis Presents as Vulval Disease in a 16-Year-Old Girl | 2016 |
| **410** | 4 | Oplatek A, Brown K, Sen S, Halerz M, Supple K, Gamelli RL | Long-Term Outcomes of Patients Treated with Toxic Epidermal Necrolysis | 2016 |
| **411** | 4 | Gonzalez A, Zurynski Y, Phu A, Deverell M, Elliott E | Paediatricians Caring for Children with Rare Diseases: Survey of Current Knowledge, Practice and Future Educational Needs | 2016 |
| **412** | 4 | de Sola-Morales O, Knebelman N | Population Preferences for Rare Diseases in Spain | 2016 |
| **413** | 4 | Hollin IL, Peay H, Bridges JF | Pulmonary Benefits of Treating Duchenne Muscular Dystrophy: A Regulatory Benefit-Risk Analysis | 2016 |
| **414** | 4 | Dulas F, Dávila Vanegas MM, Weber S. | The Rare Disease Joint Action and How to Make Rare Diseases Visible in Health Care Systems | 2016 |
| **415** | 4 | Bayliss M, White MK, McCausland KL, Guthrie S | The Relative Burden of Al Amyloidosis on Health-Related Quality of Life | 2016 |
| **416** | 4 | Bayliss M, White MK, McCausland KL, Guthrie S | Treatment History, Tolerability and Impact on Health-Related Quality of Life in Al Amyloidosis | 2016 |
| **417** | 4 | Bayliss M, White MK, McCausland KL, Oliver C, Guthrie SD | Treatment of Visual Impairment in Patients with Leber's Hereditary Optic Neuropathy (Lhon) Using Idebenone (Raxone®) | 2016 |
| **418** | 4 | Bayliss M, White MK, McCausland KL, Oliver C, Guthrie SD | Treatment-Related Symptoms and Impact on Health-Related Quality of Life in Al Amyloidosis | 2016 |
| **419** | 4 | Dwyer A, Quinton R, Morin D, Pitteloud N | Unheard Voices: The Experiences and Illness Perceptions of Women with Kallmann Syndrome | 2016 |
| **420** | 4 | Andrews W, Garceau D, Sablinski T, Zhang P, Waldman T | Aa Amyloidosis: An Evaluation of Epidemiology and Prevalence in the Us and Eu5 Countries | 2016 |
| **421** | 4 | Bayliss M, White MK, McCausland KL, Oliver C, Guthrie SD, Lo S, Sanchorawala V | Burden of Al Amyloidosis on Health-Related Quality of Life in Clinic-Based, Community-Based, and Trial-Based Studies | 2016 |
| **422** | 4 | Ariceta G, Camacho JA, Fernández-Obispo M, Fernandez-Polo A, Gamesz J, Garcia-Villoria J, et al. | Cystinosis in Adult and Adolescent Patients: Recommendations for the Comprehensive Care of the Disease in Spain | 2016 |
| **423** | 4 | Barra L, Borchin R, Burroughs C, McMahon T, Adagaria B, Goebel S, et al. | Impact of Vasculitis on Employment and Income: An Online Survey of Participants in the Rare Diseases Clinical Research Network-Vasculitis Clinical Research Consortium Patient Contact Registry | 2016 |
| **424** | 4 | Barohn R, Statland J, Kimminau K, McMahon T, Adagaria B, Goebel S, et al. | Patient Activity of Daily Living for Amyotrophic Lateral Sclerosis | 2016 |
| **425** | 4 | Amengual T, Adams H, Mink J, Augustine E | Rare Disease Clinical Research: Caregivers' Perspectives on Barriers and Solutions for Clinical Research Participation | 2016 |
| **426** | 4 | Tallar M, Chen W, Gallagher JL, Gedeit R, Zafra H | Compartment Syndrome Requiring Fasciotomy in a Patient with Hae Type 1: A Case Report | 2015 |
| **427** | 4 | Stacpoole PW | Development of an Observer-Reported Outcome Measure of Home Functionality in Children with Primary Mitochondrial Diseases | 2015 |
| **428** | 4 | Syed AR, Calles-Escandon J, Wolfe MM | Epidemiology of Gastroparesis with and without Diabetes Mellitus in a Large Cohort from 340 Us Hospitals | 2015 |
| **429** | 4 | Paz S, Torrent J, Poveda JL, et al | Experts Consensus on the Future of Rare Diseases Care and Orphan Drugs Access in Spain: A Delphi Study | 2015 |
| **430** | 4 | Weber TJ, Sawyer EK, Moseley S, OdrIjin T, Kishnani PS | Fracture and Surgical Burden in Pediatric and Adult Patients with Hypophosphatasia: Results from Patient Reported Outcome Surveys | 2015 |
| **431** | 4 | Wong K, Forrest C, Schunemann H | Generating Evidence in Cleft Care: A Delphi-Like Study to Identify and Address Barriers to Carrying out Randomized Controlled Trials | 2015 |
| **432** | 4 | Travlos V, Downs J, Wilson A, Patman S | How Is Engagement in Health Care More Than Adherence or Participation? Supporting Adolescents Living with Neuromuscular and Rare Disease | 2015 |
| **433** | 4 | Sweidan AJ, Cantillep A, Sohn D, Burg A | Idiopathic Hypereosinophilia with Warm Autoimmune Hemolytic Anemia | 2015 |
| **434** | 4 | Xu L | Mccune-Albright Syndrome, a Rare Occurrence | 2015 |
| **435** | 4 | Marika PM, Katja P, Marja A, Katri HA | Medicines Information Needs among Medicine Users in Finland-a Population Survey | 2015 |
| **436** | 4 | Zifodya JS, Kim HP, Silver HJ, Slaughter JC, Higginbotham T, Vaezi MF | Nutritional Status of Patients with Untreated Achalasia | 2015 |
| **437** | 4 | Perrone ME, Carmody D, Philipson LH, Greeley SA | An Online Discussion Group for Rare Monogenic Diabetes Patients: Supporting Families and Fueling New Research | 2015 |
| **438** | 4 | Sato K, Kikuchi T, Kimura M, Komita M, Shimada K, Seki K, et al. | Patient Satisfaction Survey for Ham-Net Registrants | 2015 |
| **439** | 4 | Sultana R | Prevalence of Sleep Problems in Children with Neurodevelopmental Disorders: A Follow-up Study | 2015 |
| **440** | 4 | Sun Y, Wang D, Salvadore G, Singh J, Casper C, Van Rhee, et al. | Siltuximab Improves Depressive Symptoms in a Randomised, Placebo-Controlled, Phase 2 Study in Patients with Multicentric Castlemen's Disease | 2015 |
| **441** | 4 | Palkmets O, Balvanyos J, Parthenaki I, Alnwick-Allu K, Hutchings A | Societal and Non-Healthcare Costs Associated with Atypical Haemolytic Uraemic Syndrome in the Uk: Results of a Patient Survey | 2015 |
| **442** | 4 | Picanco Jr OM, Picanco TSC, Alves RMS, Soares PC | Vermiform Appendix Carcinoma: Clinical Presentation, Diagnosis and Treatment | 2015 |
| **443** | 4 | Chung JH | Asia | 2015 |
| **444** | 4 | Gallagher JR, Obradovic M, Risebrough N, Heap K, Watch J | A Cost-Effective Enhanced Retrospective Observational Study Methodology to Capture Economic Burden Evidence in a Rare Disease Using Non-Tuberculous Mycobacteria Infection as a Model | 2015 |
| **445** | 4 | Brook RA, Smeeding JE, Sax MJ | The Current Landscape and Expected Changes in Formulary Management | 2015 |
| **446** | 4 | Marshall DA, MacDonald KV, Lopatina E, Mackenzie A, Hartley T, Boycott K | Estimating the Value of Whole Exome Sequencing for Parents of Children with Rare Genetic Diseases | 2015 |
| **447** | 4 | Malinowski, Alexandra, Utz, Zanrucha B, Martin D | Evaluation of United States Schools and Colleges of Pharmacy Curriculum to Assess Education on Lysosomal Diseases | 2015 |
| **448** | 4 | Elsevier BV | Gaucher Disease: Lived Experience of Patients in the Context of a French National Patient Therapeutic Education Program | 2015 |
| **449** | 4 | Liu W, Meng Z, Liu H, LiW, Wu Q, Cy E, et al. | Hepatic Epithelioid Angiomyolipoma: A Rare and Potentially Severe Treatble Tumor Accompneny with 3 Cases Report | 2015 |
| **450** | 4 | Lloyd A, Gallop K, Hutchings A, Acaster S | How Do We Estimate Quality Adjusted Life Years (Qalys) in Rare Diseases? A Case Study in Hypophosphatasia | 2015 |
| **451** | 4 | Mooney J, Watts RA, Scott DGI | How Long Does It Take to Get Diagnosed with Anca Vasculitis? | 2015 |
| **452** | 4 | Colombatti R, Palazzi G, Masera N, Notarangelo LD, Bonetti E, Samperi P, et al. | Hydroxiurea Prescription, Availability and Use for Children with Sickle Cell Disease in Italy: Results of the Italian Association of Pediatric Hematology Oncology (Aieop) Multicenter Survey | 2015 |
| **453** | 4 | Maurer M, Caballero T, Aberer W, Zanichelli A, Bouillet L, Fabien V, et al. | The Icatibant Outcome Survey: More Than 1500 Icatibant-Treated Attacks in Patients with Type I or Ii Hereditary Angioedema | 2015 |
| **454** | 4 | Longhurst HJ, Aberer W, Bouillet L, Zanichelli A, Bouillet L, Fabien V, et al. | The Icatibant Outcome Survey: More Than 2200 Icatibant-Treated Attacks in Patients with Type I or Ii Hereditary Angioedema | 2015 |
| **455** | 4 | Wu SL, Bai JG, Xu J, Ma QY, Wu Z | Necrolytic Migratory Erythema as the First Presentation of Pancreatic Glucagonoma | 2015 |
| **456** | 4 | Chow W, Ing A, Rowe D | Non-Invasive Ventilation in Motor Neuron Disease/Amyotrophic Lateral Sclerosis: An Australasian Perspective | 2015 |
| **457** | 4 | Isabelle L, Raymond C, Heather L, Spencer G, Giampaolo M | Patient Experience with Hereditary and Senile Systemic Amyloidoses: A Survey from the Amyloidosis Research Consortium | 2015 |
| **458** | 4 | Ashley Farmer, Kelly Colvin, Tippu Khan, Kam T, Mason S, Davis C, et al. | Preparation of the New Graduate Rn for Care of the Hematopoietic Cell Transplant Patient: An Orientation Reconstruction Story | 2015 |
| **459** | 4 | Manno M, Golfieri L, Lauro A, Zanfi C, Venturoli A, Tossani E, et al. | Psychological and Physical Health in Patients with Cipo: Transplant and Non-Transplant Surgery | 2015 |
| **460** | 4 | Lapointe anne-sophie, Moutel G, Gimenes P | Rare Diseases and Patient Organization Collaboration in the Medical Research: Analysis of the Issues with All the Protagonists | 2015 |
| **461** | 4 | Kaushik P, Bhalla S, Jain A | Reporting Instruments of Patient Reported Outcomes in Orphan Disease | 2015 |
| **462** | 4 | Kaura S, Seetasith A, Ramesh V, Nanavaty M, Proach J, Khan Z.M | A Survey of Ispor Attendee Views on the Use of Icer and Acceptable Icer Thresholds | 2015 |
| **463** | 4 | Chegini Z, Maghari A, Ebrahimi A, Seraji B, Khorshidfar M | Therapeutic Apheresis and Its Indications: Referred to Tehran Blood Transfusion Centre | 2015 |
| **464** | 4 | Guthrie S, Chapman D, Koller M, Jackie Walling J, Kinney GG | Who Is Treating Al Amyloidosis and How Are They Doing It? Results of a Global Physician Survey | 2015 |
| **465** | 4 | Zanichelli A, Arcoleo F, Barca MP, Borrelli P, Bova M, Cancian M, et al. | A Nationwide Survey of Hereditary Angioedema Due to C1 Inhibitor Deficiency in Italy | 2014 |
| **466** | 4 | Meunier B, Harle J, Chekroun M, Chiche L | Characteristics, Needs and Sources of Information of a French Online Community of Systemic Lupus Erythematosus Patients | 2014 |
| **467** | 4 | Yagi H, Hayakawa M, Yamaguchi N, Yamashita K, Taniguchi S, Matsumoto M, et al. | Decreased Platelet Thrombus Size, Due to a Heightened Proteolysis of Vwf by Adamts13, Is Quickly Restored after Valve Replacement in Aortic Stenosis Patients | 2014 |
| **468** | 4 | Benjamin Xu, Paul Israelson, Jessy Dana Dorn, Mark S Humayun, Lisa C Olmos | Developing an Improved Method to Evaluate Retinitis Pigmentosa Patients for the Argus Ii Retinal Prosthesis System | 2014 |
| **469** | 4 | Kumar A, Reddy A, Elliott W | Elderly Female with Anemia and Acute Renal Failure | 2014 |
| **470** | 4 | Sura M, Gerasimova K, Omelyanovsky VV, Avxentyeva M, Tatarinov A, Fedyaev D | Estimating the Costs of Drug Supply for Rare Diseases Patients in Russia | 2014 |
| **471** | 4 | Perryman S, Sreepati G, Chalasani N, Lammert C | Fatigue and Itch Are Associated with Less Biochemical Response in Treated Autoimmune Hepatitis | 2014 |
| **472** | 4 | Péntek M, Baji P, Pogány G, Brodszky V, Boncz I, Gulácsi L | Health Related Quality of Life of Patients and Their Caregivers in Rare Diseases Results of the Burqol-Rd Project in Hungary | 2014 |
| **473** | 4 | Rizzardo S., Bansback N., Mitton C., Marra C., Lynd L. | How Canadians Value Rare Diseases Given Their Opportunity Cost? | 2014 |
| **474** | 4 | Naruse S, Ishiguro H, Yamamoto A, Kondo S, Nakakuku M, Hoshino M, et al. | Incidence and Exocrine Pancreatic Function of Cystic Fibrosis in Japan | 2014 |
| **475** | 4 | Small M, Piercy J, Pike J, Cerulli A. | Incremental Burden of Disease in Patients Diagnosed with Pulmonary Arterial Hypertension Receiving Monotherapy and Combination Vasodilator Therapy | 2014 |
| **476** | 4 | Trip AM, Tsiachristas A, Koenders JM, Kanters TA | Multi-Criteria Decision Analysis for Reimbursing Orphan Drugs: A Dutch Demonstration Study Using the Analytic Hierarchy Process Method | 2014 |
| **477** | 4 | Takeuchi F, Nakamura H, Mitsuhashi S, mori-Yoshimura M, Hayashi YK, et al. | National Registry of Japanese Dystrophinopathy Patients: Remudy | 2014 |
| **478** | 4 | Peay HL, Scharff H, Biesecker BB | Perceived Barriers and Facilitators of Participation in Clinical Trials for Duchenne and Becker Muscular Dystrophy | 2014 |
| **479** | 4 | O'Mahony B, Kent A, Aymé S | Pfizer-Sponsored Satellite Symposium at the European Haemophilia Consortium (Ehc) Congress: Changing the Policy Landscape: Haemophilia Patient Involvement in Healthcare Decision-Making | 2014 |
| **480** | 4 | Opotowsky AR, Moko L, Kim Y, et al | Pheochromocytoma-Paraganglioma Syndromes and Cyanotic Congenital Heart Disease: An International Multicenter Case Series and Population-Based Epidemiologic Study | 2014 |
| **481** | 4 | Meijden JC, Gungor D, Kruijshaar ME, Ans T | The Pompe Survey: A Summary of 10 Years Follow-up through Patient Reported Outcomes on Non-Classic Pompe Patients | 2014 |
| **482** | 4 | Stourac P, Bednarova J, Praksova P, Hladikova M, Kantorova K | The Population-Based Survey of Paraneoplastic Neurological Syndromes in the Czech Republic During the Eight Years' Period | 2014 |
| **483** | 4 | Wiss J, Levin LA | Preferences for Prioritizing Patients with Rare Diseases: A Survey of the General Population in Sweden | 2014 |
| **484** | 4 | Ponet O, Tasdemir E, Magestro M, Griner BP, Gummins G, Van Engen A, et al. | Prevalence-Based Measurement of the Economic Burden of Rare Diseases: Case Review to Determine the Annual Cost of Acromegaly in Italy | 2014 |
| **485** | 4 | Vastert S, Dolezalova P, Feldman B, Ravelli A, Wulffraat N, Martini A, *et al.* | Share-Workpackage 5: Development of Best Practices of Diagnosis and Treatment for Paediatric Rheumatic Diseases Throughout Europe | 2014 |
| **486** | 4 | Sharp CA, Tansley S, New P, Richard E, Rober G, Cooper NM, et al. | Specialist Commissioning: Does Treatment by Experts Influence Outcomes for Patients with Rare Diseases? An Exemplar from Myositis | 2014 |
| **487** | 4 | Pieri L, Bonadonna P, Elena C, Papayannidis C, Grifoni FI, Rondoni M, et al. | A Survey on Clinical and Biological Characteristic and Therapy Management of an Italian Series of 455 Adult Patients with Systemic Mastocytosis on Behalf of Italian Registry of Mastocytosis | 2014 |
| **488** | 4 | Weiglein T, Vehling-Kaiser U, Kaiser F | Transdifferentiation from Chronic Lymphocytic Leukemia into Langerhans-Cell-Sarkoma: A Case Report | 2014 |
| **489** | 4 | Coi A, Santoro M, Lipucci M | Characterization and Classification of Rare Disease Registries by Using Exploratory Data Analyses | 2014 |
| **490** | 4 | Fabriani V, Marcellusi A, Mennini FS, Giannantoni P | Cost of Illness Analysis of Duchenne Muscular Dystrophy in Italy | 2014 |
| **491** | 4 | Tailor V, Ridder L | Developing a European Clinical Research Network for Paediatric Gastroenterology , Hepatology and Nutrition | 2014 |
| **492** | 4 | Karakina M, Tuzankina I | The Early Recognition Program of Primary Immunodeficiencies in the Ural Region | 2014 |
| **493** | 4 | Iskrov G, Houÿez F | European Network of Rare Disease Help Lines (Enrdhls)-Caller Profile Analysis 2013 | 2014 |
| **494** | 4 | Houÿez F, Tessier L, Synodinos D | Health Care Cost-Containment Measures in the Context of the Economic Crisis: Impact Analysis | 2014 |
| **495** | 4 | Caballero T, Caminoa MJ, Pérez-Fernández E, et al. | Health-Related Quality of Life (Hrqol) in Adult Patients with Hereditary Angioedema Due to C1 Inhibitor Deficiency (Haec1-Inh) Assessed by Sf-36v2 | 2014 |
| **496** | 4 | Dan D, Castro R | Identifying Specific Social Challenges of Rare Diseases: Current Challenges and Issues | 2014 |
| **497** | 4 | Hanson KA, Dudka P, Payne KA, Beatty M, Frazee SG | Novel Approaches to Patient Recruitment and Data Integration | 2014 |
| **498** | 4 | Joshi Namita Khanna R, Holmes E | Pharmacists' Awareness and Knowledge of Multiple Sclerosis | 2014 |
| **499** | 4 | Jayasena A, Muscarella M, Ahmed F | Rare Disease Registries: Perception of Parents and Young People | 2014 |
| **500** | 4 | Han C, Kavan augh A, Genovese MC, Deodhar A, Hsu B, Hsia E | Sustained Improvement in Health-Related Quality of Life, Work Productivity, Employability and Reduced Healthcare Resource Utilization of Patients with Rheumatoid Arthritis, Psoriatic Arthritis and Ankylosing Spondylitis Treated with Golimumab: 5-Year Results from Three Phase Iii Studies | 2014 |
| **501** | 4 | Rizzardo S, Lynd L | Canadian Values, Attitudes and Willingness to Pay for Expensive Medications for Rare Diseases | 2013 |
| **502** | 4 | Witt M, Meier J, Proft F, Schulze-koops H, Grunke M | Characterizing Disease Burden in an Ultra-Rare Disease in the United States: Transthyretin (Ttr) Amyloidosis Patients & Caregivers | 2013 |
| **503** | 4 | Ventura P, Marchini S, Rocchi E, et al. | Clinical Management of Porphyrias in Italy: The Grip Is Born | 2013 |
| **504** | 4 | Witt M, Meier J, Proft F, Schulze-Koops H, Grunke M | Diagnosis, Disease Burden and Disease Manifestations of the Sapho Syndrome in Germany: Results from a Nationwide Patient Survey | 2013 |
| **505** | 4 | Plebani A, Quinti I, Martire B, et al. | Improving the Awareness of Pids among Physicians: The Experience of the Italian Registry (Ipinet) | 2013 |
| **506** | 4 | Yang G, Tuomari A | An in-Depth Analysis of the Prevalence and Socio-Demographic Factors Associated with Lupus Nephritis in Major Industrialized Countries | 2013 |
| **507** | 4 | Rasheed A, Vasudevan V, Abbas Q | Life Threating Hyponatremia as a Presenting Manifestation of Renal Amyloidosis | 2013 |
| **508** | 4 | Turpeinen R, Kalam-Salminen L, Lampi H, Kaivos S, Kyngas H, Kaariainen M | Quality of Counselling of Patients with Cardiac Sarcoidosis | 2013 |
| **509** | 4 | Pearce KF, Hildebrandt M, Greinix H, et al. | The Regulation of Advanced Therapy Medicinal Products in Europe and the Role of Academia | 2013 |
| **510** | 4 | Partida-Gaytan A, Yamazaki-Nakashimada MA, Berron-Ruiz L, López-Herrera G | Selective Igm-Deficiency-to Be or Not to Be | 2013 |
| **511** | 4 | Pinion AK, Gierer S | The Six Year Dermatosis: Chronic Urticaria Due to Schnitzler Syndrome | 2013 |
| **512** | 4 | Bourguignon S, Reznik Y, Chenuc G | Adrenal Insufficiency: Burden of Disease in France in 2011 | 2013 |
| **513** | 4 | Hicks N, Toumi M | An Approach for Quantification of Patient Advocacy Group Input in the Hta Process | 2013 |
| **514** | 4 | Donner EJ, Jeffs T, Jette N | Are Canadian Pediatricians Aware of Sudep? | 2013 |
| **515** | 4 | García ML, Blanco JM, Gómez C, et al | Bone Involvement in Systemic Mastocytosis: Cases Review | 2013 |
| **516** | 4 | Mansouritorghabeh H, Banihashem A, Modaresi A, Manavifar L | Circumcision in Males with Bleeding Disorders | 2013 |
| **517** | 4 | Di Gennaro L, Cravino M, Martinelli A, Racano C, Donzelli O | Congenital Pseudarthrosis of the Clavicle: Report of 21 Cases | 2013 |
| **518** | 4 | Ghosh K | Developing Haemophilia Care in India | 2013 |
| **519** | 4 | Fraenkel L, Wilhelms EA, Reyna VF | Do Numbers Make a Difference? | 2013 |
| **520** | 4 | Haydeh H | Evaluation of Risk Factors in Children with Acute Lymphoblastic Leukemia in Mashhad-Iran | 2013 |
| **521** | 4 | Grider T, Cuthbertson D, Feely S, Shy M | Health-Related Quality of Life by Sf-36 in Patients with Hnpp Compared to Those with Cmt1a | 2013 |
| **522** | 4 | Mihai R, Cutter D, Pezella F, Ramasamy K | Isolated Thyroid Plasmacytoma-a Very Rare Disease | 2013 |
| **523** | 4 | Motta I, Filocamo M, Poggiali E, Poggiali E, Dragani A, Domenica M, et al. | A Multicenter Observational Study for Early Diagnosis of Gaucher Disease in Patients with Splenomegaly and/or Thrombocytopenia | 2013 |
| **524** | 4 | Fisher CG, Goldschlager T, Boriani S, Varga PP, Hehiling M, Bilsky M, et al. | A Novel Scientific Model for Rare and Often Neglected Neoplastic Conditions | 2013 |
| **525** | 4 | Henrard S, Speybroeck N, Hermans C | Participation for Innovation: Survey of Motivation for Clinical Studies Evaluating New Treatments for Haemophilia | 2013 |
| **526** | 4 | Ziegler LE | Patient Education, a Full Component of Patient Care in France : Impact of the New Regulations-Results of a National Survey | 2013 |
| **527** | 4 | Bacon CJ, Hall CA, Shy ME | The Rare Diseases Clinical Research Network Contact Registry for the Inherited Neuropathies Consortium | 2013 |
| **528** | 4 | DiBenedetti D, Coles T, Sharma T | Recruiting Patients with a Rare Blood Disorder and Their Caregivers through Social Media | 2013 |
| **529** | 4 | Hildebrandt M, Apperley J, Dickinson Am, et al. | The Regulation of Advanced Therapy Medicinal Products (Atmps) in Europe and the Role of Academia: Results of an Fp7-Funded Research Project | 2013 |
| **530** | 4 | Lam NS, Thanh TD | Study of Some Factors Related to Epidemiology in Malignant Pleural Mesothelioma | 2013 |
| **531** | 4 | Giugliani R, Jesuino K, Brites A, Burin MG, Matte Y, Leistner SS, et al. | Survey of the First 1000 Patients Identified by the Mps Brazil Network | 2013 |
| **532** | 4 | Han C, Kavanaugh A, Genovese MC, Deohar A, Hsu B, Hsia E | Sustained Improvement in Health Related Quality of Life, Work Productivity, Employability, and Reduced Healthcare Resource Utilization of Patients with Rheumatoid Arthritis, Psoriatic Arthritis and Ankylosing Spondylitis Treated with Golimumab: 5yr Results | 2013 |
| **533** | 4 | Han C, Kavanaugh A, Genovese MC, Deodhar A, Hsu B, Hsai E | Sustained Improvement in Health-Related Quality of Life, Work Productivity, Employability, and Reduced Healthcare Resource Utilization of Patients with Rheumatoid Arthritis, Psoriatic Arthritis and Ankylosing Spondylitis Treated with Golimumab: 5-Year Results from 3 Phase Iii Studies | 2013 |
| **534** | 4 | Mohamed S, Al-Juryyan N, Hasanato R, Babiker A, Al-Otibi H, Al-Hakamy A, et al. | An Unusual Presentation of Phex Mutated X-Linked Hypophosphatemic Rickets in a Child | 2013 |
| **535** | 4 | Aygören-Pürsün E, Bygum A, Caballero T, Beusterien K, Hautamaki E, Sisic Z, et al. | Hereditary C1-Inhibitor Deficiency: Economic and Social Impact on Individuals and Their Families-Results of a Cross-Sectional Survey in Germany, Denmark and Spain-the Hae-Bois Study | 2013 |
| **536** | 4 | Rodger S, Antonova V, Brabec P, Catlin N, Garami M, Gramsch K, et al. | Care-Nmd: The Role of Patient Registries in an International Study of Care in Duchenne Muscular Dystrophy | 2012 |
| **537** | 4 | Houÿez F, Bélorgey C, North A, Czondi E, Lipucci di Paola M. | Compassionate Use Programmes for Rare Diseases: Proposals for Actions | 2012 |
| **538** | 4 | Derham C, Anderson I, Wilson J, Goodden J, Chumas P, Tyagi A | The Conundrum of Paediatric Lenticulostriate Artery Aneurysm: A Report of 2 Cases | 2012 |
| **539** | 4 | Kim J, Yoo K, Park Y, Park S, Hwang T, Choi Y, et al. | The Current Status of Hemophilia in Korea: Results from a Nationwide Survey | 2012 |
| **540** | 4 | Peyvandi F | Epidemiology and Management of Rare Bleeding Disorders | 2012 |
| **541** | 4 | Underbjerg L, Sikjaer T, Mosekilde L, Rejnmark L | The Epidemiology of Hypo-and Pseudo Hypo Parathy Roidism in Denmark | 2012 |
| **542** | 4 | Moulard O, Mehta J, Fryzek J, Olivares R, Iqbal U, Mesa RA. | Epidemiology of Myelofibrosis (Mf), Polycythemia Vera (Pv) and Essential Thrombocythemia (Et) in the European Union (Eu) | 2012 |
| **543** | 4 | Taruscio D, Gainotti S, Vittozzi L, Bianchi F, Ensini M, Posada M | Epirare Survey on Activities and Needs of Rare Disease Registries in the European Union | 2012 |
| **544** | 4 | Ruano E, Woimant F, Trocello JM | A European Patient Survey in Wilson Disease to Improve the Management of This Rare Disease | 2012 |
| **545** | 4 | Kirschner J, Rodger S, Vry J, Gramsch K, Lochmüller H, Bushby K | How Reference Networks Develop, Implement, and Monitor Guidelines | 2012 |
| **546** | 4 | Woehrer A, Slavc I, Stefanits H, Waldhoer T, Heinzl H, Zielonke N, et al. | Incidence of Embryonal Tumor with Abundant Neuropil and True Rosettes in Children: A Population-Based Study by the Austrian Brain Tumor Registry | 2012 |
| **547** | 4 | Mavris M, Le Cam Y | Involvement of Patient Organisations in Research and Development of Orphan Drugs for Rare Diseases in Europe | 2012 |
| **548** | 4 | Derham C, Goodden JR, Chumas P, Goddard T, Tyagi A | Lenticulostriate Artery Aneurysms in the Paediatric Population: A Treatment Conundrum | 2012 |
| **549** | 4 | Goto M, Okada M, Komaki H, Komaki H, Sugai K, Sasaki M, et al. | A Nation-Wide Survey for Marinesco-Sjögren Syndrome | 2012 |
| **550** | 4 | Dufour C, Pillon M, Oneto R, Oneto R, Peters C, Granceschetto G, et al. | Outcome of Aplastic Anemia in Children. A Survey on Behalf of the Saa and Pdwp of the Ebmt | 2012 |
| **551** | 4 | Hal Al, Rode M, Pagnoux C, Pagnoux C, Yachyshyn E | Patient-Driven on-Line Survey on Granulomatosis with Polyangiitis | 2012 |
| **552** | 4 | Urbinati D, Dorey J, Trigo P, Yebenes J, Toumi M | Quality of Life of Spanish Family Carers in Huntington's Disease | 2012 |
| **553** | 4 | Pierri P | A Route Map for the Patients Journey | 2012 |
| **554** | 4 | Linertova R, Lopez-Bastida J, Serrano-Aguilar P, Posada-de-la-paz M | Social Economic Burden and Health-Related Quality of Life in Patients with Rare Diseases in Europe (Burqol-Rd Project). Spanish Results | 2012 |
| **555** | 4 | Weintraub D, Duda JE, Carlson K, Luo P, Sagher O, Weaver F, et al. | Suicide Ideation and Behaviors after Deep Brain Stimulation for Parkinson's Disease: Results from a Randomized Controlled Trial | 2012 |
| **556** | 4 | Essig S, Gianinazzi ME, Xavier von der Weid N, Michel G, Kuehni C | Therapy-Induced Late Toxicity in Children with Rare Diseases: Long-Term Follow-up Is Needed but Gets Disrupted in Adolescence-a Study in Pediatric Oncology from the Swiss Childhood Cancer Registry | 2012 |
| **557** | 4 | Trocello JM, Ruano E, Woimant F | Wilson's Disease: A European Survey to Improve the Management of This Rare Disease | 2012 |
| **558** | 4 | Cassiman JJ | Can the Cross-Borders Directive Improve the Quality of Genetic Testing in the Future? | 2012 |
| **559** | 4 | Delcroix M, Adir Ym Andreassen A, Boonstra A, Ekmehag B, Escribano P, et al. | Care Organisation for Pulmonary Arterial Hypertension in Developed Countries: A Survey | 2012 |
| **560** | 4 | Chai G, Kaw G, Chuah K, Leong K, Chee BE | Diffuse Reticulonodular Shadows: A Rare Manisfestation of a Rare Disease | 2012 |
| **561** | 4 | Austin S, Thompson L, Conett G, McGinnity T | Family Experience of Childhood Obliterative Bronchiolitis-Findings from a Uk and Ireland National Survey | 2012 |
| **562** | 4 | Barnholt KE, Do CB, Kiefer AK, Nelson M, Harber JE, Robson ME, et al. | Genome-Wide Analysis and Characterization of an Online Sarcoma Cohort | 2012 |
| **563** | 4 | Arnason JE, Mendez LM | Induction Therapy for Gamma-Heavy Chain Disease with Bortezomib and Dexamethasone: A Case Report | 2012 |
| **564** | 4 | Cottin V, Cadranel J, Crestani B, Dalphin JC, Delaval Pm Israel-Biet D, et al. | Management of Idiopathic Pulmonary Fibrosis in France: A Survey of 1,456 Pulmonologists | 2012 |
| **565** | 4 | Bailile S, Midelet A, Gallet C, Trossaert M, Ternisien C, Itzhard N, et al. | Patients' Opinion Surveys Are Necessary to Assess the Quality of the Organization of a Health Facility | 2012 |
| **566** | 4 | Arnaud L, Devilliers H, Peng SL, Amoura Z | The Relapsing Polychondritis Disease Activity Index: International Development and Initial Validation of the First Disease Activity Score for Relapsing Polychondritis | 2012 |
| **567** | 4 | Huei-Chun Lin M, Pitukcheewanont P | A Case of I-Cell Disease (Mucolipidosis Type ii) Masquerading as Rickets | 2011 |
| **568** | 4 | Mahe JY, Kieny P, Tich SNT, Magot A, Durigneux J, Deries X, et al. | Development, Activities and Orientations of Multidisciplinary Consultations for Neuromuscular Diseases of the Nantes-Angers Reference Center for Rare Diseases | 2011 |
| **569** | 4 | Korthof ET, Van der Zalm J, Raphael MF, Bresters D, Tolar J, Alijurf M, et al | Dyskeratosis Congenita: Evaluation of Immune Status and Hematopoietic Stem Cell Transplantation. A Literature and Ebmt Data Base Survey of 75 Patients | 2011 |
| **570** | 4 | Scotti N, Petratoli A, Carlomagno R, Orlando F, Forni C, Alessio M | Hereditary Angioedema. A Condition to Remember | 2011 |
| **571** | 4 | Ndri H, Husson MC, Trouvin JH | Injectable Sodium Benzoate: Uses in France and Future Prospects | 2011 |
| **572** | 4 | Harms L, Sieb JP, Williams AE, Graham R, Shlaen R, Claus V, Pfiffner C | The Long-Term Disease History of Lems Patients and Current Treatment Patterns: Results of a Patient Interview Survey in Germany | 2011 |
| **573** | 4 | Utz J, Hazaert JJ, Whitley CH | Medication Therapy Management, a New Clinical Practice for Inherited Metabolic Disorders | 2011 |
| **574** | 4 | Kamiya C, Katsuragi S, Neki R, Yamanaka K, Yoshihito S, Keiko U, et al. | Nationwide Survey of Peripertum Cardiomyopathy in Japan | 2011 |
| **575** | 4 | Mirabel M, Luyt CE, Leprince P, Leprince P, Trouillet J-L, Leger P, et al. | Outcomes, Long-Term Quality of Life, and Psychologic Assessment of Fulminant Myocarditis Patients Rescued by Mechanical Circulatory Support | 2011 |
| **576** | 4 | Toplak N, Dolezalovà P, Constantin T, Sediva A, Pasic S, Ciznar P, et al. | Periodic Fever Syndromes in Eastern and Central European Countries: Results of a Multinational Survey | 2011 |
| **577** | 4 | Kamiya C | Peripartum Cardiomyopathy in Japan-Results from the Nationwide Survey | 2011 |
| **578** | 4 | Masszi T | Prevalence of Hairy Cell Leukemia in Hungary with Special Emphasis on the Treatment Habits of Hungarian Hematologists | 2011 |
| **579** | 4 | Henschke C | Provision and Financing of Medical Aids in the Management of Rare Diseases: The Case of Amyotrophic Lateral Sclerosis (Als) | 2011 |
| **580** | 4 | Lombard M, Manunta A, Brissot R, et al. | Reference Center Spina Bifida | 2011 |
| **581** | 4 | Desjardins C, Calvez S, Richard A, De Lonlay P, Valayannopoulos V, Brasseur Y, et al. | Acute Maple Syrup Urinary Disease Therapeutic Strategy in French Hospitals | 2011 |
| **582** | 4 | Bernal W, Ilan Y, Laterre PF, et al. | Clinical Management of Acute Liver Failure 2010: Results of a Pan-European Survey | 2011 |
| **583** | 4 | Fehr A, Theuermann P, Razum O | Delphi Expert Survey on Promoting Research and Development into Drugs for Neglected Diseases | 2011 |
| **584** | 4 | n/a | Oral Presentation...Conference of the World Society of Arrhythmias, in Athens, Greece, 11–14 December 2011 | 2011 |
| **585** | 4 | n/a | Poster Presentations...Conference of the World Society of Arrhythmias, in Athens, Greece, 11–14 December 2011 | 2011 |
| **586** | 4 | Eriksson MK, Hagberg L, Lindholm L, Malmgren-Olsson EB, Osterlind J, Eliasson M. | Quality of Life and Cost-Effectiveness of a 3-Year Trial of Lifestyle Intervention in Primary Health Care | 2011 |
| **587** | 4 | Campoeresi S, Sullian R, Prtichard-Jones K, Vassal G, Ladenstein R, et al. | The State of Research into Children with Cancer across Europe: Results of Key Opinion Leaders Survey and Innovative Policy Strategies for the Next Decade | 2011 |
| **588** | 4 | Grosse s | Using Administrative Data Sources for Population Surveillance for Hht | 2011 |
| **589** | 4 | Lee SE, Jang JY, Kim SW | Clinical Features and Treatment Outcome of Choledochal Cyst in Korea: A Nationwide Multicenter Survey | 2010 |
| **590** | 4 | Vehreschild JJ, Ruping MJGI  Farowski F, Cornely O | Clinicalsurveys.Net: A Web-Based Research Portal for Rare Diseases | 2010 |
| **591** | 4 | Tanaka J, Moriyama H, Takada T | Clinicopathological Features of Hard Metal Lung Disease | 2010 |
| **592** | 4 | Gizycki RV | Contribution of Rare Disease Patient Organisations to Medical Education | 2010 |
| **593** | 4 | Ohba R, Furuyama K, Tsuchiya S | Epidemiological and Genetic Analysis of Sideroblastic Anemia - Multicenter Study in Japan | 2010 |
| **594** | 4 | Nouri A, Ghorshi MA, Nouri P | Extra Medulary Plasmacytoma of the Urethra | 2010 |
| **595** | 4 | Hassan B, Popoola A, Olokoba A, Salawu FK | A Hospital Survey of Neonatal Tetanus at a District General Hospital in Northeast Nigeria | 2010 |
| **596** | 4 | Kairemo E, Jarva H, Meri S | A Nationwide Survey of Paroxysmal Nocturnal Haemoglobinuria in Finland | 2010 |
| **597** | 4 | Sung N | Philanthropic Strategies to Accelerate Drug Development | 2010 |
| **598** | 4 | Plessier A, Belmatoug N, Bureau C, Mathurin P, Larrey D, Chagneau C, et al. | Quality of Life in Patients with Vascular Disease of the Liver: Results of a Questionnaire Survey in 49 Patients with Consolidated Liver Disease | 2010 |
| **599** | 4 | Kramer F, Löning M, Hertel H, Hillemanns P | A Survey of the Application of Sentinel Lymph Node Biopsy Versus Complete Inguinofemoral Lymphadenectomy in Patients with Vulvar Cancer in Germany | 2010 |
| **600** | 4 | Killeen C, Ehrenpreis ED, Du H | Symptoms and Quality of Life (Qol) in a Cohort of Patients with Mesenteric Panniculitis | 2010 |
| **601** | 4 | Terui T | Therapeutic Guidelines for Generalized Pustular Psoriasis Based on Unique Criteria and Classification of Disease Severity 2010 | 2010 |
| **602** | 4 | n/a | 051- | 2010 |
| **603** | 4 | Sanguin S, Daraï E, Brzakowski M, Gondry J | Vulvar Cancer - Survey of Surgical Practice in France in 2009 | 2010 |
| **604** | 4 | Barton D | Cross-Border Genetic Testing | 2010 |
| **605** | 4 | Burtin P, Bouché O, Lombard-Bohas C, Manfredi S. Pezet D, Giroux J, et al. | Gastric Linitis: Results of a French National Survey Confirming the Specificity of This Gastric Neoplasm and the Limited Therapeutic Options | 2010 |
| **606** | 4 | - Campos DJ, Koerich A, Biagini GL, Bonfim C.M.S, - Zanis Neto J, Boguszewski C.L, et al. | Hematopoietic Stem Cell Transplantation in Patients with Fanconi Anemia: What Are the Nutritional Consenquences? | 2010 |
| **607** | 4 | Ahn YM | Hereditary Angioedema in Childhood Presentation of Data from 15 Danish Children | 2010 |
| **608** | 4 | Chang YL, Yang WS, Tu YP | Hydronephrosis Caused by Psoas Muscle Abscess with Candida Albicans Infection - Case Report and Literature Review | 2010 |
| **609** | 4 | - Fahrner B, Prosch H, Minkov M, Wnorowski M, Gadner H, Prayer D, et al. | Hypothalamic-Pituitary Tumors in Langerhans Cell Histiocytosis | 2010 |
| **610** | 4 | Eriksson MK, Malmgren-Olsson EB, Hagberg LA, Eliasson M | Lifestyle Intervention, Quality of Life and Cost-Effectiveness, a Randomized Controlled Trial | 2010 |
| **611** | 4 | Acquaye AA, Vera-Bolanos E, Armstrong TS, Bekele BN, Gilbert MR | Symptom Profiles in Adult Patients with Ependymoma: Report from the Ependymoma Outcomes (Eo) Project | 2010 |
| **612** | 4 | Xu H, Choi JW, Nandram | A Bayesian Adjustment for an Ascertainment Bias in Human Genetics | 2009 |
| **613** | 4 | Wozniak M, Woynarowski M, Chlebcewicz Szuba W, et al. | Burden of Pediatric Autoimmune Hepatitis. Epidemiological Snap Shot from Selected Center in Poland | 2009 |
| **614** | 4 | Tsuchiya-Suzuki A, Yazaki M, Nakamura A, Yamazaki K, Agematsu K, Matsuda M, Ikeda S | Clinical and Genetic Features of Familial Mediterranean Fever in Japan | 2009 |
| **615** | 4 | Craig F, Thomas K, Layfield C, Ingram J, Muller S, Ismail MA, et al. | Management of Pyoderma Gangrenosum by U.K. Dermatologists: A Pilot Study to Inform a Trial | 2009 |
| **616** | 4 | Axelsson A, Jasen E | Necrotizing Fasciitis - a Report on Incidence and Mortality in Denmark in the Years 2005-2007 and a Review of Literature | 2009 |
| **617** | 4 | Park YB, Lee SD, Kim HJ, Lee SM | Pulmonary Arterial Hypertension in Korea. Results from a National Registry; Preliminary Data | 2009 |
| **618** | 4 | Bastani P, Pourmohamadi K, Karimi M | Quality of Life in Hemophilia Complicated by Inhibitors | 2009 |
| **619** | 4 | Li H, Geng L, Guo H, Qiao J | Risk Factors for Vaginal Intraepithelial Neoplasia in China | 2009 |
| **620** | 4 | Woynarowski M, Woźniak M,  Pawłowska M, et al. | Autoimmune Hepatitis in Polish Children: Healthcare Facilities, Epidemiology, and Standards of Care Assessed by a Pediatric Autoimmune Hepatitis Group | 2008 |
| **621** | 4 | Stych B, Dobrovolny D. Familial cold auto-inflammatory syndrome (FCAS): characterization of symptomatology and impact on patients' lives | Familial Cold Auto-Inflammatory Syndrome (Fcas): Characterization of Symptomatology and Impact on Patients' Lives | 2008 |
| **622** | 4 | Mehta A, Ginsberg L; FOS Investigators. Natural history of the cerebrovascular complications of Fabry disease. Acta Paediatr Suppl | Natural History of the Cerebrovascular Complications of Fabry Disease | 2005 |
| **623** | 4 | Williams PH | Perceptions and values regarding DNA contribution to genetic biobanks: survey design, generation and testing | 2009 |
| **624** | 4 | Shimon I, Badiu C, Bossowski A, Doknic M, Dzivite-Krisane I, Hána V, et al. | Adult Growth Hormone Deficiency in Cee Region: Heterogeneity of the Patient Pathway | 2019 |
| **625** | 4 | Kambouchner M, Emile JF, Copin MC, Coulomb-Lherminé A, Sabourin JC, Della Valle V, et al. | Childhood Pulmonary Langerhans Cell Histiocytosis: A Comprehensive Clinical-Histopathological and Brafv600e Mutation Study from the French National Cohort | 2019 |
| **626** | 4 | Chorostowska-Wynimko J, Wencker M, Horváth I | The Importance of Effective Registries in Pulmonary Diseases and How to Optimize Their Output | 2019 |
| **627** | 4 | Buka I, Hervouet-Zeiber C | Lead Toxicity with a New Focus: Addressing Low-Level Lead Exposure in Canadian Children | 2019 |
| **628** | 4 | Oehrlein EM, Love TR, Anyanwu C, Hanna ML, Kraska J, Perfetto EM | Multi-Method Patient-Engagement Approach: A Case Example from a Pcori-Funded Training Project | 2019 |
| **629** | 4 | Torreggiani S, Torcoletti M, Campos-Xavier B, Baldo F, Agostoni C, Superti-Furga A, et al. | Progressive Pseudorheumatoid Dysplasia: A Rare Childhood Disease | 2019 |
| **630** | 4 | Sahay KM, Smith T, Conway KM, Romitti PA, Lamb MM, Andrews J, et al. | A Review of Md Star Net's Research Contributions to Pediatric-Onset Dystrophinopathy in the United States; 2002-2017 | 2019 |
| **631** | 4 | Powell P, Kreuter M, Wijsenbeek-Lourens M | Where Are the Gaps in Education in the Field of Rare Lung Disease? Perspectives from the Ern-Lung Educational Programme Survey | 2019 |
| **632** | 4 | Rice JB, White AG, Scarpati LM, Wan GJ, Nelson WW | The Burden of Non-Infectious Intraocular Inflammatory Eye Diseases: A Systematic Literature Review | 2018 |
| **633** | 4 | Liu KX, Prajapati VH, Liang MG, Mulliken JB, Lee MS | A Cross-Sectional Survey of Long-Term Outcomes for Patients with Diffuse Capillary Malformation with Overgrowth | 2018 |
| **634** | 4 | Cardarelli WJ | Economic Burden Limiting Proper Healthcare Delivery, Management, and Improvement of Patient Outcomes | 2018 |
| **635** | 4 | Swoboda KJ, Nery FC | Harnessing the Power of the Patient Perspective for Rare Disease Therapeutics | 2018 |
| **636** | 4 | Knoble N, Nayroles G, Cheng C, Arnould B | Illustration of Patient-Reported Outcome Challenges and Solutions in Rare Diseases: A Systematic Review in Cushing's Syndrome | 2018 |
| **637** | 4 | Hiremath G, Kodroff E, Strobel MJ, Scott M, Book W, Reidy C, et al. | Individuals Affected by Eosinophilic Gastrointestinal Disorders Have Complex Unmet Needs and Frequently Experience Unique Barriers to Care | 2018 |
| **638** | 4 | Birn CS | Mastocytosis | 2018 |
| **639** | 4 | Slade A, Isa F, Kyte D, Pankhurst T, Kerecuk L, Ferguson J, et al. | Patient Reported Outcome Measures in Rare Diseases: A Narrative Review | 2018 |
| **640** | 4 | Honoré C, Atallah V, Mir O, Orbach D, Ferron G, LePéchoux C, et al. | Abdominal Desmoplastic Small Round Cell Tumor without Extraperitoneal Metastases: Is There a Benefit for Hipec after Macroscopically Complete Cytoreductive Surgery? | 2017 |
| **641** | 4 | Bellutti Enders F, Bader-Meunier B, Baildam E, et al. | Consensus-Based Recommendations for the Management of Juvenile Dermatomyositis | 2017 |
| **642** | 4 | Acaster S | Patient-Reported Outcome and Observer-Reported Outcome Assessment in Rare Disease Trials | 2017 |
| **643** | 4 | Tadisina KK, Lin AY | Squamosal Craniosynostosis: Defining the Phenotype and Indications for Surgical Management | 2017 |
| **644** | 4 | Muenzer J, Jones SA, Tylki-Szymańska A, Harmatz P, Mendelsohn NJ, Guffon N, | Ten Years of the Hunter Outcome Survey (Hos): Insights, Achievements, and Lessons Learned from a Global Patient Registry | 2017 |
| **645** | 4 | Pope JE | Trial Methodology in Scleroderma: Avoiding a Shot in the Dark | 2017 |
| **646** | 4 | anonymous | What's Happening. Rare Disease Caregiving Study | 2017 |
| **647** | 4 | Balasubramaniam GS, Arenas-Hernandez M, Escuredo E, Fairbanks L, Marinaki T, Mapplebeck S, Sheaff M, Almond MK | Adenine Phosphoribosyltransferase Deficiency in the United Kingdom: Two Novel Mutations and a Cross-Sectional Survey | 2016 |
| **648** | 4 | Rüther A, Elstein D, Wong-Rieger D, Guyatt G | Aspects of Patient Reported Outcomes in Rare Diseases: A Discussion Paper | 2016 |
| **649** | 4 | Okubo Y, Yokose T, Motohashi O, Miyagi Y, Yoshioka E, Suzuki M, Washimi K, Kawachi K, Nito M, Nemoto T, Shibuya K, Kameda Y | Duodenal Rare Neuroendocrine Tumor: Clinicopathological Characteristics of Patients with Gangliocytic Paraganglioma | 2016 |
| **650** | 4 | Kitzinger HB, van Schoonhoven J, Schmitt R, Hacker S, Karle B. | Hypothenar Hammer Syndrome: Long-Term Results after Vascular Reconstruction | 2016 |
| **651** | 4 | Davies W | Insights into Rare Diseases from Social Media Surveys | 2016 |
| **652** | 4 | Klein D | An Introduction to the Patient Access Network Foundation and Ajmc Collaborative Supplement | 2016 |
| **653** | 4 | Johnston BC, Miller PA, Agarwal A, et al. | Limited Responsiveness Related to the Minimal Important Difference of Patient-Reported Outcomes in Rare Diseases | 2016 |
| **654** | 4 | Garau R | The Medical Experience of a Patient with a Rare Disease and Her Family | 2016 |
| **655** | 4 | Bronstein MG, Kakkis ED | Patients as Key Partners in Rare Disease Drug Development | 2016 |
| **656** | 4 | Johnson J, Adams-Spink G, Arndt T, Wijeratne D, Heyhoe J, Taylor P | Providing Family-Centred Care for Rare Diseases in Maternity Services: Parent Satisfaction and Preferences When Dysmelia Is Identified | 2016 |
| **657** | 4 | Zion YC, Pappadopulos E, Wajnrajch M, Rosenbaum H | Rethinking Fatigue in Gaucher Disease | 2016 |
| **658** | 4 | Yoneva T, Rashkov R | Rituximab in Treatment of Anca-Associated Vasculitis | 2015 |
| **659** | 4 | Ito T, Lee L, Hijioka M, Kawabe K, Kato M, Nakamura K, et al. | The up-to-Date Review of Epidemiological Pancreatic Neuroendocrine Tumors in Japan | 2015 |
| **660** | 4 | Wang RT, Nelson SF | What Can Duchenne Connect Teach Us About Treating Duchenne Muscular Dystrophy? | 2015 |
| **661** | 4 | Mitsubuchi H, Nakamura K, Matsumoto S, Endo F | Biochemical and Clinical Features of Hereditary Hyperprolinemia | 2014 |
| **662** | 4 | Barnes KT, Smith BJ, Lynch CF, Gupta A | Obesity and Invasive Penile Cancer | 2013 |
| **663** | 4 | Ohga S, Kang D, Kinjo T, Ochiai M, Doi T, Ishimura M, et al. | Paediatric Presentation and Outcome of Congenital Protein C Deficiency in Japan | 2013 |
| **664** | 4 | Lundberg IE, Svensson J | Registries in Idiopathic Inflammatory Myopathies | 2013 |
| **665** | 4 | Zeniya M, Takahashi H | Characteristics of Autoimmune Hepatitis in the Asia-Pacific Region: Historical Review | 2012 |
| **666** | 4 | Riedl M | Hereditary Angioedema Therapies in the United States: Movement toward an International Treatment Consensus | 2012 |
| **667** | 4 | Fasanello RA, Vu TM | Paradoxical Hyponatremia and Polyurodipsia in a Patient with Lithium-Induced Nephrogenic Diabetes Insipidus | 2012 |
| **668** | 4 | Mihai B, Mihai C, Cijevschi-Prelipcean C, Lăcătuşu C | Rare Types of Diabetes Mellitus | 2012 |
| **669** | 4 | Langenbruch AK, Augustin M, Granström S, Kluwe L, Mautner VF | Clinical and Healthcare Status of Patients with Neurofibromatosis Type 1 | 2011 |
| **670** | 4 | Farkas H, Varga L | Ecallantide Is a Novel Treatment for Attacks of Hereditary Angioedema Due to C1 Inhibitor Deficiency | 2011 |
| **671** | 4 | Toscani M, Riedl M | Meeting the Challenges and Burdens Associated with Hereditary Angioedema | 2011 |
| **672** | 4 | Hyde R, Dobrovolny D | Orphan Drug Pricing and Payer Management in the United States: Are We Approaching the Tipping Point? | 2010 |
| **673** | 4 | Soucie JM, McAlister S, McClellan A, Oakley M, Su Y | The Universal Data Collection Surveillance System for Rare Bleeding Disorders | 2010 |
| **674** | 4 | Harmston N, Filsell W, Stumpf MP | What the Papers Say: Text Mining for Genomics and Systems Biology | 2010 |
| **675** | 4 | The L | Listening to Patients with Rare Diseases | 2009 |
| **676** | 4 | Valla DC | Primary Budd-Chiari Syndrome | 2009 |
| **677** | 4 | Ford T | Practitioner Review: How Can Epidemiology Help Us Plan and Deliver Effective Child and Adolescent Mental Health Services? | 2008 |
| **678** | 4 | Heinemann U, Krasnianski A, Meissner B, Gloeckner SF, Kretzschmar HA, Zerr I. | Molecular Subtype-Specific Clinical Diagnosis of Prion Diseases | 2007 |
| **679** | 4 | Shirazi MA, Silver AL, Stankiewicz JA. | Surgical Outcomes Following the Endoscopic Modified Lothrop Procedure | 2007 |
| **680** | 4 | Plaxe SC | Chasing Zebras: The Study and Treatment of Rare Diseases | 2006 |
| **681** | 4 | Ganguli M | Mild Cognitive Impairment and the 7 Uses of Epidemiology | 2006 |
| **682** | 4 | Bolla M, van Poppel H, Collette L, van Cangh P, Vekemans K, Da Pozzo L, et al. | Postoperative Radiotherapy after Radical Prostatectomy: A Randomised Controlled Trial (Eortc Trial 22911) | 2006 |
| **683** | 4 | Senior TP, Knight AW | Rare Diseases Need a Generic Approach [5] | 2006 |
| **684** | 4 | Finger PT | Topical Mitomycin Chemotherapy for Malignant Conjunctival and Corneal Neoplasia | 2006 |
| **685** | 4 | Foot B, Stanford M, Rahi J, Thompson J | The British Ophthalmological Surveillance Unit: An Evaluation of the First 3 Years | 2003 |
| **686** | 4 | Akalan N, Ozgen T | Infection as a Cause of Spinal Cord Compression: A Review of 36 Spinal Epidural Abscess Cases | 2000 |
| **687** | 4 | Papoz L, Balkau B, Lellouch J | Case Counting in Epidemiology: Limitations of Methods Based on Multiple Data Sources | 1996 |
| **688** | 4 | Dickinson CJ | Clinical Research in the Nhs Today | 1994 |
| **689** | 4 | Dans PE, Vernon T, Russell B | Cerebrospinal Fluid Serology - Is Its Routine Use Justifiable? | 1977 |
| **690** | 4 | Borocco C, Ballot-Schmit C, Ackermann O, et al. | The French Paediatric Cohort of Castleman Disease: A Retrospective Report of 23 Patients | 2020 |
| **691** | 4 | Ghaffari-Rafi A, Horak RD, Miles DT, Eum KS, Jahanmir J | Case Report on Fear of Falling Syndrome: A Debilitating but Curable Gait Disorder | 2019 |
| **692** | 4 | Chen CH, Sun CH | Multifocal Head and Neck Amyloidosis as a Diagnostic Clue of Systemic Lupus Erythematosus (Sle): A Case Report | 2019 |
| **693** | 4 | Nezafati S, Yazdani J, Shahi S, Mehryari M, Hajmohammadi E | Outcome of Surgery as Sole Treatment of Eosinophilic Granuloma of Jaws | 2019 |
| **694** | 4 | Prihantono P, Palinggi E, Haryasena H, Hamdani W, Binekada IMC | Surgical Treatment for Parathyroid Adenoma: A Case Report | 2019 |
| **695** | 4 | Riva G, Girolami I, Luchini C, Villanova M, Valotto G, Cima L, et al. | Tracheobronchopathia Osteochondroplastica: A Case Report Illustrating the Importance of Multilevel Workup Clinical, Endoscopic and Histological Assessment in Diagnosis of an Uncommon Disease | 2019 |
| **696** | 4 | Bian S, Chen H, Wang L, Fei Y, Yang Y, Peng L, et al. | Cardiac Involvement in Patients with Primary Biliary Cholangitis: A 14-Year Longitudinal Survey-Based Study | 2018 |
| **697** | 4 | Silveira MAD, Seguro AC, da Silva JB, et al. | Chronic Hyponatremia Due to the Syndrome of Inappropriate Antidiuresis (Siad) in an Adult Woman with Corpus Callosum Agenesis (Cca) | 2018 |
| **698** | 4 | Kim YM, Kang E, Choi JH, Kim GH, Yoo HW, Lee BH | Clinical Characteristics and Treatment Outcomes in Camurati-Engelmann Disease | 2018 |
| **699** | 4 | Diebels I, Blockhuys M, Willemsen P, Pirenne Y | Embolization of a Large Progressive Symptomatic Desmoid Tumor in the Rectus Muscle of a Female Patient with Multiple Sclerosis: A Case Report | 2018 |
| **700** | 4 | Najem CE, Springer J, Prayson R, Culver DA, Fernandez J, Tavee J, et al. | Intra Cranial Granulomatous Disease in Common Variable Immunodeficiency: Case Series and Review of the Literature | 2018 |
| **701** | 4 | Fukayama M, Miyagaki T, Akamata K, Suzuki S, Tanaka M, Sato S | Japanese Familial Anetoderma: A Report of Two Cases and Review of the Published Work | 2018 |
| **702** | 4 | Agaimy A, Fonseca I, Martins C, et al. | Nut Carcinoma of the Salivary Glands: Clinicopathologic and Molecular Analysis of 3 Cases and a Survey of Nut Expression in Salivary Gland Carcinomas | 2018 |
| **703** | 4 | Mufty H, Matricali GA, Thomis S | Venous Malformation as Source of a Tarsal Tunnel Syndrome: Treat the Source or the Cause of the Complaints? A Case Report | 2018 |
| **704** | 4 | Mahoney JM, Millonig K | Behcet's Disease: A Case Report | 2017 |
| **705** | 4 | Taisne N, Desnuelle C, Juntas Morales R, et al. | Bent Spine Syndrome as the Initial Symptom of Late-Onset Pompe Disease | 2017 |
| **706** | 4 | Flamm A, Sachdev S, Dufresne F | Gabapentin-Induced Bullous Pemphigoid | 2017 |
| **707** | 4 | Agarwal P, Kumar V, Kaushal M, Kumari M, Chaudhary A | Indian Visceral Leishmaniasis with Extensive Lymphadenopathy-an Unusual Presentation: A Case Report with Literature Review | 2017 |
| **708** | 4 | Shanahan LK, Raines SG, Coggins RL, Moore T, Carnes M, Griffin L | Osteopathic Manipulative Treatment in the Management of Isaacs Syndrome | 2017 |
| **709** | 4 | Griffiths A, Patel A, Roth MZ | Polyarteritis Nodosum of the Breast in a Patient with History of Bilateral Augmentation Mammoplasty | 2017 |
| **710** | 4 | Chen WT, Huang YC, Peng MC, Wang MC, Lin KP | Acute Disseminated Encephalomyelitis after Influenza Vaccination: A Case Report | 2016 |
| **711** | 4 | Maritati M, Contini C | A Case of Leprosy in Italy: A Multifaceted Disease Which Continues to Challenge Medical Doctors | 2016 |
| **712** | 4 | Yang CS, Lo CP, Wu MC | Ischemic Stroke in a Young Patient with Fahr's Disease: A Case Report | 2016 |
| **713** | 4 | Yadav S, Chalise S, Chaudhary S, Shah GS, Gupta MK, Mishra OP | Osteopetrosis in Two Siblings: Two Case Reports | 2016 |
| **714** | 4 | Papis D, Marangoni G | Surgical Treatment of Megaduodenum in Familial Visceral Myopathy - Report of a Case and Review of the Literature | 2016 |
| **715** | 4 | Erginel B, Gün F, Kocaman H, Çelik A, Salman T | Three Cases of Triple a Syndrome (Allgrove Syndrome) in Pediatric Surgeons' View | 2016 |
| **716** | 4 | Tsai CC, Wu MN | Frequent Ischemic Stroke as First Manifestation of Occult Colon Cancer: A Rare Case | 2015 |
| **717** | 4 | Modi J, Kamal J, Eter A, El-Sayegh S, El-Charabaty E | Immunoglobulin D Multiple Myeloma with Rapidly Progressing Renal Failure | 2015 |
| **718** | 4 | Opotowsky AR, Moko LE, Ginns J, et al. | Pheochromocytoma and Paraganglioma in Cyanotic Congenital Heart Disease | 2015 |
| **719** | 4 | Kumar S, Sharma S | Polyneuropathy, Organomegaly, Endocrinopathy, M-Protein and Skin Changes (Poems Syndrome): A Paraneoplastic Syndrome | 2015 |
| **720** | 4 | Granvall SA | De Garengeot Hernia: A Unique Surgical Finding | 2014 |
| **721** | 4 | Tadano S, Kondo T, Watanabe M, Takayashiki N, Shiozawa T, Ohara G, et al. | Incidental Esophageal Submucosal Tumor Detection by Chest Radiography: A Case Report | 2014 |
| **722** | 4 | Dreznik Y, Gutman M, Weiss B, Nevler A | Mitochondrial Neuro-Gastrointestinal Encephalomyopathy Presenting with Recurrent Bowel Perforations and Intra-Abdominal Abscesses | 2014 |
| **723** | 4 | Schotanus M, van Hout N, Vos D | Pyoderma Gangrenosum of the Hand | 2014 |
| **724** | 4 | Dhinsa BS, Abdul-Jabar HB, Rajkumar S, Kochhar T | A Rare Case of Primary Psoas Abscess Causing Hip Pain in a Patient with Hip Replacement | 2014 |
| **725** | 4 | Anoumou MN, Kouame M, Daix T, Yepie A | Tuberculosis Tenosynovitis of the Flexor Tendons in the Wrist: A Case Report | 2014 |
| **726** | 4 | Gajendra S, Gupta R, Gupta R, Kumar L | Coexistence of Scleroderma with Multiple Myeloma: A Rare Association | 2013 |
| **727** | 4 | Bavisetty S, Grody WW, Yazdani S | Emergence of Pediatric Rare Diseases: Review of Present Policies and Opportunities for Improvement | 2013 |
| **728** | 4 | Asencio-Egea MA, Huertas-Vaquero M, Carranza-González R, et al. | Endogenous Endophthalmitis: Case Report and Brief Review of a Serious Ocular Disease | 2013 |
| **729** | 4 | Veeravagu A, Lad SP, Camara-Quintana JQ, Jiang B, Shuer L | Neurosurgical Interventions for Spondyloepiphyseal Dysplasia Congenita: Clinical Presentation and Assessment of the Literature | 2013 |
| **730** | 4 | Alayli MB, Sanjad SA | Severe Hypoalphalipoproteinaemia in a Child with Acute Post-Streptococcal Glomerulonephritis (Apsgn) | 2013 |
| **731** | 4 | Kunin N, Gancel CH, Foret A, Gayet C, Letoquart JP, Daaboul M | Transiliac Hernia after Bone Graft | 2013 |
| **732** | 4 | Mitu F, Turcanu AM | Chronic Venous Insufficiency Stage V Ceap Secondary to Hereditary Thrombophilia at a Young Man | 2012 |
| **733** | 4 | Costantinides F, Luzzati R, Tognetto D, Bazzocchi G, Biasotto M, Tirelli GC | Rapidly Progressing Subperiosteal Orbital Abscess: An Unexpected Complication of a Group-a Streptococcal Pharyngitis in a Healthy Young Patient | 2012 |
| **734** | 4 | Gupta M, Verma R, Garg RK, Singh M | An Unusual Case of Poems Syndrome | 2012 |
| **735** | 4 | Goujon-Bellec S, Demoury C, Guyot-Goubin A, Hémon D, Clavel J | Detection of Clusters of a Rare Disease over a Large Territory: Performance of Cluster Detection Methods | 2011 |
| **736** | 4 | Al-Haggar M, Sakamoto O, Shaltout A, El-Hawary A, Wahba Y, Abdel-Hadi D | Fanconi Bickel Syndrome: Novel Mutations in Glut 2 Gene Causing a Distinguished Form of Renal Tubular Acidosis in Two Unrelated Egyptian Families | 2011 |
| **737** | 4 | Miyakoshi N, Hongo M, Kasukawa Y, Misawa A, Shimada Y | Bilateral and Symmetric C1-C2 Dumbbell Ganglioneuromas Associated with Neurofibromatosis Type 1 Causing Severe Spinal Cord Compression | 2010 |
| **738** | 4 | Sullivan S | Cannabinoid Hyperemesis | 2010 |
| **739** | 4 | Isreb S, Alem F, Smith D | Left Thyroid Hemiagenesis in a Patient with Primary Hyperparathyroidism | 2010 |
| **740** | 4 | Hausbrandt PA, Leithner A, Beham A, Bodo K, Raith J, Windhager R | A Rare Case of Infantile Myofibromatosis and Review of Literature | 2010 |
| **741** | 4 | Shenoy MM, Gopa K, Girisha BS, Pinto J, Shetty V | Ellis-Van Creveld Syndrome (Chondro-Ectodermal Dysplasia) in Two Siblings | 2008 |
| **742** | 4 | Borrás-Blasco J, Gracia-Perez A, Nuñez-Cornejo C, et al. | Exacerbation of Psoriatic Skin Lesions in a Patient with Psoriatic Arthritis Receiving Adalimumab | 2008 |
| **743** | 4 | Lin CJ, Chang SP, Ke YY, Chiu HY, Tsao LY, Chen M | Phenotype and Genotype of Two Taiwanese Cystic Fibrosis Siblings and a Survey of Delta F508 in East Asians | 2008 |
| **744** | 4 | Yiotakis I, Eleftheriadou A, Giotakis E, Manolopoulos L, Ferekidou E, Kandiloros D | Resection of Giant Ethmoid Osteoma with Orbital and Skull Base Extension Followed by Duraplasty | 2008 |
| **745** | 4 | Famularo G, Minisola G, De Simone C | Rupture of the Spleen after Colonoscopy. A Life-Threatening Complication | 2008 |
| **746** | 4 | Taziarova M, Holeckova K, Lesnakova A, Sladeckova V, Bartkovjak M, Seckova S, et al. | Gram-Negative Bacillary Community Acquired Meningitis Is Not a Rare Entity in Last Two Decades | 2007 |
| **747** | 4 | Mokhtar GA, Yazdi H, Mai KT | Cytopathology of Extramedullary Plasmacytoma of the Bladder: A Case Report | 2006 |
| **748** | 4 | Bosch X, Guilabert A | Kikuchi-Fujimoto Disease | 2006 |
| **749** | 4 | Lagrotteria DD, Tsang B, Elavathil LJ, Tomlinson CW | A Case of Primary Malignant Pericardial Mesothelioma | 2005 |
| **750** | 4 | Geneviève D, Amiel J, Viot G, Le Merrer M, Sanlaville D, Urtizberea A, et al. | Atypical Findings in Kabuki Syndrome: Report of 8 Patients in a Series of 20 and Review of the Literature | 2004 |
| **751** | 4 | Beck M, Ricci R, Widmer U, Dehout F, de Lorenzo AG, Kampmann C, et al. | Fabry Disease: Overall Effects of Agalsidase Alfa Treatment | 2004 |
| **752** | 4 | - Kantarci M, Ogul H, Bayraktutan U | Intracerebral Alveolar Echinococcosis | 2003 |
| **753** | 4 | Wu KL, Changchien CS, Kuo CM, Chuah SK, Chiu YC, Kuo CH. | Caroli's Disease - a Report of Two Siblings | 2002 |
| **754** | 4 | Squarcione S, Prete A, Vellucci L | Botulism Surveillance in Italy: 1992-1996 | 1999 |
| **755** | 4 | Manfredi R, Donzelli C, Talò S, Guzmán SM, Chiodo F | Typhoid Fever and Hiv Infection: A Rare Disease Association in Industrialized Countries | 1998 |
| **756** | 4 | Mircea PA, Valean S, Pop S, et al. | Giant Gastric Trichobezoar | 1997 |
| **757** | 4 | St-Germain G, Murray G, Duperval R | Blastomycosis in Quebec (1981-90): Report of 23 Cases and Review of Published Cases from Quebec | 1993 |
| **758** | 4 | O'Connor TP, Wade TP, Sunwoo YC, Reimers HJ, Palmer DC, Silverberg AB, et al. | Small Cell Undifferentiated Carcinoma of the Pancreas: Report of a Patient with Tumor Marker Studies | 1992 |
| **759** | 4 | Satoh S, Shirane R, Yoshimoto T | Clinical Survey of Ischemic Cerebrovascular Disease in Children in a District of Japan | 1991 |
| **760** | 4 | Harjula ALJ, Harjola P-T, Heikkilä L, Somer T, Nordling S | Aortic and Ureteral Obstruction Associated with Retroperitoneal Fibrosis and Arteritis. A Case Report and Literature Survey | 1985 |
| **761** | 4 | Eroglu M, Taneli NN | Congenital Hyperphosphatasia (Juvenile Paget's Disease). Eleven Years' Follow up of Three Sisters | 1977 |
| **762** | 4 | Quemeneur AS, Trocello JM, Ea HK, Woimant F, Lioté F | Musculoskeletal Conditions Associated with Wilson's Disease | 2011 |
| **763** | 4 | Hirota Y, Suwanai H, Yamauchi T, Kadowaki T | Clinical Features of Type B Insulin Resistance in Japanese Patients: Case Report and Survey-Based Case Series Study | 2020 |
| **764** | 4 | Kazi DS, Bellows BK, Baron SJ, Shen C, Cohen DJ, Spertus JA, et al. | Cost-Effectiveness of Tafamidis Therapy for Transthyretin Amyloid Cardiomyopathy | 2020 |
| **765** | 4 | Guthrie S | Improvement in Quality of Life in Patients with Hereditary Transthyretin Amyloidosis with Polyneuropathy and Cardiomyopathy Treated with Inotersen in the Phase 3 Study Neuro-Ttr | 2020 |
| **766** | 4 | Coelho T, Yarlas A, Waddington-Cruz M, White MK, Sikora Kessler A, Lovley A, et al. | Inotersen Preserves or Improves Quality of Life in Hereditary Transthyretin Amyloidosis | 2020 |
| **767** | 4 | Shafie AA, Supian A, Ahmad Hassali MA, Ngu LH, Thong MK, Ayob H, et al. | Rare Disease in Malaysia: Challenges and Solutions | 2020 |
| **768** | 4 | Cui T, Wang Y, Wang J, Zhang J, Gao Z, Wang Z | The Role of Allogeneic Hematopoietic Stem Cell Transplantation and Epstein-Barr Virus Infection on the Treatment for Child Primary Hemophagocytic Lymphohistiocytosis Patients with X-Linked Lymphoproliferative Disease: A Rare Case Report and Family Survey Study | 2020 |
| **769** | 4 | Louie A, Meyerle C, Francomano C, Srikumaran D, Merali F, Doyle JJ, et al. | Survey of Ehlers-Danlos Patients' Ophthalmic Surgery Experiences | 2020 |
| **770** | 4 | Thombs B.D, Kwakkenbos L, Carrier M.-E, Bourgeault A, Tao L, Harb S, et al. | A Partially Nested RCT to Evaluate the Effectiveness of the Scleroderma Patient-centered Intervention Network COVID-19 Home-isolation Activities Together (SPIN-CHAT) Program to Reduce Anxiety Among At-Risk Scleroderma Patients | 2020 |
| **771** | 4 | Dillard D | Blood Pressure-Improving Control among Alaska Native People" (Bp-Ican) | 2019 |
| **772** | 4 | Hureaux M, Ashton E, Dahan K, Houillier P, Blanchard A, Cormier C, et al. | High-Throughput Sequencing Contributes to the Diagnosis of Tubulopathies and Familial Hypercalcemia Hypocalciuria in Adults | 2019 |
| **773** | 4 | Cooper J | Hyperbaric Oxygen for Carbon Monoxide Induced Chronic Encephalopathy | 2019 |
| **774** | 4 | Dal-Ré R, Avendaño-Solà C, de Boer A, James SK, Rosendaal FR, Stephens R, et al. | A Limited Number of Medicines Pragmatic Trials Had Potential for Waived Informed Consent Following the 2016 Cioms Ethical Guidelines | 2019 |
| **775** | 4 | Salman J, Ius F, Sommer W, Siemeni T, Fleissner F, Alhadidi H, et al. | Long-Term Results of Bilateral Lung Transplantation in Patients with End-Stage Pulmonary Lymphangioleiomyomatosis | 2019 |
| **776** | 4 | Galvão CRC, Cavalcante PMA, Olinda R, Graciani Z, Zatz M, Kok F, et al. | Motor Impairment in a Rare Form of Spastic Paraplegia (Spoan Syndrome): A 10-Year Follow-Up | 2019 |
| **777** | 4 | Greenberg BM, Krishnan C, Harder L | New Onset Transverse Myelitis Diagnostic Accuracy and Patient Experiences | 2019 |
| **778** | 4 | Shiraishi H, Yamada K, Oki E, Ishige M, Fukao T, Hamada Y, et al. | Open-Label Clinical Trial of Bezafibrate Treatment in Patients with Fatty Acid Oxidation Disorders in Japan; 2nd Report Qol Survey | 2019 |
| **779** | 4 | Hasker E, Malaviya P, Scholar VK, de Koning P, Singh OP, Kansal S, et al. | Post Kala Azar Dermal Leishmaniasis and Leprosy Prevalence and Distribution in the Muzaffarpur Health and Demographic Surveillance Site | 2019 |
| **780** | 4 | Walburn J, Canfield M, Norton S, Sainsbury K, Araújo-Soares V, Foster L, et al. | Psychological Correlates of Adherence to Photoprotection in a Rare Disease: International Survey of People with Xeroderma Pigmentosum | 2019 |
| **781** | 4 | Thombs BD | The Spin - Scleroderma Support Group Leader Education Program Trial (Spin-Ssled) | 2019 |
| **782** | 4 | van der Meijden JC, Kruijshaar ME, Rizopoulos D, van Doorn PA, van der Beek NAME, van der Ploeg AT | Enzyme Replacement Therapy Reduces the Risk for Wheelchair Dependency in Adult Pompe Patients | 2018 |
| **783** | 4 | Hull University Teaching Hospitals NHS Trust | Feasibility Study of Early Review and Early Cardiac Rehabilitation after Coronary Artery Bypass | 2018 |
| **784** | 4 | Wainright C | Finding the Optimal Regimen for Mycobacterium Abscessus Treatment (Format) | 2018 |
| **785** | 4 | Boffin N, Swinnen E, Wens J, Urbina M, Van der Heyden J, Van Casteren V | General Practice Care for Patients with Rare Diseases in Belgium. A Cross-Sectional Survey | 2018 |
| **786** | 4 | Shaikh S, Waxler JL, Lee H, Grinke K, Garry J, Pober BR, et al. | Glucose and Lipid Metabolism, Bone Density, and Body Composition in Individuals with Williams Syndrome | 2018 |
| **787** | 4 | Sugie K, Komaki H, Eura N, Shiota T, Onoue K, Tsukaguchi H, et al. | A Nationwide Survey on Danon Disease in Japan | 2018 |
| **788** | 4 | Tingsgaard JK, Henriksen A, Mikkelsen LH, Behrendt N, Melchior LC, Svendsen LB, et al. | Primary and Secondary Mucosal Melanoma of the Small Intestine – a Clinical, Pathological, and Genetic Nationwide Survey of Danish Patients between 1980 and 2014 | 2018 |
| **789** | 4 | Manjang B, Hemming K, Bradley C, Ensink J, Martin JT, Sowe J, et al. | Promoting Hygienic Weaning Food Handling Practices through a Community-Based Programme: Intervention Implementation and Baseline Characteristics for a Cluster Randomised Controlled Trial in Rural Gambia | 2018 |
| **790** | 4 | Thombs BD, Dyas L, Pépin M, Aguila K, Carrier ME, Tao L, et al. | The Scleroderma Patient-Centered Intervention Network (Spin) Support Group Leader Education Program Feasibility Trial | 2018 |
| **791** | 4 | Cao XX, Tian Z, Lin L, Sun J, Su W, Zhou DB, et al. | Successful Treatment of Type 1 Cryoglobulinemic Vasculitis with Cardiac Involvement | 2018 |
| **792** | 4 | Rizio AA, White MK, McCausland KL, Quock TP, Guthrie SD, Yokota M, et al. | Treatment Tolerability in Patients with Immunoglobulin Light-Chain Amyloidosis | 2018 |
| **793** | 4 | Wagenhäuser MU, Meyer-Janiszewski YK, Dueppers P, et al. | Chronic Mesenteric Ischemia: Patient Outcomes Using Open Surgical Revascularization | 2017 |
| **794** | 4 | Choi JH, Lee BH, Heo SH, Kim GH, Kim YM, Kim DS, et al. | Clinical Characteristics and Mutation Spectrum of Gla in Korean Patients with Fabry Disease by a Nationwide Survey: Underdiagnosis of Late-Onset Phenotype | 2017 |
| **795** | 4 | Krause K, Tsianakas A, Wagner N, Fischer J, Weller K, Metz M, et al. | Efficacy and Safety of Canakinumab in Schnitzler Syndrome: A multicenter Randomized Placebo-Controlled Study | 2017 |
| **796** | 4 | D'Ambrosi R, Ragone V, Caldarini C, Serra N, Usuelli FG, Facchini RM | The Impact of Hereditary Multiple Exostoses on Quality of Life, Satisfaction, Global Health Status, and Pain | 2017 |
| **797** | 4 | Isrctn | Patients for Patients – Qualified Peer-Counselling and Self-Management for Patients with Rare Chronic Diseases | 2017 |
| **798** | 4 | Esposito M, Gimigliano F, Barillari MR, Precenzano F, Ruberto M, Sepe J, et al. | Pediatric Selective Mutism Therapy: A Randomized Controlled Trial | 2017 |
| **799** | 4 | Tabibian JH, Gossard A, El-Youssef M, et al. | Prospective Clinical Trial of Rifaximin Therapy for Patients with Primary Sclerosing Cholangitis | 2017 |
| **800** | 4 | Hoenig M, Lagresle-Peyrou C, Pannicke U, Notarangelo LD, Porta F, Gennery AR, et al. | Reticular Dysgenesis: International Survey on Clinical Presentation, Transplantation, and Outcome | 2017 |
| **801** | 4 | Roghi A, Poggiali E, Cassinerio E, Pedrotti P, Giuditta M, Milazzo A, et al. | The Role of Cardiac Magnetic Resonance in Assessing the Cardiac Involvement in Gaucher Type 1 Patients: Morphological and Functional Evaluations | 2017 |
| **802** | 4 | Chiu CY, Su SC, Fan WL, Lai SH, Tsai MH, Chen SH, et al. | Whole-Genome Sequencing of a Family with Hereditary Pulmonary Alveolar Proteinosis Identifies a Rare Structural Variant Involving Csf2ra/Crlf2/Il3ra Gene Disruption | 2017 |
| **803** | 4 | Montaño AM, Lock-Hock N, Steiner RD, Graham BH, Szlago M, Greenstein R, et al. | Clinical Course of Sly Syndrome (Mucopolysaccharidosis Type Vii) | 2016 |
| **804** | 4 | Najafi R, Hashemipour M, Mostofizadeh N, Ghazavi M, Nasiri J, Shahsanai A, et al. | Demographic and Clinical Findings in Pediatric Patients Affected by Organic Acidemia | 2016 |
| **805** | 4 | Deroux A, Boccon-Gibod I, Fain O, Pralong P, Ollivier Y, Pagnier A, et al. | Hereditary Angioedema with Normal C1 Inhibitor and Factor Xii Mutation: A Series of 57 Patients from the French National Center of Reference for Angioedema | 2016 |
| **806** | 4 | D'Angelo R, Rinaldi R, Carelli V, Boschetti E, Caporali L, Capristo M, et al. | Ita-Mngie: An Italian Regional and National Survey for Mitochondrial Neuro-Gastro-Intestinal Encephalomyopathy | 2016 |
| **807** | 4 | McClure P, Booy D, Katarincic J, Eberson C | Orthopedic Manifestations of Mobius Syndrome: Case Series and Survey Study | 2016 |
| **808** | 4 | Lane A, Metcalfe C, Young GJ, Peters TJ, Blazeby J, Avery KN, et al. | Patient-Reported Outcomes in the Protect Randomized Trial of Clinically Localized Prostate Cancer Treatments: Study Design, and Baseline Urinary, Bowel and Sexual Function and Quality of Life | 2016 |
| **809** | 4 | Umazume K, Suzuki J, Usui Y, Wakabayashi Y, Goto H | Possible Relation between Lack of Posterior Vitreous Detachment and Severe Endogenous Endophthalmitis | 2016 |
| **810** | 4 | Plomp RG, van Lieshout MJS, Joosten KFM, et al. | Treacher Collins Syndrome: A Systematic Review of Evidence-Based Treatment and Recommendations | 2016 |
| **811** | 4 | Lund C, Bjørnvold M, Tuft M, Kostov H, Røsby O, Selmer KK | Aicardi Syndrome: An Epidemiologic and Clinical Study in Norway | 2015 |
| **812** | 4 | van Schaarenburg RA, Schejbel L, Truedsson L, Topaloglu R, Al-Mayouf SM, Riordan A, et al. | Marked Variability in Clinical Presentation and Outcome of Patients with C1q Immunodeficiency | 2015 |
| **813** | 4 | Shumack S | A Randomised, Double Blind, Placebo Controlled Comparative Trial of Ceramide Cream and Ceramide Cleanser in the Management of Moderate Eczema in Adults | 2015 |
| **814** | 4 | DRKS | Rehabilitation and Individuals with Temporary Disability Pension | 2015 |
| **815** | 4 | Fujimoto M, Okada S, Kawashima Y, Nishimura R, Miyahara N, Kawaba Y, et al. | Clinical Overview of Nephrogenic Diabetes Insipidus Based on a Nationwide Survey in Japan | 2014 |
| **816** | 4 | .  Dall’Alba L, Gray M, Williams G, Lowe S | Early Intervention in Children (0-6 Years) with a Rare Developmental Disability: The Occupational Therapy Role | 2014 |
| **817** | 4 | Retsch-Bogart GZ, Van Dalfsen JM, Marshall BC, et al. | Highly Effective Cystic Fibrosis Clinical Research Teams: Critical Success Factors | 2014 |
| **818** | 4 | Abe J, Nakamura K, Nishikomori R, et al. | A Nationwide Survey of Aicardi–Goutières Syndrome Patients Identifies a Strong Association between Dominant Trex1 Mutations and Chilblain Lesions: Japanese Cohort Study | 2014 |
| **819** | 4 | Chan AK, Takano A, Hsu AL, Low SY | Pulmonary Alveolar Proteinosis | 2014 |
| **820** | 4 | Nordenfelt P, Dawson S, Wahlgren CF, Lindfors A, Mallbris L, Björkander J | Quantifying the Burden of Disease and Perceived Health State in Patients with Hereditary Angioedema in Sweden | 2014 |
| **821** | 4 | Berkouk-Redjimi Y, Belhani MF | Rare Congenital Coagulation Factor Deficiencies: Clinical Manifestations | 2014 |
| **822** | 4 | Walat A, Skoczylas MM, Welnicka A, et al. | Szczecin Citizens' Knowledge About Rare Diseases | 2014 |
| **823** | 4 | Stunnenberg BC, Woertman W, Raaphorst J, et al. | Combined N-of-1 Trials Mexiletine Vs Placebo in Patients with Non-Dystrophic Myotonia (Ndm) | 2013 |
| **824** | 4 | Sakushima K, Hida K, Yabe I, Tsuboi S, Uehara R, Sasaki H | Different Surgical Treatment Techniques Used by Neurosurgeons and Orthopedists for Syringomyelia Caused by Chiari I Malformation in Japan | 2013 |
| **825** | 4 | Hasib Ahmed AI | Feasibility of a Trial of Laparoscopic Hysterectomy Versus Laparoscopic Sub-Total Hysterectomy | 2013 |
| **826** | 4 | King MJ, Zanella A | Hereditary Red Cell Membrane Disorders and Laboratory Diagnostic Testing | 2013 |
| **827** | 4 | Aboul-Enein F, Seifert-Held T, Mader S, Kuenz B, Lutterotti A, Rauschka H, et al. | Neuromyelitis Optica in Austria in 2011: To Bridge the Gap between Neuroepidemiological Research and Practice in a Study Population of 8.4 Million People | 2013 |
| **828** | 4 | Hosman AE, Devlin HL, Silva BM, Shovlin CL. | Specific Cancer Rates May Differ in Patients with Hereditary Haemorrhagic Telangiectasia Compared to Controls | 2013 |
| **829** | 4 | Weintraub D, Duda JE, Carlson K, Luo P, Sagher O, Stern M, et al. | Suicide Ideation and Behaviours after Stn and Gpi Dbs Surgery for Parkinson's Disease: Results from a Randomised, Controlled Trial | 2013 |
| **830** | 4 | Hamidieh AA, Ansari S, Darbandi B, Soroush A, Arjmandi Rafsanjani K, Alimoghaddam K, et al. | The Treatment of Children Suffering from Chronic Myelogenous Leukemia: A Comparison of the Result of Treatment with Imatinib Mesylate and Allogeneic Hematopoietic Stem Cell Transplantation | 2013 |
| **831** | 4 | Giamarellos-Bourboulis EJ | Anakinra in Hidradenitis Suppurativa | 2012 |
| **832** | 4 | Bastidas N, Mackay DDJ, Taylor JA, Bartlett SP | Analysis of the Long-Term Outcomes of Nonsyndromic Bicoronal Synostosis | 2012 |
| **833** | 4 | Brandenburg VM, Kramann R, Specht P, Ketteler M | Calciphylaxis in Ckd and Beyond | 2012 |
| **834** | 4 | Lu DF, Moon M, Lanning LD, McCarthy AM, Smith RJ | Clinical Features and Outcomes of 98 Children and Adults with Dense Deposit Disease | 2012 |
| **835** | 4 | Harms L, Sieb JP, Williams AE, Graham R, Shlaen R, Claus V, et al. | Long-Term Disease History, Clinical Symptoms, Health Status, and Healthcare Utilization in Patients Suffering from Lambert Eaton Myasthenic Syndrome: Results of a Patient Interview Survey in Germany | 2012 |
| **836** | 4 | Li H, Guo YL, Zhang JX, Qiao J, Geng L | Risk Factors for the Development of Vaginal Intraepithelial Neoplasia | 2012 |
| **837** | 4 | Nct | Wick Vs. No Wick: Does Method of Closure Affect Rate of Wound Infection? | 2012 |
| **838** | 4 | Piarroux M, Piarroux R, Giorgi R, Knapp J, Bardonnet K, Sudre B, et al. | Clinical Features and Evolution of Alveolar Echinococcosis in France from 1982 to 2007: Results of a Survey in 387 Patients | 2011 |
| **839** | 4 | Isrctn | The Fixed Bearing Lateral Stabilised Djo 3d Knee™ Versus the Finsbury Medial Rotation Knee™ System Versus the Stryker Triathlon® Knee in Primary Total Knee Replacement for Osteoarthritis and Rheumatoid Arthritis | 2011 |
| **840** | 4 | Nct | Phenoxybenzamine Versus Doxazosin in Pcc Patients | 2011 |
| **841** | 4 | Kamdar BB, Nandkumar P, Krishnan V, Gamaldo CE, Collop NA | Self-Reported Sleep and Breathing Disturbances in Joubert Syndrome | 2011 |
| **842** | 4 | Isomoto H, Ohnita K, Yamaguchi N, Fukuda E, Ikeda K, Nishiyama H, et al. | Clinical Outcomes of Endoscopic Submucosal Dissection in Elderly Patients with Early Gastric Cancer | 2010 |
| **843** | 4 | Edener U, Wöllner J, Hehr U, Kohl Z, Schilling S, Kreuz F, et al. | Early Onset and Slow Progression of Sca28, a Rare Dominant Ataxia in a Large Four-Generation Family with a Novel Afg3l2 Mutation | 2010 |
| **844** | 4 | Fearon WF, Bornschein B, Tonino PA, Gothe RM, Bruyne BD, Pijls NH, et al. | Economic Evaluation of Fractional Flow Reserve-Guided Percutaneous Coronary Intervention in Patients with Multivessel Disease | 2010 |
| **845** | 4 | Yuan J, Li XH. | Evaluation of Pathological Diagnosis Using Ultrasonography-Guided Lymph Node Core-Needle Biopsy | 2010 |
| **846** | 4 | Isrctn | Aortic Valve Replacement in High Risk Patients: Conventional Surgery Compared with Catheter-Based Techniques | 2009 |
| **847** | 4 | Qin RF, Shi LF, Liu YP, Lei DL, Hu KJ, Feng XH, et al. | Diagnosis and Surgical Treatment of Carotid Body Tumors: 25 Years' Experience in China | 2009 |
| **848** | 4 | Mukherjee A, McGarrity T, Staveley-O'Carroll K, Ruggiero F, Baker MJ | Hereditary Diffuse Gastric Cancer: Lifesaving Potential of Prophylactic Gastrectomy | 2009 |
| **849** | 4 | Michel M, Chanet V, Dechartres A, Morin AS, Piette JC, Cirasino L, et al. | The Spectrum of Evans Syndrome in Adults: New Insight into the Disease Based on the Analysis of 68 Cases | 2009 |
| **850** | 4 | Nozu T, Komiyama H | Clinical Characteristics of Asymptomatic Esophagitis | 2008 |
| **851** | 4 | International Centre for Diarrhoeal Disease Research, Bangladesh | Iron Deficiency Anemia and Psychosocial Stimulation | 2008 |
| **852** | 4 | Pirrotta MT, Bernardeschi P, Fiorentini G | Targeted Therapy for Advanced Renal Cell Carcinoma | 2008 |
| **853** | 4 | Pollock-BarZiv S, Cohen MM, Downey GP, Johnson SR, Sullivan E, McCormack FX | Air Travel in Women with Lymphangioleiomyomatosis | 2007 |
| **854** | 4 | Kala Z, Válek V, Kysela P | Amyloidosis of the Small Intestine | 2007 |
| **855** | 4 | Wolff AS, Erichsen MM, Meager A, Magitta NF, Myhre AG, et al. | Autoimmune Polyendocrine Syndrome Type 1 in Norway: Phenotypic Variation, Autoantibodies, and Novel Mutations in the Autoimmune Regulator Gene | 2007 |
| **856** | 4 | Mills EJ, Kelly S, Wu P, Guyatt GH | Epidemiology and Reporting of Randomized Trials Employing Re-Randomization of Patient Groups: A Systematic Survey | 2007 |
| **857** | 4 | Mennel S, Barbazetto I, Meyer CH, Peter S, Stur M | Ocular Photodynamic Therapy - Standard Applications and New Indications (Part 2): Review of the Literature and Personal Experience | 2007 |
| **858** | 4 | Mann GN, Link JM, Pham P, Pickett CA, Byrd DR, Kinahan PE, et al. | [11c]Metahydroxyephedrine and [18f]Fluorodeoxyglucose Positron Emission Tomography Improve Clinical Decision Making in Suspected Pheochromocytoma | 2006 |
| **859** | 4 | Schoening AM | Amniotic Fluid Embolism: Historical Perspectives & New Possibilities | 2006 |
| **860** | 4 | Ashraf-Ganjoei T, Behtash N, Shariat M, Mosavi A | Low Grade Endometrial Stromal Sarcoma of Uterine Corpus, a Clinico-Pathological and Survey Study in 14 Cases | 2006 |
| **861** | 4 | Hashimoto J, Ohno I, Nakatsuka K, Yoshimura N, Takata S, Zamma M, et al. | Prevalence and Clinical Features of Paget's Disease of Bone in Japan | 2006 |
| **862** | 4 | Nct | Assessing Impact of Loco-Regional Treatment on Survival in Metastatic Breast Cancer at Presentation | 2005 |
| **863** | 4 | Boesenberg C, Schulz-Schaeffer WJ, Meissner B, et al. | Clinical Course in Young Patients with Sporadic Creutzfeldt-Jakob Disease | 2005 |
| **864** | 4 | Hyduk A, Croft JB, Ayala C, Zheng K, Zheng ZJ, Mensah GA | Pulmonary Hypertension Surveillance -- United States, 1980-2002 | 2005 |
| **865** | 4 | Chen, HW, Su DH, Chen, HW, Chang TC | Thyroid Carcinomas with Brain or Skull Metastases | 2005 |
| **866** | 4 | Mehta A, Ricci R, Widmer U, Dehout F, Garcia de Lorenzo A, Kampmann C, et al. | Fabry Disease Defined: Baseline Clinical Manifestations of 366 Patients in the Fabry Outcome Survey | 2004 |
| **867** | 4 | Jenkins PA, Campbell IA; Research Committee of The British Thoracic Society | Pulmonary Disease Caused by Mycobacterium Xenopi in Hiv-Negative Patients: Five Year Follow-up of Patients Receiving Standardised Treatment | 2003 |
| **868** | 4 | Ronghe MD, Barton J, Jardine PE, Crowne EC, Webster MH, Armitage M, et al. | The Importance of Testing for Adrenoleucodystrophy in Males with Idiopathic Addison's Disease | 2002 |
| **869** | 4 | Aihara Y, Takahashi Y, Kotoyori T, Mitsuda T, Ito R, Aihara M, et al. | Frequency of Food-Dependent, Exercise-Induced Anaphylaxis in Japanese Junior-High-School Students | 2001 |
| **870** | 4 | Kim DS | Sarcoidosis in Korea: Report of the Second Nationwide Survey | 2001 |
| **871** | 4 | Leung T, Slinger R, Mindorff C,  Ricketts M, Cranston B, Paton S | Creutzfeldt-Jakob Disease: Infection Control Policies and Neurosurgical Instrument Processing Capabilities in Canadian Neurosurgical Hospitals | 2000 |
| **872** | 4 | Applegarth DA, Toone JR, Lowry RB | Incidence of Inborn Errors of Metabolism in British Columbia, 1969-1996 | 2000 |
| **873** | 4 | Topaloglu R, Bakkaloglu A, Slingsby JH, Aydintug O, Besbas N, Saatci U, et al. | Survey of Turkish Systemic Lupus Erythematosus Patients for a Particular Mutation of C1q Deficiency | 2000 |
| **874** | 4 | Bresson-Hadni S, Vuitton DA, Bartholomot B, Heyd B, Godart D, Meyer JP, et al. | A Twenty-Year History of Alveolar Echinococcosis: Analysis of a Series of 117 Patients from Eastern France | 2000 |
| **875** | 4 | Ben-Dov I, Kishinevski Y, Roznman J, et al. | Pulmonary Alveolar Proteinosis in Israel: Ethnic Clustering | 1999 |
| **876** | 4 | Nielson KA, Nolan JH, Berchtold NC, Sandman CA, Mulnard RA, Cotman CW | Apolipoprotein-E Genotyping of Diabetic Dementia Patients: Is Diabetes Rare in Alzheimer's Disease? | 1996 |
| **877** | 4 | van der Kooi AJ, Barth PG, Busch HF, de Haan R, Ginjaar HB, van Essen AJ, et al. | The Clinical Spectrum of Limb Girdle Muscular Dystrophy. A Survey in the Netherlands | 1996 |
| **878** | 4 | Geissmann F, Thomas C, Emile JF, Micheau M, Canioni D, Cerf-Bensussan N, et al. | Digestive Tract Involvement in Langerhans Cell Histiocytosis. The French Langerhans Cell Histiocytosis Study Group | 1996 |
| **879** | 4 | Karma A, Mikkilä H | Ocular Manifestations and Treatment of Lyme Disease | 1996 |
| **880** | 4 | Lerede T, Bassan R, Rossi A, Di Bona E, Rossi G, Pogliani EM, et al. | Therapeutic Impact of Adult-Type Acute Lymphoblastic Leukemia Regimens in B-Cell/L3 Acute Leukemia and Advanced-Stage Burkitt's Lymphoma | 1996 |
| **881** | 4 | Peñas PF, Rios-Buceta L, Sánchez-Pérez J, Dorado-Bris JM, Aragüés M | Keratosis Punctata of the Palmar Creases: Case Report and Prevalence Study in Caucasians | 1994 |
| **882** | 4 | ELLIOTT EJ, CHANT K. G. | Rare Disease Surveillance | 1994 |
| **883** | 4 | Riis P | A Critical Survey of Controlled Studies in the Treatment of Ulcerative Colitis and Crohn's Disease | 1980 |
| **884** | 4 | Kubo S, Funabashi S, Uehara S, Toba T, Muramatsu Y, Sanada K | Clinical Aspects of 'Asthmatic Bronchitis' and Chronic Bronchitis in Infants and Children | 1978 |
| **885** | 4 | Tsujimoto M | Epidemiological Research on the Prevalence of Ankylosing Spondylitis | 1978 |
| **886** | 4 | Evans AE, D'Angio GJ, Koop CE | Childhood Cancer: Basic Considerations in Diagnosis and Treatment | 1976 |
| **887** | 4 | Shai S, Perez-Becker R, von König CH, et al. | Rotavirus Disease in Germany-a Prospective Survey of Very Severe Cases | 2013 |
| **888** | 4 | Kanecki K, Nitsch-Osuch A, Gorynski P, Tarka P, Tyszko P. | Hospital morbidity database for epidemiological studies on Churg-Strauss syndrome | 2017 |
| **889** | 4 | Baynam G, Bowman F, Lister K, Walker CE, Pachter N, Goldblatt J, et al. | Improved diagnosis and care for rare diseases through implementation of precision public health framework | 2017 |
| **890** | 5 | Hansmann S, Lainka E, Horneff G, Holzinger D, Rieber N, Jansson AF, et al. | Consensus Protocols for the Diagnosis and Management of the Hereditary Autoinflammatory Syndromes Caps, Traps and Mkd/Hids: A German Pro-Kind Initiative | 2020 |
| **891** | 5 | Clairman H, Dover S, Whitney K, Marcuz JA, Bell-Peter A, Feldman BM | Correlation of a Modified Disease Activity Score with the Validated Original Disease Activity Score in Patients with Juvenile Dermatomyositis | 2020 |
| **892** | 5 | de Vries F, Bruin M, Cersosimo A, van Beuzekom CN, Ahmed SF, et al. | An Overview of Clinical Activities in Endo-Ern: The Need for Alignment of Future Network Criteria | 2020 |
| **893** | 5 | Sage L, Russo ML, Byers PH, Demasi J, Morris SA, Puryear LN, et al. | Setting a Research Agenda for Vascular Ehlers-Danlos Syndrome Using a Patient and Stakeholder Engagement Model | 2020 |
| **894** | 5 | Salek MS, Ionova T, Johns JR, Oliva EN | Appraisal of Patient-Reported Outcome Measures in Analogous Diseases and Recommendations for Use in Phase Ii and Iii Clinical Trials of Pyruvate Kinase Deficiency | 2019 |
| **895** | 5 | Kaiser K, Yount SE, Martens CE, Webster KA, Shaunfield S, Sparling A, et al. | Assessing Patient Preferences for Rare Disease Treatment: Qualitative Development of the Paroxysmal Nocturnal Hemoglobinuria Patient Preference Questionnaire | 2019 |
| **896** | 5 | Hollin IL, Dimmock AE, Bridges JF, Danoff SK, Bascom R | Collecting Patient Preference Information Using a Clinical Data Research Network: Demonstrating Feasibility with Idiopathic Pulmonary Fibrosis | 2019 |
| **897** | 5 | Foeldvari I, Culpo R, Sperotto F, Anton J, Avcin T, Baildam E, et al. | Consensus-Based Recommendations for the Management of Juvenile Localised Scleroderma | 2019 |
| **898** | 5 | Horgan D, Bernini C, Thomas PPM, Morre SA. | Cooperating on Data: The Missing Element in Bringing Real Innovation to Europe's Healthcare Systems | 2019 |
| **899** | 5 | Kocher A, Simon M, Dwyer AA, Villiger PM, Künzler-Heule P, De Geest S, et al. | Developing a Rare Disease Chronic Care Model: Management of Systemic Sclerosis (Manoss) Study Protocol | 2019 |
| **900** | 5 | Cruz R, Belter L, Wasnock M, Nazarelli A, Jarecki J | Evaluating Benefit-Risk Decision-Making in Spinal Muscular Atrophy: A First-Ever Study to Assess Risk Tolerance in the Sma Patient Community | 2019 |
| **901** | 5 | Efthymiadou O, Mossman J, Kanavos P | Health Related Quality of Life Aspects Not Captured by Eq-5d-5l: Results from an International Survey of Patients | 2019 |
| **902** | 5 | van Karnebeek CDM, Beumer D, Pawliuk C, Goez H, Mostafavi S, et al. | A Novel Classification System for Research Reporting in Rare and Progressive Genetic Conditions | 2019 |
| **903** | 5 | Park Y, Fullerton HJ, Elm JJ | A Pragmatic, Adaptive Clinical Trial Design for a Rare Disease: The Focal Cerebral Arteriopathy Steroid (Focas) Trial | 2019 |
| **904** | 5 | Montgomery BK, Cahan EM, Frick S | Spinal Screening Mri Trends in Patients with Multiple Hereditary Exostoses: National Survey | 2019 |
| **905** | 5 | Marques R, Belousova E, Benedik MP, Carter T, Cottin V, Curatolo P, et al. | The Tosca Registry for Tuberous Sclerosis—Lessons Learnt for Future Registry Development in Rare and Complex Diseases | 2019 |
| **906** | 5 | Noël E, Dussol B, Lacombe D, Bedreddine N, Fouilhoux A, Ronco P, et al. | Treatment Needs and Expectations for Fabry Disease in France: Development of a New Patient Needs Questionnaire | 2019 |
| **907** | 5 | Wei Y, MSc, McCo rmick  A, et al. | The Canadian Neuromuscular Disease Registry: Connecting Patients to National and International Research Opportunities | 2018 |
| **908** | 5 | Garcelon N, Neuraz A, Salomon R, Faour H, Benoit V, Delapalme A, et al. | A Clinician Friendly Data Warehouse Oriented toward Narrative Reports: Dr. Warehouse | 2018 |
| **909** | 5 | Grinspan ZM, Tian N, Yozawitz EG, McGoldrick PE, Wolf SM, McDonough TL, et al. | Common Terms for Rare Epilepsies: Synonyms, Associated Terms, and Links to Structured Vocabularies | 2018 |
| **910** | 5 | Stacpoole PW, Shuster J, Thompson JLPS, Prather RA, Lawson LA, Zou B, et al. | Development of a Novel Observer Reported Outcome Tool as the Primary Efficacy Outcome Measure for a Rare Disease Randomized Controlled Trial | 2018 |
| **911** | 5 | Rapkin BD, Garcia I, Michael W, Zhang J, Schwartz CE | Development of a Practical Outcome Measure to Account for Individual Differences in Quality-of-Life Appraisal: The Brief Appraisal Inventory | 2018 |
| **912** | 5 | Namulanda G, Qualters J, Vaidyanathan A, Roberts E, Richardson M, Fraser A, et al. | Electronic Health Record Case Studies to Advance Environmental Public Health Tracking | 2018 |
| **913** | 5 | Botto LD, Mastroiacovo P | From Cause to Care: Triple Surveillance for Better Outcomes in Birth Defects and Rare Diseases | 2018 |
| **914** | 5 | Marcucci G, Cianferotti L, Parri S, et al. | Hypoparanet: A Database of Chronic Hypoparathyroidism Based on Expert Medical-Surgical Centers in Italy | 2018 |
| **915** | 5 | Pinera EK, Arteagoitia GM, González LR, Lamiquiz LE | Iexpac, a Tool to Assess Patient Experience | 2018 |
| **916** | 5 | Williams JL, Rahm AK, Zallen DT, et al. | Impact of a Patient-Facing Enhanced Genomic Results Report to Improve Understanding, Engagement, and Communication | 2018 |
| **917** | 5 | Ahern S, Sims G, Earnest A, C Bell S | Optimism, Opportunities, Outcomes: The Australian Cystic Fibrosis Data Registry | 2018 |
| **918** | 5 | Slota C, Bevans M, Yang L, Shrader J, Joe G, Carrillo N | Patient Reported Outcomes in Gne Myopathy: Incorporating a Valid Assessment of Physical Function in a Rare Disease | 2018 |
| **919** | 5 | Karanevich AG, Statland JM, Gajewski BJ, He J | Using an Onset-Anchored Bayesian Hierarchical Model to Improve Predictions for Amyotrophic Lateral Sclerosis Disease Progression | 2018 |
| **920** | 5 | COST Action BM1105, Badiu C, Bonomi M, Borshchevsky I, Cools M, Craen M, et al. | Developing and Evaluating Rare Disease Educational Materials Co-Created by Expert Clinicians and Patients: The Paradigm of Congenital Hypogonadotropic Hypogonadism | 2017 |
| **921** | 5 | Wu CC, Econs MJ, DiMeglio LA, Insogna, KL, Levine MA, Orchard PJ, et al. | Diagnosis and Management of Osteopetrosis: Consensus Guidelines from the Osteopetrosis Working Group | 2017 |
| **922** | 5 | Rapkin BD, Garcia I, Michael W, Zhang J, Schwartz CE | Distinguishing Appraisal and Personality Influences on Quality of Life in Chronic Illness: Introducing the Quality-of-Life Appraisal Profile Version 2 | 2017 |
| **923** | 5 | Potter BK, Hutton B, Clifford TJ, Pallone N, Smith M, Stockler S, et al. | Establishing Core Outcome Sets for Phenylketonuria (Pku) and Medium-Chain Acyl-Coa Dehydrogenase (Mcad) Deficiency in Children: Study Protocol for Systematic Reviews and Delphi Surveys | 2017 |
| **924** | 5 | Paviglianiti A, Ruggeri A, Volt F, Sanz G, Milpied N, Furst S, et al. | Evaluation of a Disease Risk Index for Adult Patients Undergoing Umbilical Cord Blood Transplantation for Haematological Malignancies | 2017 |
| **925** | 5 | Rezende SM, Rodrigues SH, Brito KN, da Silva DL, Santo ML, Simões BJ, et al. | Evaluation of a Web-Based Registry of Inherited Bleeding Disorders: A Descriptive Study of the Brazilian Experience with Hemovidaweb Coagulopatias | 2017 |
| **926** | 5 | Krischer J, Cronholm PF, Burroughs C, McAlear CA, Borchin R, Easley E, Davis T, et al. | Experience with Direct-to-Patient Recruitment for Enrollment into a Clinical Trial in a Rare Disease: A Web-Based Study | 2017 |
| **927** | 5 | Mateus HE, Pérez AM, Mesa ML, Escobar G, Valvez JM, Montano JI, et al. | A First Description of the Colombian National Registry for Rare Diseases | 2017 |
| **928** | 5 | Singh JA, Shah N, Green C | Individualized Patient Decision-Aid for Immunosuppressive Drugs in Women with Lupus Nephritis: Study Protocol of a Randomized, Controlled Trial | 2017 |
| **929** | 5 | Benjamin K, Vernon MK, Patrick DL, Perfetto E, Nestler-Parr S, Burke L | Patient-Reported Outcome and Observer-Reported Outcome Assessment in Rare Disease Clinical Trials: An Ispor Coa Emerging Good Practices Task Force Report | 2017 |
| **930** | 5 | Maeda K, Kaneko M, Narukawa M, Arato T | Points to Consider: Efficacy and Safety Evaluations in the Clinical Development of Ultra-Orphan Drugs | 2017 |
| **931** | 5 | Gagete E, Delazari Dos Santos L, Gomes de Pontes L, Morato Castro F | Who Has Anaphylaxis in Brazil? Validation of a Questionnaire for Population Studies | 2017 |
| **932** | 5 | Gibson JF, Huang J, Liu KJ, Carlson KR, Foss F, Choi J, et al. | Cutaneous T-Cell Lymphoma (Ctcl): Current Practices in Blood Assessment and the Utility of T-Cell Receptor (Tcr)-Vbeta Chain Restriction | 2016 |
| **933** | 5 | Pelentsov LJ, Fielder AL, Laws TA, Esterman AJ | Development of the Parental Needs Scale for Rare Diseases: A Tool for Measuring the Supportive Care Needs of Parents Caring for a Child with a Rare Disease | 2016 |
| **934** | 5 | Porto G, Brissot P, Swinkels DW, et al. | Emqn Best Practice Guidelines for the Molecular Genetic Diagnosis of Hereditary Hemochromatosis (Hh) | 2016 |
| **935** | 5 | Rubbini M | The European Network of Coloproctology: A Strategy Towards the European Research and Healthcare System | 2016 |
| **936** | 5 | Balbi B, Vallese D, Chavannes N, Stallberg B, Baiardi P | General Practitioners and Rare Lung Diseases: A Task Force for the Development of Rare Lung Diseases Educational Material | 2016 |
| **937** | 5 | Iskrov G, Miteva-Katrandzhieva T, Stefanov R. | Multi-Criteria Decision Analysis for Assessment and Appraisal of Orphan Drugs | 2016 |
| **938** | 5 | Coler-Reilly AL, Yagishita N, Suzuki H, Sato T, Aray N, Lnoue E, et al. | Nation-Wide Epidemiological Study of Japanese Patients with Rare Viral Myelopathy Using Novel Registration System (Ham-Net) | 2016 |
| **939** | 5 | Edwards PC, Graham J, Oling R, Frantz KE | The Patient Educator Presentation in Dental Education: Reinforcing the Importance of Learning About Rare Conditions | 2016 |
| **940** | 5 | Miller PA, Mulla SM, Adams-Webber T, Sivji Y, Guyatt GH, Johnston BC | Patient-Reported Outcomes in Rare Lysosomal Storage Diseases: Key Informant Interviews and a Systematic Review Protocol | 2016 |
| **941** | 5 | van der Heijden L, Piner SR, van de Sande MA | Pigmented Villonodular Synovitis: A Crowdsourcing Study of Two Hundred and Seventy Two Patients | 2016 |
| **942** | 5 | Dell SD, Leigh MW, Lucas JS, Ferkol TW, Knowles MR, Alpern A, et al. | Primary Ciliary Dyskinesia: First Health-Related Quality-of-Life Measures for Pediatric Patients | 2016 |
| **943** | 5 | Coi A, Santoro M, Villaverde-Hueso A, Lipucci Di Paola M, Gainotti S, Taruscio D, et al. | The Quality of Rare Disease Registries: Evaluation and Characterization | 2016 |
| **944** | 5 | Paściak M, Dacko W, Sikora J, Gurlaga D, Pawlik K, Miękisiak G, et al. | Creation of an in-House Matrix-Assisted Laser Desorption Ionization-Time of Flight Mass Spectrometry Corynebacterineae Database Overcomes Difficulties in Identification of Nocardia Farcinica Clinical Isolates | 2015 |
| **945** | 5 | Watanabe Y, Sumida W, Takasu H, Oshima K, Kanamori Y, Uchida K, et al. | Early Jejunostomy Creation in Cases of Isolated Hypoganglionosis: Verification of Our Own Experience Based on a National Survey | 2015 |
| **946** | 5 | Larrandaburu M, Matte U, Noble A, Olivera Z, Sanseverino MT, Nacul L, et al. | Ethics, Genetics and Public Policies in Uruguay: Newborn and Infant Screening as a Paradigm | 2015 |
| **947** | 5 | Berlage S, Grüßner S, Lack N, Franz HB | Geross (German Obstetric Surveillance System). A Project to Improve the Treatment of Obstetric Rare Diseases and Complications Using a Web Based Documentation and Information Platform | 2015 |
| **948** | 5 | Santoro M, Coi A, Lipucci Di Paola M, Bianucci AM, Gainotti S, Mollo E, et al. | Rare Disease Registries Classification and Characterization: A Data Mining Approach | 2015 |
| **949** | 5 | van der Meijden JC, Güngör D, Kruijshaar ME, Muir AD, Broekgaarden HA, van der Ploeg AT | Ten Years of the International Pompe Survey: Patient Reported Outcomes as a Reliable Tool for Studying Treated and Untreated Children and Adults with Non-Classic Pompe Disease | 2015 |
| **950** | 5 | Schmiedeke E, de Blaauw I, Lacher M, Grasshoff-Derr S, Garcia-Vazquez A, Giuliani S, et al. | Towards the Perfect Arm Center: The European Union’s Criteria for Centers of Expertise and Their Implementation in the Member States. A Report from the Arm-Net | 2015 |
| **951** | 5 | Hubert K, Sideridis G, Sherlock R, Queally J, Rosoklija I, Kringle G, et al. | Validation of a Bowel Dysfunction Instrument for Adolescents with Spina Bifida | 2015 |
| **952** | 5 | Silberstein E, Pagkalos VA, Landau D, Berezovsky AB, Krieger Y, Shoham Y, et al. | Aplasia Cutis Congenita: Clinical Management and a New Classification System | 2014 |
| **953** | 5 | Stefanov R, Miteva-Katrandjieva Ts, Iskrov G, Damyanov D, Korukov B, Kermedchiev M, et al. | Epidemiological Registry of Neuroendocrine Tumors in Bulgaria--a Pilot Survey | 2014 |
| **954** | 5 | Sievers C, Akmatov MK, Kreienbrock L, Hille K, Ahrens W, Günther K, et al. | Evaluation of a Questionnaire to Assess Selected Infectious Diseases and Their Risk Factors : Findings of a Multicenter Study | 2014 |
| **955** | 5 | Lanzola G, Ginardi G, Russo P, Quaglini S | G-Quest: A Single Platform for Delivering Questionnaires, Educational Material, and Checklists on Mobile Devices | 2014 |
| **956** | 5 | Iczkowski KA, Egevad L, Ma J, Harding-Jackson N, Algaba F, Billis A, et al. | Intraductal Carcinoma of the Prostate: Interobserver Reproducibility Survey of 39 Urologic Pathologists | 2014 |
| **957** | 5 | Poon JL, Doctor JN, Nichol MB | Longitudinal Changes in Health-Related Quality of Life for Chronic Diseases: An Example in Hemophilia A | 2014 |
| **958** | 5 | Mazzucato M, Visonà Dalla Pozza L, Manea S, Minichiello C, Facchin P | A Population-Based Registry as a Source of Health Indicators for Rare Diseases: The Ten-Year Experience of the Veneto Region's Rare Diseases Registry | 2014 |
| **959** | 5 | Schumacher KR, Stringer KA, Donohue JE, et al. | Social Media Methods for Studying Rare Diseases | 2014 |
| **960** | 5 | Dajer-Fadel WL, Argüero-Sánchez R, Ibarra-Pérez C, Navarro-Reynoso FP | Systematic Review of Spontaneous Pneumomediastinum: A Survey of 22 Years' Data | 2014 |
| **961** | 5 | Hayashi M | Calciphylaxis: Diagnosis and Clinical Features | 2013 |
| **962** | 5 | Sparks R | Challenges in Designing a Disease Surveillance Plan: What We Have and What We Need? | 2013 |
| **963** | 5 | Klug GM, Wand H, Simpson M, Boyd A, Law M, Masters C et al. | Intensity of Human Prion Disease Surveillance Predicts Observed Disease Incidence | 2013 |
| **964** | 5 | Thompson AG, Lowe J, Fox Z, Lukic A, Porter MC, Ford L, et al. | The Medical Research Council Prion Disease Rating Scale: A New Outcome Measure for Prion Disease Therapeutic Trials Developed and Validated Using Systematic Observational Studies | 2013 |
| **965** | 5 | Conway M, Dowling JN, Chapman WW | Using Chief Complaints for Syndromic Surveillance: A Review of Chief Complaint Based Classifiers in North America | 2013 |
| **966** | 5 | Linertová R, Serrano-Aguilar P, Posada-de-la-Paz M, Hens-Pérez M, Kanavos P, Taruscio D, et al. | Delphi Approach to Select Rare Diseases for a European Representative Survey. The Burqol-Rd Study | 2012 |
| **967** | 5 | Ohkubo H, Iida H, Takahashi H, et al. | An Epidemiologic Survey of Chronic Intestinal Pseudo-Obstruction and Evaluation of the Newly Proposed Diagnostic Criteria | 2012 |
| **968** | 5 | Kunisawa S, Ikai H, Imanaka Y | Incidence and Prevention of Postoperative Venous Thromboembolism: Are They Meaningful Quality Indicators in Japanese Health Care Settings? | 2012 |
| **969** | 5 | Kessel M, Hannemann-Weber H, Kratzer J | Innovative Work Behavior in Healthcare: The Benefit of Operational Guidelines in the Treatment of Rare Diseases | 2012 |
| **970** | 5 | Petrossians P, Tichomirowa MA, Stevenaert A, Martin D, Daly AF, Beckers A | The Liege Acromegaly Survey (Las): A New Software Tool for the Study of Acromegaly | 2012 |
| **971** | 5 | Arnaud L, Devilliers H, Peng SL, Mathian A, Costedoat-Chalumeau N, Buckner J, et al. | The Relapsing Polychondritis Disease Activity Index: Development of a Disease Activity Score for Relapsing Polychondritis | 2012 |
| **972** | 5 | Hansson E, Svensson H, Brorson H | Review of Dercum's Disease and Proposal of Diagnostic Criteria, Diagnostic Methods, Classification and Management | 2012 |
| **973** | 5 | Knowles RL, Friend H, Lynn R, Mitchell S, Michie C, Ihekweazu C | Surveillance of Rare Diseases: A Public Health Evaluation of the British Paediatric Surveillance Unit | 2012 |
| **974** | 5 | Ranganath L, Taylor AM, Shenkin A, Fraser WD, Jarvis J, Gallagher JA, et al. | Identification of Alkaptonuria in the General Population: A United Kingdom Experience Describing the Challenges, Possible Solutions and Persistent Barriers | 2011 |
| **975** | 5 | Clarke JT, Giugliani R, Sunder-Plassmann G, Elliott PM, Pintos-Morell G, Hernberg-Ståhl E, et al. | Impact of Measures to Enhance the Value of Observational Surveys in Rare Diseases: The Fabry Outcome Survey (Fos) | 2011 |
| **976** | 5 | Hannemann-Weber H, Kessel M, Budych K, Schultz C | Shared Communication Processes within Healthcare Teams for Rare Diseases and Their Influence on Healthcare Professionals' Innovative Behavior and Patient Satisfaction | 2011 |
| **977** | 5 | Martins AM, Kerstenezky M, Linares A, Politei J, Kohan R, Ospina S, et al. | Utility of Rare Disease Registries in Latin America | 2011 |
| **978** | 5 | Censi F, Falbo V, Floridia G, Salvatore M, Tosto F, De Rosa M, et al. | The Italian External Quality Control Program for Familial Adenomatous Polyposis of the Colon: Five Years of Experience | 2010 |
| **979** | 5 | He S, Zurynski YA, Elliott EJ | Evaluation of a National Resource to Identify and Study Rare Diseases: The Australian Paediatric Surveillance Unit | 2009 |
| **980** | 5 | Brown ML, Cedeño AR, Edell ES, Hagler DJ, Schaff HV | Operative Strategies for Pulmonary Artery Occlusion Secondary to Mediastinal Fibrosis | 2009 |
| **981** | 5 | Nan B, Lin X | Analysis of Case-Control Age-at-Onset Data Using a Modified Case-Cohort Method | 2008 |
| **982** | 5 | Knol MJ, Vandenbroucke JP, Scott P, Egger M | What Do Case-Control Studies Estimate? Survey of Methods and Assumptions in Published Case-Control Research | 2008 |
| **983** | 5 | Mojica P, Smith D, Ellenhorn J | Adjuvant Radiation Therapy Is Associated with Improved Survival for Gallbladder Carcinoma with Regional Metastatic Disease | 2007 |
| **984** | 5 | Aymé S, Schmidtke J | Networking for Rare Diseases: A Necessity for Europe | 2007 |
| **985** | 5 | Takata S, Hashimoto J, Nakatsuka K, et al. | Guidelines for Diagnosis and Management of Paget's Disease of Bone in Japan | 2006 |
| **986** | 5 | Yetter Read C | Conducting a Client-Focused Survey Using E-Mail | 2004 |
| **987** | 5 | Ben-Abraham R, Greenfeld A, Rozenman J, Ben-Dov I. | Pulmonary Alveolar Proteinosis: Step-by-Step Perioperative Care of Whole Lung Lavage Procedure | 2002 |
| **988** | 5 | Doherty JA, Grenier D | The Canadian Paediatric Surveillance Program: Beyond Collecting Numbers | 2001 |
| **989** | 5 | Elliott EJ, Nicoll A, Lynn R, Marchessault V, Hirasing R, Ridley G | Rare Disease Surveillance: An International Perspective | 2001 |
| **990** | 5 | Silva MC, Coutinho P, Pinheiro CD, Neves JM, Serrano P | Hereditary Ataxias and Spastic Paraplegias: Methodological Aspects of a Prevalence Study in Portugal | 1997 |
| **991** | 6 | Yoon HY, Kim JH, Kim YJ, Song JW | Pulmonary Alveolar Proteinosis in Korea: Analysis of Prevalence and Incidence Via a Nationwide Population-Based Study | 2020 |
| **992** | 6 | Willey C, Kamat S, Stellhorn R, Blais J | Analysis of Nationwide Data to Determine the Incidence and Diagnosed Prevalence of Autosomal Dominant Polycystic Kidney Disease in the USA: 2013-2015 | 2019 |
| **993** | 6 | Jo A, Larson S, Carek P, Peabody MR, Peterson LE, Mainous AG | Prevalence and Practice for Rare Diseases in Primary Care: A National Cross-Sectional Study in the USA | 2019 |
| **994** | 6 | Grier J, Hirano M, Karaa A, Shepard E, Thompson JLP | Diagnostic Odyssey of Patients with Mitochondrial Disease Results of a Survey | 2018 |
| **995** | 6 | Blencowe H, Moorthie S, Petrou M, Hamamy H, Povey S, Bittles A, et al | Rare Single Gene Disorders: Estimating Baseline Prevalence and Outcomes Worldwide | 2018 |
| **996** | 6 | Shah MV, Hook CC, Call TG, Go RS | A Population-Based Study of Large Granular Lymphocyte Leukemia | 2016 |
| **997** | 6 | Willis JR, Vitale S, Morse L, Parke DW 2nd, Rich WL, Lum F, et al. | The Prevalence of Myopic Choroidal Neovascularization in the United States: Analysis of the Iris® Data Registry and Nhanes | 2016 |
| **998** | 6 | Lee SO, Yu SY | Utilization Patterns of Coagulation Factor Consumption for Patients with Hemophilia | 2016 |
| **999** | 6 | Papa R, Cant A, Klein C, Little MA, Wulffraat NM, Gattorno M, et al | Towards European Harmonisation of Healthcare for Patients with Rare Immune Disorders: Outcome from the Ern Rita Registries Survey | 2020 |
| **1000** | 6 | Mehta BY, Ibrahim S, Briggs W, Efthimiou P | Racial/Ethnic Variations in Morbidity and Mortality in Adult Onset Still's Disease: An Analysis of National Dataset | 2019 |
| **1001** | 6 | Mehta BY, Ibrahim S, Briggs W, Efthimiou P | Sleep-Disordered Breathing in Children with Rare Skeletal Disorders: A Survey of Clinical Records | 2018 |
| **1002** | 6 | Weissler JM, Shubinets V, Carney MJ, Low DW | Complex Truncal Masses in the Setting of Cloves Syndrome: Aesthetic and Functional Implications | 2017 |
| **1003** | 6 | Kedia S, Ahuja V | Epidemiology of Inflammatory Bowel Disease in India: The Great Shift East | 2017 |
| **1004** | 6 | Sanchorawala V, McCausland KL, White MK, Bayliss MS, Guthrie SD, Lo S, Skinner M | A Longitudinal Evaluation of Health-Related Quality of Life in Patients with Al Amyloidosis: Associations with Health Outcomes over Time | 2017 |
| **1005** | 6 | Horick NK, Manful A, Lowery J, Domchek S, Moorman P, Griffin C, et al. | Physical and Psychological Health in Rare Cancer Survivors | 2017 |
| **1006** | 6 | Suzuki S, Midorikawa S, Matsuzuka T, Fukushima T, Ito Y, Shimura H, Takahashi H, et al. | Prevalence and Characterization of Thyroid Hemiagenesis in Japan: The Fukushima Health Management Survey | 2017 |
| **1007** | 6 | Imtiaz S, Nasir K, Drohlia MF, Salman B, Ahmad A | Frequency of Kidney Diseases and Clinical Indications of Pediatric Renal Biopsy: A Single Center Experience | 2016 |
| **1008** | 6 | Anderzén-Carlsson A | Charge Syndrome-a Five Case Study of the Syndrome Characteristics and Health Care Consumption During the First Year in Life | 2015 |
| **1009** | 6 | Kim HS, Lyoo CH, Lee PH, Kim SJ, Park MY, Ma HI, et al. | Current Status of Huntington's Disease in Korea: A Nationwide Survey and National Registry Analysis | 2015 |
| **1010** | 6 | Litchfield K, Thomsen H, Mitchell JS, Sundquist J, Houlston RS, Hemminki K, et al. | Quantifying the Heritability of Testicular Germ Cell Tumour Using Both Population-Based and Genomic Approaches | 2015 |
| **1011** | 6 | Simeón-Aznar CP, Fonollosa-Plá V, Tolosa-Vilella C, Espinosa-Garriga G, Campillo-Grau M, Ramos-Casals M, et al. | Registry of the Spanish Network for Systemic Sclerosis: Survival, Prognostic Factors, and Causes of Death | 2015 |
| **1012** | 6 | Kamath S, Arkader A, Jubran RF | Outcomes of Children Younger Than 24 Months with Langerhans Cell Histiocytosis and Bone Involvement: A Report from a Single Institution | 2014 |
| **1013** | 6 | Latino GA, Brown D, Glazier RH, Weyman JT, Faughnan ME | Targetig under-Diagnosis in Hereditary Hemorrhagic Telangiectasia: A Model Approach for Rare Diseases? | 2014 |
| **1014** | 6 | Wixner J, Mundayat R, Karayal ON, Anan I, Karling P, Suhr OB | Thaos: Gastrointestinal Manifestations of Transthyretin Amyloidosis - Common Complications of a Rare Disease | 2014 |
| **1015** | 6 | Chung SE, Cheong HK, Park JH, Kim HJ | Burden of Disease of Multiple Sclerosis in Korea | 2012 |
| **1016** | 6 | Trama A, Mallone S, Ferretti S, Meduri F, Capocaccia R, Gatta G | The Burden of Rare Cancers in Italy: The Surveillance of Rare Cancers in Italy (Rita) Project | 2012 |
| **1017** | 6 | Nakamura C, Bromberg M, Bhargava S, Wicks P, Zeng-Treitler Q | Mining Online Social Network Data for Biomedical Research: A Comparison of Clinicians' and Patients' Perceptions About Amyotrophic Lateral Sclerosis Treatments | 2012 |
| **1018** | 6 | Risselada R, de Vries LM, Dippel DW, et al. | Incidence, Treatment, and Case-Fatality of Non-Traumatic Subarachnoid Haemorrhage in the Netherlands | 2011 |
| **1019** | 6 | Lefton-Greif MA, Crawford TO, McGrath-Morrow S, Carson KA, Lederman HM. | Safety and Caregiver Satisfaction with Gastrostomy in Patients with Ataxia Telangiectasia | 2011 |
| **1020** | 6 | Yang WC, Wu HP | Clinical Analysis of Hypertension in Children Admitted to the Emergency Department | 2010 |
| **1021** | 6 | Verbeek WH, Van De Water JM, Al-Toma A, Oudejans JJ, Mulder CJ, Coupé VM | Incidence of Enteropathy - Associated T-Cell Lymphoma: A Nation-Wide Study of a Population-Based Registry in the Netherlands | 2008 |
| **1022** | 6 | Kodra Y, Morosini PR, Petrigliano R, Agazio E, Salerno P, Taruscio D | Access to and Quality of Health and Social Care for Rare Diseases: Patients' and Caregivers' Experiences | 2007 |
| **1023** | 6 | Pourhassan S, Grotemeyer D, Fokou M, et al. | Extracranial Carotid Arteries Aneurysms in Children. Single-Center Experiences in 4 Patients and Review of the Literature | 2007 |
| **1024** | 6 | Taheri-Kadkhoda Z, Björk-Eriksson T, Johansson KA, Mercke C | Long-Term Treatment Results for Nasopharyngeal Carcinoma: The Sahlgrenska University Hospital Experience | 2007 |
| **1025** | 6 | Bidstrup C, Andersen PH, Skinhøj P, Andersen AB | Tuberculous Meningitis in a Country with a Low Incidence of Tuberculosis: Still a Serious Disease and a Diagnostic Challenge | 2002 |
| **1026** | 6 | Guidetti D, Sabadini R, Ferlini A, Torrente I | Epidemiological Survey of X-Linked Bulbar and Spinal Muscular Atrophy, or Kennedy Disease, in the Province of Reggio Emilia, Italy | 2001 |
| **1027** | 6 | Appelbaum L, Yigla M, Bendayan D, et al. | Primary Pulmonary Hypertension in Israel: A National Survey | 2001 |
| **1028** | 7 | Grau C, Jakobsen MH, Harbo G, Svane-Knudsen V, Wedervang K, Larsen S, et al. | Sino-Nasal Cancer in Denmark 1982-1991: A Nationwide Survey | 2001 |
| **1029** | 7 | Schlimok G | Adjuvant and Palliative Immunotherapy of Colorectal Carcinoma | 1997 |
| **1030** | 7 | Schilling T, Ziegler R | Current Therapy of Hypoparathyroidism - a Survey of German Endocrinology Centers | 1997 |
| **1031** | 7 | Boccon-Gibod LA, Krichen HA, Carlier-Mercier LM, Salaun JF, Fontaine JL, Leverger GR | Digestive Tract Involvement in Langerhans Cell Histiocytosis | 1996 |
| **1032** | 7 | Simpson DM | Improving the Reporting of Notifiable Diseases in Texas: Suggestions from an Ad Hoc Committee of Providers | 1996 |
| **1033** | 7 | Trimble EL, Rubinstein LV, Menck HR, Hankey BF, Kosary C, Giusti RM | Vaginal Clear Cell Adenocarcinoma in the United States | 1996 |
| **1034** | 7 | Rozan R, Albuisson E, Giraud B, Donnarieix D, Delannes M, Pigneux J, et al. | Interstitial Brachytherapy for Penile Carcinoma: A Multicentric Survey (259 Patients) | 1995 |
| **1035** | 7 | Spickett GP, Askew T, Chapel HM | Management of Primary Antibody Deficiency by Consultant Immunologists in the United Kingdom: A Paradigm for Other Rare Diseases | 1995 |
| **1036** | 7 | Maheux B, Delorme P, Béland F, Beaudry J | Humanism in Medical Education: A Study of Educational Needs Perceived by Trainees of Three Canadian Schools | 1990 |
| **1037** | 7 | Ritvo ER, Mason-Brothers A, Freeman BJ, Pingree C, Jenson WR, McMahon WM, et al. | The Ucla-University of Utah Epidemiologic Survey of Autism: The Etiologic Role of Rare Diseases | 1990 |
| **1038** | 7 | Bortin MM, Rimm AA | Increasing Utilization of Bone Marrow Transplantation. Ii. Results of the 1985-1987 Survey | 1989 |
| **1039** | 7 | Pearson M | Social Factors and Leprosy in Lamjung, West Central Nepal: Implication for Disease Control | 1982 |
| **1040** | 7 | Moodie JW, MacKenzie DJ, Kipps A | Subacute Sclerosing Panencephalitis (Sspe) in Southern Africa. Recent Additions to the Sspe Registry | 1980 |
| **1041** | 7 | Arthes FG, Masi AT | Myocardial Infarction in Younger Women. Associated Clinical Features and Relationship to Use of Oral Contraceptive Drugs | 1976 |
| **1042** | 7 | Hamlyn AN, Sherlock S | The Epidemiology of Primary Biliary Cirrhosis: A Survey of Mortality in England and Wales | 1974 |
| **1043** | 8 | Hanisch M, Wiemann S, Bohner L, Jung S, Kleinheinz J. | State of Knowledge About Information Sources and Health Care Centres for Rare Diseases among Affected People in Germany | 2020 |
| **1044** | 8 | McLawhorn, J,Balamurugan A, Gardner JM, Kaley JR, George MJ | Using Social Media to Assess Mental Health and Quality of Care among Patients with Darier Disease: A Novel Resource for Patient Advocacy | 2019 |
| **1045** | 8 | Bösch A, Wager J, Zernikow B, Thalemann R, Frenzel H, Krude H, et al. | Life-Limiting Conditions at a University Pediatric Tertiary Care Center: A Cross-Sectional Study | 2018 |
| **1046** | 8 | Dekker BL, Newbold KL, Führer D, Waguespack SG, Handkiewicz-Junak D, Links TP | Survey on Paediatric Differentiated Thyroid Cancer Care in Europe | 2018 |
| **1047** | 8 | Lanzarini E, Parmeggiani A | Disability and Inclusive Education in an Italian Region: Analysis of the Data for the School Year 2012-2013 | 2017 |

**References**

1. Rudebeck M, Scott C, Sireau N, Ranganath L. A patient survey on the impact of alkaptonuria symptoms as perceived by the patients and their experiences of receiving diagnosis and care. JIMD Rep [Internet]. 2020/05/13. 2020;53:71–9. Available from: https://www.ncbi.nlm.nih.gov/pmc/articles/PMC7203644/pdf/JMD2-53-71.pdf
2. Mancuso M, Filosto M, Lamperti C, Musumeci O, Santorelli FM, Servidei S, et al. Awareness of rare and genetic neurological diseases among italian neurologist. A national survey. Neurol Sci [Internet]. 2020; Available from: http://www.embase.com/search/results?subaction=viewrecord&from=export&id=L2004147496
3. Takeuchi F, Nakamura H, Yonemoto N, Komaki H, Rosales RL, Kornberg AJ, et al. Clinical practice with steroid therapy for Duchenne muscular dystrophy: An expert survey in Asia and Oceania. Brain Dev [Internet]. 2020;42:277–88. Available from: http://www.embase.com/search/results?subaction=viewrecord&from=export&id=L2004670732
4. Yan X, He S, Dong D. Determining how far an adult rare disease patient needs to travel for a definitive diagnosis: A cross-sectional examination of the 2018 national rare disease survey in China. Int J Environ Res Public Health [Internet]. 2020;17. Available from: http://www.embase.com/search/results?subaction=viewrecord&from=export&id=L2003965694
5. Ramalle-Gómara E, Domínguez-Garrido E, Gómez-Eguílaz M, Marzo-Sola ME, Ramón-Trapero JL, Gil-De-Gómez J. Education and information needs for physicians about rare diseases in Spain. Orphanet J Rare Dis [Internet]. 2020;15. Available from: http://www.embase.com/search/results?subaction=viewrecord&from=export&id=L630625190
6. Hartman AL, Hechtelt Jonker A, Parisi MA, Julkowska D, Lockhart N, Isasi R. Ethical, legal, and social issues (ELSI) in rare diseases: a landscape analysis from funders. Eur J Hum Genet [Internet]. 2020;28:174–81. Available from: http://www.embase.com/search/results?subaction=viewrecord&from=export&id=L2003455483
7. Requena-Fernández MÁ, Dasí F, Castillo S, Barajas-Cenobi R, Navarro-García MM, Escribano A. Knowledge of rare respiratory diseases among paediatricians and medical school students. J Clin Med [Internet]. 2020;9. Available from: http://www.embase.com/search/results?subaction=viewrecord&from=export&id=L2004064123
8. Painous C, van Os N, Delamarre A, Michailoviene I, Marti MJ, van de Warrenburg BP, et al. Management of rare movement disorders in Europe: outcome of surveys of the European Reference Network for Rare Neurological Diseases. Eur J Neurol [Internet]. 2020; Available from: http://www.embase.com/search/results?subaction=viewrecord&from=export&id=L631700018
9. Peeks F, Boonstra WF, de Baere L, Carøe C, Casswall T, Cohen D, et al. Research priorities for liver glycogen storage disease: An international priority setting partnership with the James Lind Alliance. J Inherit Metab Dis [Internet]. 2020;43:279–89. Available from: http://www.embase.com/search/results?subaction=viewrecord&from=export&id=L2003606155
10. Byrne N, Turner J, Marron R, Lambert DM, Murphy DN, O’Sullivan G, et al. The role of primary care in management of rare diseases in Ireland. Ir J Med Sci [Internet]. 2020; Available from: http://www.embase.com/search/results?subaction=viewrecord&from=export&id=L2004087065
11. Quinn L, Davis K, Yee A, Snyder H. Understanding genetic learning needs of people affected by rare disease. J Genet Couns [Internet]. 2020; Available from: http://www.embase.com/search/results?subaction=viewrecord&from=export&id=L631156786
12. Qian E, Thong MK, Flodman P, Gargus J. A comparative study of patients’ perceptions of genetic and genomic medicine services in California and Malaysia. J Community Genet. 2019;10.
13. Marques-da-Silva D, Francisco R, dos Reis Ferreira V, Forbat L, Lagoa R, Videira PA, et al. An electronic questionnaire for liver assessment in congenital disorders of glycosylation (LeQCDG): A patient-centered study. JIMD Rep. 2019.
14. Conner T, Cook F, Fernandez V, Rascati K, Rangel-Miller V. An online survey on burden of illness among families with post-stem cell transplant mucopolysaccharidosis type i children in the United States. Orphanet J Rare Dis. 2019;14.
15. Khair K, Pelentsov L. Assessing the supportive care needs of parents with a child with a bleeding disorder using the Parental Needs Scale for Rare Diseases (PNS-RD): A single-centre pilot study. Haemophilia. 2019;25:831–7.
16. Noone D, Skouw-Rasmussen N, Lavin M, van Galen KPM, Kadir RA. Barriers and challenges faced by women with congenital bleeding disorders in Europe: Results of a patient survey conducted by the European Haemophilia Consortium. Haemophilia. 2019;25.
17. Rush ET, Moseley S, Petryk A. Burden of disease in pediatric patients with hypophosphatasia: results from the HPP Impact Patient Survey and the HPP Outcomes Study Telephone interview. Orphanet J Rare Dis [Internet]. BioMed Central; 2019;14:N.PAG-N.PAG. Available from: http://search.ebscohost.com/login.aspx?direct=true&db=rzh&AN=138108750&site=ehost-live
18. Rice DB, Cañedo-Ayala M, Carboni-Jiménez A, Carrier ME, Cumin J, Malcarne VL, et al. Challenges and support service preferences of informal caregivers of people with systemic sclerosis: a cross-sectional survey. Disabil Rehabil. 2020;42.
19. Crowe AL, McKnight AJ, McAneney H. Communication Needs for Individuals With Rare Diseases Within and Around the Healthcare System of Northern Ireland. Front Public Heal. 2019;7.
20. Hollis A, Dart A, Morgan C, Mammen C, Zappitelli M, Chanchlani R, et al. Delays in diagnosis of nephrotic syndrome in children: A survey study. Paediatr Child Heal. 2019;24.
21. Baldelli I, Gallo F, Crimi M, Fregatti P, Mellini L, Santi P, et al. Experiences of patients with Poland syndrome of diagnosis and care in Italy: A pilot survey. Orphanet J Rare Dis. 2019;14.
22. Yankouskaya A, Boughey A, McCagh J, Neal A, de Bezenac C, Davies SJ. Illness Perception Mediates the Relationship Between the Severity of Symptoms and Perceived Health Status in Patients With Behçet Disease. J Clin Rheumatol [Internet]. Baltimore, Maryland: Lippincott Williams & Wilkins; 2019;25:319–24. Available from: http://search.ebscohost.com/login.aspx?direct=true&db=rzh&AN=140139637&site=ehost-live
23. Swezey T, Reeve BB, Hart TS, Floor MK, Dollar CM, Gillies AP, et al. Incorporating the patient perspective in the study of rare bone disease: insights from the osteogenesis imperfecta community. Osteoporos Int [Internet]. 2018/09/08. 2019;30:507–11. Available from: https://www.ncbi.nlm.nih.gov/pmc/articles/PMC6449303/pdf/198_2018_Article_4690.pdf
24. Nakahara Y, Kitoh H, Nakashima Y, Toguchida J, Haga N. Longitudinal study of the activities of daily living and quality of life in Japanese patients with fibrodysplasia ossificans progressiva. Disabil Rehabil. 2019;41.
25. Morrison A, Oussoren E, Friedel T, Cruz J, Yilmaz N. Pathway to diagnosis and burden of illness in mucopolysaccharidosis type VII- A European caregiver survey. Orphanet J Rare Dis. 2019;14.
26. Karaa A, Goldstein A, Balcells C, Mann K, Stanley L, Yeske PE, et al. Primary mitochondrial disease in the US: Data from patients and physicians’ perspective on health care delivery. Data Br. 2019;25.
27. Courbier S, Dimond R, Bros-Facer V. Share and protect our health data: An evidence based approach to rare disease patients’ perspectives on data sharing and data protection - Quantitative survey and recommendations. Orphanet J. Rare Dis. 2019.
28. Yoo J, Halley MC, Lown EA, Yank V, Ort K, Cowan MJ, et al. Supporting caregivers during hematopoietic cell transplantation for children with primary immunodeficiency disorders. J Allergy Clin Immunol [Internet]. 2019;143:2271–8. Available from: http://www.embase.com/search/results?subaction=viewrecord&from=export&id=L2001326335
29. Marshall DA, MacDonald K V., Heidenreich S, Hartley T, Bernier FP, Gillespie MK, et al. The value of diagnostic testing for parents of children with rare genetic diseases. Genet Med. 2019;21.
30. Delisle VC, Gumuchian ST, El-Baalbaki G, Körner A, Malcarne VL, Peláez S, et al. Training and support needs of scleroderma support group facilitators: the North American Scleroderma Support Group Facilitators Survey. Disabil Rehabil. 2019;41.
31. López-Bastida J, Ramos-Goñi JM, Aranda-Reneo I, Trapero-Bertran M, Kanavos P, Rodriguez Martin B. Using a stated preference discrete choice experiment to assess societal value from the perspective of decision-makers in Europe. Does it work for rare diseases? Health Policy (New York). 2019;123.
32. López-Bastida J, Ramos-Goñi JM, Aranda-Reneo I, Taruscio D, Magrelli A, Kanavos P. Using a stated preference discrete choice experiment to assess societal value from the perspective of patients with rare diseases in Italy. Orphanet J Rare Dis. 2019;14.
33. Baqué M, Colineaux H, Dreyfus I, Mesthé P, Mazereeuw-Hautier J. Why is it so difficult for GPs to effectively manage patients with rare skin diseases? Press Medicale. 2019;48.
34. Ivarsson B, Hesselstrand R, Rådegran G, Kjellström B. Adherence and medication belief in patients with pulmonary arterial hypertension or chronic thromboembolic pulmonary hypertension: A nationwide population-based cohort survey. Clin Respir J. 2018;12.
35. Fu L, Kanani A, Lacuesta G, Waserman S, Betschel S. Canadian physician survey on the medical management of hereditary angioedema. Ann Allergy, Asthma Immunol. 2018;121.
36. Peiris V, Xu K, Agler HL, Chen EA, Gopal-Srivastava R, Lappin BM, et al. Children and adults with rare diseases need innovative medical devices. J Med Devices, Trans ASME. 2018;12.
37. Ho NT, Kroner B, Grinspan Z, Fureman B, Farrell K, Zhang J, et al. Comorbidities of Rare Epilepsies: Results from the Rare Epilepsy Network. J Pediatr. 2018;203.
38. Tory HO, Carrasco R, Griffin T, Huber AM, Kahn P, Robinson AB, et al. Comparing the importance of quality measurement themes in juvenile idiopathic inflammatory myositis between patients and families and healthcare professionals. Pediatr Rheumatol. 2018;16.
39. Schadewald A, Kimball E, Ou L. Coping strategies, stress, and support needs in caregivers of children with mucopolysaccharidosis. JIMD Rep. 2018.
40. Efthymiadou O, Mossman J, Kanavos P. Differentiation of health-related quality of life outcomes between five disease areas: Results from an international survey of patients. Int. J. Technol. Assess. Health Care. 2018.
41. Lopes MT, Koch VH, Sarrubbi-Junior V, Gallo PR, Carneiro-Sampaio M. Difficulties in the diagnosis and treatment of rare diseases according to the perceptions of patients, relatives and health care professionals. Clinics. 2018;73.
42. Kanavin ØJ, Fjermestad KW. Gastrointestinal and urinary complaints in adults with hereditary spastic paraparesis. Orphanet J Rare Dis. 2018;13.
43. McCausland KL, White MK, Guthrie SD, Quock T, Finkel M, Lousada I, et al. Light Chain (AL) Amyloidosis: The Journey to Diagnosis. Patient. 2018;11.
44. Fu M, Guan X, Wei G, Xin X, Shi L. Medical service utilisation, economic burden and health status of patients with rare diseases in China. J Chinese Pharm Sci. 2018;27.
45. Liu S, Adelman DT, Xu Y, Sisco J, Begelman SM, Webb SM, et al. Patient-centered assessment on disease burden, quality of life, and treatment satisfaction associated with acromegaly. J Investig Med. 2018;66.
46. Chung PH, Kampp JT, Voelzke BB. Patients’ Experiences With Extramammary Paget Disease: An Online Pilot Study Querying a Patient Support Group. Urology. 2018;111.
47. Peyron C, Pélissier A, Béjean S. Preference heterogeneity with respect to whole genome sequencing. A discrete choice experiment among parents of children with rare genetic diseases. Soc Sci Med. 2018;214.
48. Richardson E, Spinks C, Davis A, Turner C, Atherton J, McGaughran J, et al. Psychosocial Implications of Living with Catecholaminergic Polymorphic Ventricular Tachycardia in Adulthood. J Genet Couns. 2018;27.
49. Kazimierska-Zając M, Dymarek R, Rosińczuk J. Quality of Life and the Degree of Disease Acceptance In Patients with Spinocerebellar Ataxia. J Neurol Neurosurg Nurs. 2018;7:12–21.
50. Cockerell I, Guenin M, Heimdal K, Bjørnvold M, Selmer KK, Rouvière O. Renal manifestations of tuberous sclerosis complex: Patients’ and parents’ knowledge and routines for renal follow-up - A questionnaire study. BMC Nephrol. 2018;19.
51. Adachi T, Imanishi N, Ogawa Y, Furusawa Y, Izumida Y, Izumi Y, et al. Survey on patients with undiagnosed diseases in Japan: Potential patient numbers benefiting from Japan’s initiative on rare and undiagnosed diseases (IRUD). Orphanet J Rare Dis. 2018;13.
52. Ivleva A, Weith E, Mehta A, Hughes DA. The influence of patient-reported joint manifestations on quality of life in fabry patients. JIMD Rep. 2018.
53. Szczepura A, Wynn S, Searle B, Khan AJ, Palmer T, Biggerstaff D, et al. UK families with children with rare chromosome disorders: Changing experiences of diagnosis and counselling (2003-2013). Clin Genet. 2018;93.
54. Zhu X, Smith RA, Parrott RL, Worthington AK. Understanding information sharing about rare diseases: an evaluation of the NIH’s website on AATD. J Commun Healthc. 2018;11.
55. Anderson M, Elliott EJ, Zurynski YA. Australian families living with rare disease: Experiences of diagnosis, health services use and needs for psychosocial support. Orphanet J Rare Dis. 2013;8.
56. Richardson J, Iezzi A, Chen G, Maxwell A. Communal Sharing and the Provision of Low-Volume High-Cost Health Services: Results of a Survey. PharmacoEconomics - Open. 2017;1.
57. Chen CX, Baker JR, Nichol MB. Economic Burden of Illness among Persons with Hemophilia B from HUGS Vb: Examining the Association of Severity and Treatment Regimens with Costs and Annual Bleed Rates. Value Heal. 2017;20.
58. Polisena J, Burgess M, Mitton C, Lynd LD. Engaging the Canadian public on reimbursement decision-making for drugs for rare diseases: A national online survey. BMC Health Serv. Res. 2017.
59. Bogart KR, Irvin VL. Health-related quality of life among adults with diverse rare disorders. Orphanet J Rare Dis. 2017;12.
60. Patriquin CJ, Clark WF, Pavenski K, Arnold DM, Rock G, Foley SR. How we treat thrombotic thrombocytopenic purpura: Results of a Canadian TTP practice survey. J Clin Apher. 2017;32.
61. Amelot V, Bungener C, Guilmin-Crepon S, Schroedt J, Alberti C, Husson I. Preferences for Receiving Results from a Rare Disease Clinical Trial: A Survey of Subjects with Friedreich’s Ataxia and their Parents. Pharmaceut Med. 2017;31.
62. Wiss J, Levin LA, Andersson D, Tinghög G. Prioritizing Rare Diseases: Psychological Effects Influencing Medical Decision Making. Med Decis Mak. 2017;37.
63. Zurynski Y, Gonzalez A, Deverell M, Phu A, Leonard H, Christodoulou J, et al. Rare disease: A national survey of paediatricians’ experiences and needs. BMJ Paediatr Open. 2017;1.
64. Schwartz CE, Michael W, Rapkin BD. Resilience to health challenges is related to different ways of thinking: mediators of physical and emotional quality of life in a heterogeneous rare-disease cohort. Qual Life Res. 2017;26.
65. Lloyd AJ, Gallop K, Ali S, Hughes D, MacCulloch A. Social preference weights for treatments in Fabry disease in the UK: a discrete choice experiment. Curr Med Res Opin. 2017;33.
66. Bayliss M, McCausland KL, Guthrie SD, White MK. The burden of amyloid light chain amyloidosis on health-related quality of life. Orphanet J Rare Dis. 2017;12.
67. O’Hara J, Hughes D, Camp C, Burke T, Carroll L, Diego DAG. The cost of severe haemophilia in Europe: the CHESS study. Orphanet J Rare Dis. 2017;12.
68. Heuyer T, Pavan S, Vicard C. The health and life path of rare disease patients: results of the 2015 French barometer. Patient Relat Outcome Meas. 2017;Volume 8.
69. Cacioppo CN, Conway LJ, Mehta D, Krantz ID, Noon SE. Attitudes about the use of internet support groups and the impact among parents of children with Cornelia de Lange syndrome. Am J Med Genet Part C Semin Med Genet. 2016;172.
70. Engelstad K, Sklerov M, Kriger J, Sanford A, Grier J, Ash D, et al. Attitudes toward prevention of mtDNA-related diseases through oocyte mitochondrial replacement therapy. Hum Reprod. 2016;31.
71. Xin XX, Zhao L, Guan XD, Shi LW. Determinants and equity evaluation for health expenditure among patients with rare diseases in China. Chin Med J (Engl). 2016;129.
72. Behan L, Galvin AD, Rubbo B, Masefield S, Copeland F, Manion M, et al. Diagnosing primary ciliary dyskinesia: An international patient perspective. Eur Respir J. 2016;48.
73. Karaa A, Kriger J, Grier J, Holbert A, Thompson JLP, Parikh S, et al. Mitochondrial disease patients’ perception of dietary supplements’ use. Mol Genet Metab. 2016;119.
74. Serrano-Aguilar P, Trujillo-Martin MM, Del Pino Sedeño T, Pérez De La Rosa A, De Pascual y Medina AM, Perestelo-Pérez L, et al. Patient participation in the development of a clinical guideline for inherited retinal dystrophies. Expert Opin. Orphan Drugs. 2016.
75. Darquy S, Moutel G, Lapointe AS, D’Audiffret D, Champagnat J, Guerroui S, et al. Patient/family views on data sharing in rare diseases: Study in the European LeukoTreat project. Eur J Hum Genet. 2016;24.
76. Morel T, Aymé S, Cassiman D, Simoens S, Morgan M, Vandebroek M. Quantifying benefit-risk preferences for new medicines in rare disease patients and caregivers. Orphanet J Rare Dis. 2016;11.
77. Delisle VC, Gumuchian ST, Peláez S, Malcarne VL, El-Baalbaki G, Körner A, et al. Reasons for non-participation in scleroderma support groups. Clin Exp Rheumatol. 2016;34.
78. Péntek M, Gulácsi L, Brodszky V, Baji P, Boncz I, Pogány G, et al. Social/economic costs and health-related quality of life of mucopolysaccharidosis patients and their caregivers in Europe. Eur J Heal Econ. 2016;17.
79. Senger BA, Ward LD, Barbosa-Leiker C, Bindler RC. Stress and coping of parents caring for a child with mitochondrial disease. Appl Nurs Res. 2016;29.
80. Molster C, Urwin D, Di Pietro L, Fookes M, Petrie D, Van Der Laan S, et al. Survey of healthcare experiences of Australian adults living with rare diseases. Orphanet J Rare Dis. 2016;11.
81. Merkel PA, Manion M, Gopal-Srivastava R, Groft S, Jinnah HA, Robertson D, et al. The partnership of patient advocacy groups and clinical investigators in the rare diseases clinical research network. Orphanet J Rare Dis. 2016;11.
82. Carpenter DM, Thorpe CT, Alexander DS, Sage AJ, Lewis M, Hogan SL, et al. The Relationship Between Social Support, Social Constraint, and Psychological Adjustment for Patients with Rare Autoimmune Disease. Curr Rheumatol Rev. 2016;12.
83. Pelentsov LJ, Fielder AL, Laws TA, Esterman AJ. The supportive care needs of parents with a child with a rare disease: Results of an online survey. BMC Fam. Pract. 2016.
84. Bosch AM, Burlina A, Cunningham A, Bettiol E, Moreau-Stucker F, Koledova E, et al. Assessment of the impact of phenylketonuria and its treatment on quality of life of patients and parents from seven European countries. Orphanet J Rare Dis. 2015;10.
85. Dragojlovic N, Rizzardo S, Bansback N, Mitton C, Marra CA, Lynd LD. Challenges in Measuring the Societal Value of Orphan Drugs: Insights from a Canadian Stated Preference Survey. Patient. 2015.
86. Ramalle-Gõmara E, Ruiz E, Quiñones C, Andrés S, Iruzubieta J, Gil-De-Gõmez J. General knowledge and opinion of future health care and non-health care professionals on rare diseases. J Eval Clin Pract. 2015;21.
87. Melo DG, de Paula PK, de Araujo Rodrigues S, da Silva de Avó LR, Germano CMR, Demarzo MMP. Genetics in primary health care and the National Policy on Comprehensive Care for People with Rare Diseases in Brazil: opportunities and challenges for professional education. J Community Genet. 2015;6.
88. Tosi LL, Oetgen ME, Floor MK, Huber MB, Kennelly AM, McCarter RJ, et al. Initial report of the osteogenesis imperfecta adult natural history initiative. Orphanet J Rare Dis. 2015;10.
89. Oliveira P, Zejnilovic L, Canhão H, Von Hippel E. Innovation by patients with rare diseases and chronic needs. Orphanet J Rare Dis. 2015;10.
90. Shin DW, Cho J, Yang HK, Kim SY, Lee SH, Suh B, et al. Oncologist perspectives on rare cancer care: A nationwide survey. Cancer Res Treat. 2015;47.
91. Samoutis G, Samoutis A. Paget Disease of Bone in Cyprus: Prevalence, Clinical Characteristics and Physicians’ Awareness. Int J Caring Sci [Internet]. 2015 [cited 2021 Aug 28];8:36–44. Available from: http://www.internationaljournalofcaringsciences.org/docs/5-Original Samoutis.pdf
92. Forsythe LP, Frank L, Walker KO, Anise A, Wegener N, Weisman H, et al. Patient and clinician views on comparative effectiveness research and engagement in research. J Comp Eff Res. 2015;4.
93. Johnson NE, Sowden J, Dilek N, Eichinger K, Burns J, Mcdermott MP, et al. Prospective study of muscle cramps in Charcot-Marie-Tooth disease. Muscle and Nerve. 2015;51.
94. Houyez F, Sanchez de Vega R, Brignol TN, Mazzucato M, Polizzi A. A European Network of Email and Telephone Help Lines Providing Information and Support on Rare Diseases: Results From a 1-Month Activity Survey. Interact J Med Res. 2014;3.
95. McClain MR, Cooley WC, Keirns T, Smith A. A survey of the preferences of primary care physicians regarding the comanagement with specialists of children with rare or complex conditions. Clin. Pediatr. (Phila). 2014.
96. Hendriksz CJ, Lavery C, Coker M, Ucar SK, Jain M, Bell L, et al. Burden of disease in patients with Morquio A syndrome: Results from an international patient-reported outcomes survey. Orphanet J. Rare Dis. 2014.
97. Smorti M, Cappelli F, Guarnieri S, Bergesio F, Perfetto F. Depression and cardiac symptoms among AL amyloidosis patients: The mediating role of coping strategies. Psychol Heal Med. 2014;19.
98. Johnson NE, Heatwole CR, Dilek N, Sowden J, Kirk CA, Shereff D, et al. Quality-of-life in Charcot-Marie-Tooth disease: The patient’s perspective. Neuromuscul Disord. 2014;24.
99. Greulich T, Ottaviani S, Bals R, Lepper PM, Vogelmeier C, Luisetti M, et al. Alpha1-antitrypsin deficiency - Diagnostic testing and disease awareness in Germany and Italy. Respir Med. 2013;107.
100. Zhou Q, Weis E, Ye M, Benjaminy S, MacDonald IM. An internet-based health survey on the co-morbidities of choroideremia patients. Ophthalmic Physiol Opt. 2013;33.
101. Zurynski Y, Deverell M, Dalkeith T, Johnson S, Christodoulou J, Leonard H, et al. Australian children living with rare diseases: experiences of diagnosis and perceived consequences of diagnostic delays. Orphanet J Rare Dis. 2017;12.
102. Calvert M, Pall H, Hoppitt T, Eaton B, Savill E, Sackley C. Health-related quality of life and supportive care in patients with rare long-term neurological conditions. Qual Life Res. 2013;22.
103. Desser AS. Prioritizing treatment of rare diseases: A survey of preferences ofNorwegian doctors. Soc Sci Med. 2013;94.
104. Tozzi AE, Mingarelli R, Agricola E, Gonfiantini M, Pandolfi E, Carloni E, et al. The internet user profile of Italian families of patients with rare diseases: A web survey. Orphanet J. Rare Dis. 2013.
105. Motil KJ, Caeg E, Barrish JO, Geerts S, Lane JB, Percy AK, et al. Gastrointestinal and nutritional problems occur frequently throughout life in girls and women with rett syndrome. J Pediatr Gastroenterol Nutr. 2012;55.
106. Carpenter DM, Blalock SJ, DeVellis RF. Patients with rare diseases using pharmacists for medication information. J Am Pharm Assoc. 2012;52.
107. Henschke C. Provision and financing of assistive technology devices in Germany: A bureaucratic odyssey? The case of amyotrophic lateral sclerosis and Duchenne muscular dystrophy. Health Policy (New York). 2012;105.
108. Zhao H, Cui Y, Zhou X, Pang J, Xu S, Han J, et al. Study and Analysis of the State of Rare Disease Research in Shandong Province, China. Intractable Rare Dis Res. 2012;1.
109. Wicks P, Massagli M, Frost J, Brownstein C, Okun S, Vaughan T, et al. Sharing health data for better outcomes on patientslikeme. J Med Internet Res. 2010;12.
110. Desser AS, Gyrd-Hansen D, Olsen JA, Grepperud S, Kristiansen IS. Societal views on orphan drugs: Cross sectional survey of Norwegians aged 40 to 67. BMJ. 2010;341.
111. Imrie J, Galani C, Gairy K, Lock K, Hunsche E. Cost of illness associated with Niemann-Pick disease type C in the UK. J Med Econ. 2009;12.
112. Abularrage CJ, Slidell MB, Sidawy AN, Kreishman P, Amdur RL, Arora S. Quality of life of patients with Takayasu’s arteritis. J Vasc Surg. 2008;47.
113. Galéra C, Delrue MA, Goizet C, Etchegoyhen K, Taupiac E, Sigaudy S, et al. Behavioral and temperamental features of children with Costello syndrome. Am J Med Genet. 2006;140 A.
114. Pasculli G, Resta F, Guastamacchia E, Di Gennaro L, Suppressa P, Sabbà C. Health-related quality of life in a rare disease: Hereditary hemorrhagic telangiectasia (HHT) or Rendu-Osler-Weber Disease. Qual Life Res. 2004;13.
115. Robey KL, Reck JF, Giacomini KD, Barabas G, Eddey GE. Modes and patterns of self-mutilation in persons with Lesch-Nyhan disease. Dev Med Child Neurol. 2003;45.
